# A genome-wide identification of the miRNAome in response to salinity stress in date palm (*Phoenix dactylifera* L.)

**DOI:** 10.3389/fpls.2015.00946

**Published:** 2015-11-05

**Authors:** Mahmoud W. Yaish, Ramanjulu Sunkar, Yun Zheng, Bo Ji, Rashid Al-Yahyai, Sardar A. Farooq

**Affiliations:** ^1^Department of Biology, College of Science, Sultan Qaboos UniversityMuscat, Oman; ^2^Department of Biochemistry and Molecular Biology, Oklahoma State UniversityStillwater, OK, USA; ^3^Faculty of Life Science and Technology, Kunming University of Science and TechnologyKunming, China; ^4^Department of Crop Science, College of Agriculture, Sultan Qaboos UniversityMuscat, Oman

**Keywords:** miRNA, salinity, genomics, date palm, *Phoenix dactylifera* L.

## Abstract

Although date palm is relatively salt-tolerant, little is known about the underlying molecular mechanisms that contribute to its salt tolerance. Only recently, investigators have uncovered microRNA-mediated post-transcriptional gene regulation, which is critical for typical plant development and adaptation to stress conditions such as salinity. To identify conserved and novel miRNAs in date palm and to characterize miRNAs that could play a role in salt tolerance, we have generated sRNA libraries from the leaves and roots of NaCl-treated and untreated seedlings of date palm. Deep sequencing of these four sRNA libraries yielded approximately 251 million reads. The bioinformatics analysis has identified 153 homologs of conserved miRNAs, 89 miRNA variants, and 180 putative novel miRNAs in date palm. Expression profiles under salinity revealed differential regulation of some miRNAs in date palm. In leaves, 54 of the identified miRNAs were significantly affected and the majority (70%) of them were upregulated, whereas in roots, 25 of the identified miRNAs were significantly affected and 76% of them were upregulated by the salinity stress. The salt-responsiveness of some of these miRNAs was further validated using semi-quantitative PCR (qPCR). Some of the predicted targets for the identified miRNA include genes with known functions in plant salt tolerance, such as potassium channel AKT2-like proteins, vacuolar protein sorting-associated protein, calcium-dependent and mitogen-activated proteins. As one of the first cultivated trees in the world that can tolerate a wide range of abiotic stresses, date palm contains a large population of conserved and non-conserved miRNAs that function at the post-transcriptional level. This study provided insights into miRNA-mediated gene expression that are important for adaptation to salinity in date palms.

## Introduction

In recent decades, excessive soil salinity has become a global agricultural constraint (Munns and Tester, 2008; Peleg et al., 2011). High evaporation rates from the soil surface coupled with the shortage of precipitation in hot and dry regions exacerbates the salinity problem. Plant species vary in their response to saline conditions; salt-tolerant plants use diverse morpho-physio-molecular strategies to cope with salinity, while susceptible plants suffer from ionic toxicity, osmotic and oxidative stresses, and even plant death (Bartels and Sunkar, 2005; Munns and Tester, 2008; Zhang and Shi, 2013).

Date palm, an economically important perennial plantation crop, is grown in various dry and semidry regions. It is a relatively salt-tolerant species with an adaptive capacity that exceeds barley, which is one of the most tolerant crops (Furr and Armstrong, 1975; Yaish, 2015; Yaish and Kumar, 2015). Ramoliya and Pandey (2003) screened date palm varieties for their salt tolerance and found that certain varieties can endure a relatively high level of soil salinity (12.8 dS m^−1^) with no visible effect. Although the date palm is a salt-adaptive plant, excessive salt in the soil beyond its capacity leads to significant damage. The underlying molecular mechanism associated with its salt tolerance has not been substantially investigated in the date palm.

In plants, microRNAs (miRNAs) represent both highly conserved ancient miRNAs as well as lineage- and species-specific young miRNAs (Sun, 2012). Genes that encode MIRNAs are transcribed by RNA polymerase II, and the resulting primary MIRNA transcript adopts a fold-back structure. With the assistance of HYPONASTIC LEAVES 1 (HYL1), SERRATE and other proteins, DICER-LIKE 1 enzyme (DCL1) in the nucleus recognizes such typical structures and endonucleolytically releases the 21-nt long miR-3p/miR-5p duplex from the fold-back structure (Kurihara et al., 2006). The duplex is then translocated to the cytoplasm by HASTY (HST) where usually one of the strands is functional (either the 5p or 3p) is integrated into the Argonaute (AGO) family of proteins called RNA-INDUCED SILENCING COMPLEX (RISC), to act on the mRNA targets (Bartel, 2004; Hu et al., 2014). MicroRNAs use sequence complementarity to find their messenger RNA (mRNA) targets to regulate their protein production by mediating cleavage or inhibiting translation (Llave et al., 2002; Bartel, 2004, 2009; Shukla et al., 2011). It was proposed that an miRNA can act as a threshold-based gene expression coordinator for its targets, similar to a buffer-based mechanism (Levine et al., 2007; Mukherji et al., 2011). Recently, it was discovered that ancient primary miRNA transcripts that evade processing by the Dicer in the cytoplasm could undergo translation to produce small peptides called “miPEPs” which in turn boost the transcription of their own primary miRNA transcript (Lauressergues et al., 2015).

In addition to miRNAs, the plant small RNA population is enriched with the other endogenous small interfering RNA (siRNA) molecules, especially 24-long small RNAs that are also called “heterochromatic siRNAs,” which can trigger and modulate *de novo* methylation of target loci through a process called RNA-directed DNA methylation (RdDM) (Wassenegger et al., 1994; Chan et al., 2006; Dalakouras and Wassenegger, 2013). The RdDM pathway can suppress the transcription of the target locus.

There is growing evidence that miRNAs are significantly important for plant adaptation to stress conditions (Sunkar et al., 2007, 2012), including salinity (Covarrubias and Reyes, 2010). Although none of the identified targets for the conserved miRNAs are known to be directly associated with the salt tolerance mechanisms in plants, most of the conserved miRNAs that target transcription factors could in turn regulate genes that play a role in salt tolerance (Sunkar et al., 2012). For example, the expression of several miRNAs, such as miR396, miR168, miR167, miR165, miR319, miR159, miR394, miR156, miR393, miR171, and miR158, has been altered in response to salinity and consequently affects the expression of their targets in Arabidopsis (Liu et al., 2008) and rice (Zhao et al., 2009). Such target gene regulation could be part of the plants' salt tolerance mechanism to upregulate important genes and/or repress unnecessary genes.

Recently small RNA population from the developing date palm fruits was sequenced, which led to the identification of some of the conserved miRNAs (Xin et al., 2015). Prior to that 18 conserved miRNA families have been predicted in the date palm (Xiao et al., 2013) based on the sequenced date palm genome (Al-Dous et al., 2011). The present study was undertaken to (i) identify conserved and novel miRNAs expressed in leaves and roots of date palm, and (ii) characterize salt-regulated miRNAs by profiling small RNAs. By sequencing 4 small RNA libraries, we have uncovered a total of 153 homologs of conserved miRNAs, 89 variants, and 180 putative novel miRNAs in date palm. Comparative miRNA profiling under salt stress revealed differential expression of most miRNAs, which suggests their potential involvement in the salt tolerance of this plant species.

## Materials and methods

### Plant growth conditions and salt treatment

Date palm seeds (*Phoenix dactylifera* L., cultivar *Khalas*) were thoroughly washed with tap water and surface sterilized by dipping in 75% ethanol for 3 min followed by 1.0% sodium hypochlorite solution for an additional 3 min. After rinsing 4 times with sterile water, the seeds were soaked overnight in water. The next day, the seeds were mixed with sterilized moist vermiculite and incubated at 30°C for 1 week in the dark. Subsequently, the germinated seeds transplanted in 2-L pots containing a mixture of vermiculite and peat moss (2:1). The pots were incubated in a growth chamber programmed for 16/8 h light/dark cycle with 350 μE m^−2^ s^−1^ light intensity and 35/30°C day/night temperature with 60% humidity. The plants were irrigated to field capacity with distilled water as needed for 5 more weeks. At the end of this period, the seedlings were either watered regularly (control treatment) or treated with a 300-mM NaCl solution at 72-h intervals (salinity treatment). A week after the beginning of the NaCl treatment, the leaves and roots of the control and treated seedlings were collected separately; thoroughly rinsed with tap water; dried with tissue paper and flash frozen in liquid nitrogen.

### miRNA extraction, library construction, and deep RNA sequencing

Leaf and root tissues from 6 control and salt-treated seedlings were separately pooled, and small RNAs were isolated using the miRNA Mini Kit (Qiagen). The quality and quantity of the RNA samples were tested using an Agilent 2100 Bioanalyzer (Agilent). The small RNAs were ligated with 3′ and 5′ adaptors, and RT-PCR was performed using the TruSeq™ Small RNA kit (Illumina) according to the manufacturer's instructions. The expected final PCR product was isolated from the gel, purified and sequenced using Hiseq2000 sequencer (Illumina). sRNA sequencing and basic bioinformatics analyses were conducted at Macrogen, Korea and LC Sciences, Texas, USA, respectively.

### Data analysis

The SolexaQA software package (Solexa) was used to calculate the quality statistics and create visual representations of the data quality from FASTQ files generated by the Illumina sequencer. The generated data files from each of the four libraries were used for the subsequent analysis. After removing the adaptor sequences from the small RNA reads, total and unique read counts for the small RNAs were established. From these, small RNAs that mapped to rRNA, tRNA, snoRNA, and snRNA were discarded, and the remaining reads were mapped to the assembled date palm genome (PDK_30) and the transcripts that are available at the Genbank of the National Library of Medicine (NLM), USA (http://ftp.ncbi.nlm.nih.gov), and the Laboratoire de Biogenese Membranaire, France (http://www.biomemb.cnrs.fr/date_contigs.doc) using the Bowtie software (Langmead et al., 2009). For alignment using Bowtie, one mismatch in the first 16 nt was allowed. Unmapped reads were excluded from further analysis.

To the genome matching reads, hairpin-like structures were predicted using the UNAFold software (Markham and Zuker, 2008) with the following default settings: the number of permitted errors in one bulge in stem ≤ 12, the number of base pairs (bp) in the stem region ≥ 16, free energy (ΔG in kCal/mol) ≤ −15, length of the hairpin (up and down stem + terminal loop) ≥50, length of the terminal loop ≤ 350, number of allowed errors in one bulge in a mature region ≤ 8, number of allowed biased errors in one bulge in mature regions ≤ 4, number of allowed biased bulges in mature regions ≤ 2, number of base pairs (bp) in mature 5p-miRNAs or mature 3p-miRNA^*^ region ≥ 12, percentage of small RNAs in stem regions (pm) ≥ 80% and number of allowed errors in mature regions ≤ 7. sRNAs were further filtered using a more stringent strategy (Körbes et al., 2012), which includes at least 10 miRNA reads that originated from a stem-looped hairpin with a high negative minimum folding free energy (MFE ≤ −40 kcal mol^−1^) and a minimum folding free energy index (MFEI) higher than 0.85 (Zhang et al., 2006b) were considered for further analysis.

### Identification and analysis of conserved and novel miRNAs

To identify conserved miRNAs, sRNAs were aligned with known non-redundant plant mature miRNAs that were available in the miRBase using Bowtie v 0.12.7. The miRNAs with a complete match sequence were considered to be conserved sequences. The miRNAs with 1–3 mismatches were considered to be miRNA variants, and the miRNAs with no similarity to those in the miRNA database were considered to be novel miRNAs, as previously classified by Ma et al. (2013). The length of the miRNA precursor sequences identified in this study ranges from 57 to 264 bp (Tables S1–S3). The formation of secondary hairpin structure and the alignment of putative novel miRNA sequences on the corresponding precursor were verified using the UAE small RNA workbench software (Stocks et al., 2012).

### Differential gene expression

Normalization of sequence counts in each miRNA was calculated by dividing the counts by a library size parameter of the corresponding sample following the previously described method (Anders and Huber, 2010). The library size parameter was considered to be the median value of the ratio between the counts of a specific sample and a pseudo-reference sample, which was calculated based on the following equation:

$$s_{j}=\underset{i}{\text{median}}\;\left[\frac{c_{ij}}{{\left(\prod_{k=1}^{m}\;c_{ik}\right)}^{1/m}}\right]$$

given that *S*_*j*_ is the library size parameter; *c*_*ij*_ is the count number of sequence *i* of sample *j*; and *m* is the total number of samples associated with the analysis.

After removing the miRNA sequences that did not show read counts in the libraries, 355 miRNA unique sequences were found in the four libraries and were used to study the expression level of the miRNA based on the normalized read count using the Differential Expression Analysis of Digital Gene Expression Data software (edgeR) (Chen et al., 2014). The Biological coefficient of variation (BCV) value was set to 0.2 according to the software's instructions. Significantly regulated miRNAs were predicted if multiple test corrected *P*-value and the false discovery rate (FDR) is < 0.05. The complete linkage of the hierarchical clustering analysis and the visualization of the normalized expression values were obtained using PermutMatrix software (Caraux and Pinloche, 2005).

### Computational prediction of miRNA targets

Prediction of target genes for the conserved, variants and putative novel miRNA sequenced from the four libraries was accomplished by searching the miRNA against preloaded assembled RNA-seq contigs using psRNAtarget software (Dai and Zhao, 2011), which is available online at http://plantgrn.noble.org/psRNATarget/. We used the following default parameters: maximum expectation (ME) at 3, length for complementarity scoring (hspsize) at 20, target accessibility-allowed maximum energy to unpair the target site (UPE) at 25, flanking length around the target site for target accessibility analysis at 17 bp in upstream and 13 bp in downstream and the range of central mismatch that leads to translational inhibition between 9 and 11 nt.

Potential mRNA targets were then annotated, and each was assigned a function using blast2GO v2.3.5 software (Conesa et al., 2005) based on deduced protein similarities with other deposited sequences and conserved domains available at Swiss- Prot/Uniprot protein databases using BLASTx. Each sequence was assigned one Gene Ontology (GO) term or more for the cellular component, molecular function and biological processes. Mapping of the target genes on known metabolic pathways was conducted using the Kyoto Encyclopedia of Genes and Genomes (KEGG; http://www.genome.jp/kegg) (Kanehisa et al., 2004), implanted within the blast2GO v2.3.5 software. Venn Diagrams were generated using the VENNY software (Oliveros, 2007), which was available at http://bioinfogp.cnb.csic.es/tools/venny/index.html.

### Quantitative real-time PCR (RT-qPCR)

The read count of selected miRNAs was verified using the stem-loop Quantitative Real-time PCR (RT-qPCR) method, as previously described by Chen et al. (2005). Date palm seedlings were treated with a saline solution as previously described in this report. Leaf and root samples of five NaCl-treated and control seedlings were separately pooled and homogenized in liquid nitrogen, and the total RNA was extracted using the mirVana™ miRNA Isolation Kit (Life Technologies). After DNase treatment, the cDNA of the premature miRNA was synthesized using the NCode™ VILO™ miRNA cDNA Synthesis Kit, and RT-qPCR was performed using the NCode™ EXPRESS SYBR® GreenER™ miRNA kit (Life Technologies). DNA sequence of each miRNA was used as a forward primer in combination with a universal reverse primer which was included within the NCode™ EXPRESS SYBR® GreenER™ miRNA kit. The U6 small nuclear ribonucleic acid (snRNA) (5′-CCTGCGCAAGGATGACACGCAT-3′), sequence was used as a housekeeping normalization miRNA sequence, as previously described (Jain et al., 2014; Xie et al., 2015). The gene expression was calculated based on the 2^−Δ*ΔC*^T method (Livak and Schmittgen, 2001) and the average fold of control was calculated based on 3 independent biological and 2 experimental replicates. The Raw sRNA sequences were deposited in the GenBank/NCBI GEO under the accession number GSE71445.

## Results and discussion

### Salt treatment

The date palm is a relatively salt-tolerant tree species (Furr and Armstrong, 1975; Ramoliya and Pandey, 2003), but the underlying physiological or molecular mechanisms associated with salt tolerance are unknown. The ability to adapt to saline conditions is largely dependent on the induction/suppression of many genes upon exposure to salinity. Such a change in gene expression could be partly controlled by the miRNAs in plants (Covarrubias and Reyes, 2010; Yaish et al., 2011).

The average soil salinity measured as the electrical conductivity level in pots at the beginning of the experiment was 0.68 ds m^−1^. After treatment twice with 300 mM NaCl solution for 1 week, the average electrical conductivity of the soil in the treated and control pots was 17.46 and 0.64 ds m^−1^, respectively. The soil salinity levels were relatively high and could have caused a significant osmotic pressure on the plants; however, phenotypic differences were not obvious between the treated and untreated plants (control plants), which can be attributed to the short duration of salt exposure coupled with the anatomical features of the date palm leaves such as the presence of a thick layer of wax (Yaish, 2015). The salinity treatment used in this experiment could shock the seedlings and dramatically alter the gene expression that is associated with salt tolerance (Shavrukov, 2013), and the salinity levels used in this report were comparable to those used in other reports (Ding et al., 2009; Long et al., 2014; Zhuang et al., 2014). This salinity shock could explain the reason behind the dramatic change in miRNA read count in the date palm seedlings.

### miRNA sequencing revealed profile diversity among sRNA libraries

Being major post-transcriptional regulators, miRNAs have been shown to respond to salt stress in several plant species (Kumar, 2014; Peng et al., 2014; Tian et al., 2014). Because of direct physical contact with saline soil, the root is the first organ to be affected during salt stress. With an increasing duration of salt stress, the shoots are also affected due to Na^+^ and/or Cl^−^ toxicity (Tester and Davenport, 2003; Møller and Tester, 2007). However, the ion toxicity in the shoots could be diminished by decreasing the salt translocation to the shoots, or the shoots can effectively tolerate salt accumulation through pumping into vacuoles and/or tissue tolerance (Munns and Tester, 2008). Therefore, it is important to study the effect of salt stress on both organs (roots and leaves) of date palm.

To identify the miRNA repertoire in date palm and to reveal the changes in the miRNA profile during salt stress, sRNA libraries were constructed from the roots and leaves of seedlings exposed to NaCl and control conditions and were sequenced using the Illumina platform, a powerful sequencing technology. This approach yielded almost 251 million total reads and approximately 26 million unique reads from the four sRNA libraries. After eliminating low-quality sequences, approximately 232 million total reads and 19.5 million unique reads, which ranged between 18 and 25 nucleotides, were obtained. Of these, only 5,196,547 unique sRNA reads could be mapped to the date palm genome, of which there were 4470 and 1,682,804 unique and redundant mappable reads, respectively (Table 1), which suggests that the majority of the unique small RNA reads could not be used in the analysis. This limitation likely due to incomplete genome sequencing or assembly or due to RNA contamination with other small RNA molecules from endophytic microorganisms harboring date palm roots and leaves (Yaish et al., 2015). The estimated genome size of the date palm is approximately 658 Mb (Al-Dous et al., 2011); however, the assembled sequences that are available in the Gene Bank cover 381.563 Mb only. A total of 27,368 known (previously sequenced) and 14,537 predicted sRNA unique reads were sequenced from the four libraries.

**Table 1:** **Summary of sequencing data, presented as reads abundance of ***P. dactylifera*** small RNA libraries**.

	**Redundant reads**					**Unique reads**				
	**Control-leaves**	**NaCl-leaves**	**Control-root**	**NaCl-root**	**Total**	**Control-leaves**	**NaCl-leaves**	**Control-root**	**NaCl-root**	**Total**
Total reads	69,820,252	57,749,080	53,893,175	70,138,817	251,601,324	5,488,113	7,354,391	6,018,618	7,192,892	26,054,014
Total reads after filtration	67,941,166	53,269,570	47,716,479	63,423,863	232,351,078	4,110,970	5,632,666	4,534,884	5,235,066	19,513,586
Total mapped small RNAs	48,597,554	39,388,275	25,753,815	34,829,777	148,569,421 (1,682,804)^*^	1,070,750	1,405,067	1,209,183	1,511,547	5,196,547 (4,470)^*^
Total known small RNAs	26,439,310	19,827,140	18,686,729	24,574,312	89,527,491	348,574	361,770	443,727	429,316	1,583,387
Known miRNA	376,803	591,326	224,471	347,865	1,540,465	5,176	10,171	6,194	5,827	27,368
Predicted miRNA	314,899	903,299	36,325	73,887	1,328,410	4,434	4,410	2,622	3,071	14,537
rRNA	19,241,412	8,896,131	14,891,035	20,437,750	63,466,328	263,646	210,394	337,115	332,132	1,143,287
tRNA	3,090,812	6,156,880	1,293,910	842,345	11,383,947	32,510	47,369	40,966	33,476	154,321
snoRNA	613,764	696,722	429,123	352,090	2,091,699	35,143	64,141	42,891	41,814	183,989
snRNA	106,920	238,688	167,258	159,847	672,713	12,099	29,695	16,561	16,067	74,422
no hit	19,343,612	13,881,295	21,962,664	28,594,086	83,781,657	3,040,220	4,227,599	3,325,701	3,723,519	14,317,039

* *Unique and redundant reads of size ranging between 18 and 25 nt*.

**Figure 1:**
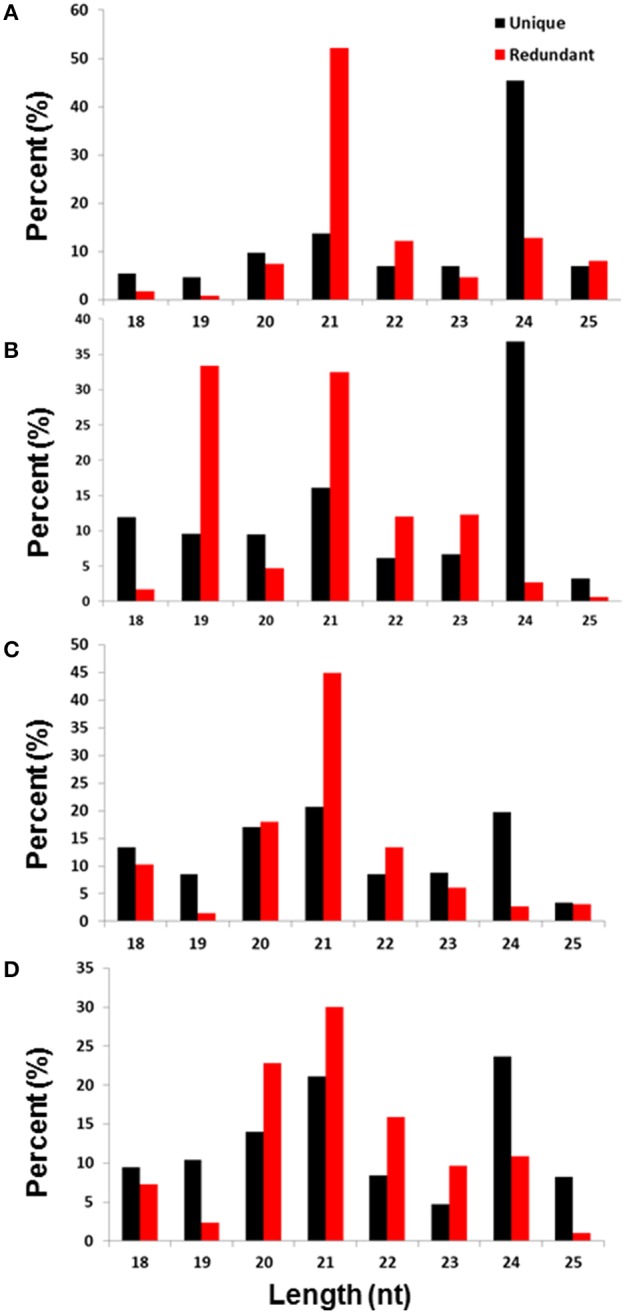
**Size distribution in the base pair (bp) and diversity percentage of the total and unique small RNA reads isolated from untreated leaves (A), NaCl-treated leaves (B), untreated roots (C), and NaCl-treated roots (D)**.

Consistent with the length distribution patterns of the small RNAs isolated from other plant species (Rajagopalan et al., 2006; Zhu et al., 2008), the sRNA of 21 and 24 nt sizes were the most representative in all four sRNA libraries from the date palm (Figure 1). As expected, the redundant reads that belong to 21 nt were greater in numbers compared to the unique reads of this class, which suggests that this class could represent miRNAs. In contrast, the unique reads of the 24 nt size class were greater, and the redundant reads were much smaller. The small RNAs that belong to the 24-nt size class were the most representative in three of the four libraries (Figures 1A,B,D), and the only exception was the untreated root sample, in which this size was the second most representative (Figure 1C). The 21-nt size class was the most abundant fraction in the four libraries, except for the library generated from the NaCl-treated leaves (Figure 1B), where the 19- and 21-nt fraction appeared as dual peaks (Figure 1). Compared to the untreated leaves, the percentage of redundant reads that belong to the 19-nt class was much greater in the salt-treated leaves (more than 30% in salt but less than 5% in control), while the percentage of redundant reads that belong to the 24-nt class was much lower during salt stress. Compared to the untreated roots, the percentages of redundant reads that belong to the 21-nt size have decreased, while the unique reads were unaltered during salt stress. Similarly, the percentage of redundant reads that belong to the 20-nt size was similar between the control and treated samples, whereas the unique reads percentage increased. Interestingly, the percentage of redundant reads of the 24-nt size class was increased in the roots but decreased in the leaves under salt stress. This finding suggested that there was differential regulation of small RNA loci in roots and leaves, which could be due to differential activation of repeat-rich regions/transposons that are potential loci for generating 24-nt-long small RNAs (Figure 1).

The functional sRNA that is produced in plants is mainly composed of either 21- or 24-nt sRNAs. While the 21-nt sRNAs are largely miRNAs that are transcribed by RNA polymerase II and are essential for mRNA decay, the 24-nt sRNAs are produced by the PolIV/Pol V system and play an important role in RNA-directed DNA methylation (Law et al., 2010) and subsequent histone modifications at the target chromatin (Matzke et al., 2009). Recent studies have showen that the most abundant sRNAs found in the angiosperm tissues are 24-nucleotide Pol IV–dependent siRNAs, which are denoted as p4-siRNAs (Ma et al., 2010). These p4-siRNAs are derived from the DCL processing of long, perfectly base-paired double-stranded RNA precursors.

### sRNAs sequencing uncovers conserved miRNAs and miRNA variants in date palm

sRNA sequences that were mapped to the genome (with a maximum 1-nt mismatch) were used for the identification of conserved and novel miRNAs, by searching against plant miRNA sequences in the miRBase. The sRNA sequences were classified based on their degree of similarity with the previously identified miRNA sequences in the miRBase. The date palm miRNAs that were identical (100% similarity) were considered to be conserved miRNA (Table 2, Table S1), and those with one or two mismatches to those in the miRBase were considered to be miRNA variants (Table S2), while those with no similarity were considered to be potential novel miRNA sequences (Table 3, Table S3). Similar classification criteria were previously used by Ma et al. (2013).

**Table 2:** **Conserved miRNAs in ***P. dactylifera*** L**.

**5p-miRNA/3p-miRNA**	**Sequence(5′-3′)**	**nt**	**ΔG**	**Total Reads**
pda-miR156a	UGACAGAAGAGAGUGAGCU	19	−60.7	448
pda-miR156i-3p	GCUCACUUCUCUUUCUGUCAGCC	23	−60.7	25
pda-miR156a	UGACAGAAGAGAGUGAGCAC	20	−65.7	11,263
pda-miR156i-3p	GCUCACUUCUCUUUCUGUCAGCC	23	−65.7	25
pda-miR156a	UGACAGAAGAGAGUGAGCAC	20	−50.4	10,790
pda-miR156i-3p	GCCCACUUCUCUUUCUGUCAA	21	−50.4	1
pda-miR156a	CUGACAGAAGAGAGUGAGC	19	−57.0	370
pda-miR156i-3p	GCUCACUUCUCUUUCUGUCAGC	22	−57.0	623
pda-miR156h	UUGACAGAAGAUAGAGAGCAC	21	−53.1	14,392
pda-MIR156h-p3	GCUCUCUAUGCUUCUGUCAUC	21	−53.1	1158
pda-miR156e	UUGACAGAAGAUAGAGAGCAC	21	−41.1	13,737
pda-MIR156c-p3	GCUCUCUAUGCUUCUGUCAUU	21	−48.6	2
pda-miR160a-5p	UGCCUGGCUCCCUGUAUGCCA	21	−48.7	911
pda-miR160a-3p	GCGUGCAAGGAGCCAAGCAU	20	−48.7	2
pda-miR166a-5p	GGGAAUGUUGUCUGGUUCGAG	21	−63.9	37
pda-miR166a	UCGGACCAGGCUUCAUUCCCC	21	−63.9	159,897
pda-miR166a-5p	GGAAUGUUGUCUGGCUCGAGG	21	−65.0	566
pda-MIR166b-p3	UCUCGGACCAGGCUUCAUUC	20	−65.0	2015
pda-miR166a-5p	GGAAUGUUGUCUGGCUCGAGG	21	−63.2	359
pda-miR166i-3p	UCUCGGAUCAGGCUUCAUUCC	21	−63.2	9121
pda-miR166a-5p	GGAAUGUUGUCUGGUUC	17	−56.8	7
pda-miR166a	UCGGACCAGGCUUCAUUCC	19	−56.8	3400
pda-miR166i-5p	AAUGAGGUUUGAUCCAAGAUCU	22	−65.5	2
pda-miR166i-3p	UCUCGGAUCAGGCUUCAUUCC	21	−65.5	5803
pda-miR167a-	UGAAGCUGCCAGCAUGAUCUA	21	−65.9	61
pda-miR167b-3p	GGUCAUGCUCUGACAGCCUCACU	23	−65.9	1
pda-miR167a	UGAAGCUGCCAGCAUGAUC	19	−56.0	5212
pda-miR167b-3p	GGUCAUGCUCUGACAGCCUCACU	23	−56.0	1
pda-miR168a	UCGCUUGGUGCAGGUCGGGAACGG	24	−63.6	53
pda-miR168a-3p	CCCGCCUUGCAUCAACUGAA	20	−63.6	235
pda-miR168a	UCGCUUGGUGCAGGUCGGGA	20	−59.7	654
pda-miR168c-3p	CCCGCCUUGCAUCAACUGAA	20	−59.7	375
pda-miR168a-	UCGCUUGGUGCAGGUCGGGAAC	22	−65.3	26
pda-miR168c-3p	CCCGCCUUGCAUCAACUGAAU	21	−65.3	180
pda-miR168a	UCGCUUGGUGCAGGUCGGGA	20	−77.5	440
pda-miR168c-3p	CCCGACUUGCAUCAACUGA	19	−77.5	8
pda-miR169a	UAGCCAAGGAUGACUUGCCU	20	−70.3	94
pda-miR169j-3p	GGCAGUCUCCUUGGCUAGUC	20	−70.3	35
pda-miR169c-	UAGCCAAGGAUGAUCUGCCU	20	−78.4	85
pda-miR169e-3p	GGCAGUCUCCUUGGCUAGUA	20	−78.4	3
pda-miR169	UAGCCAAGGAUGACUUGCCU	20	−73.7	146
pda-miR169j-3p	GGCAGUCUCCUUGGCUAGUC	20	−73.7	55
pda-miR169p	CAGCCAAGGAUGACUUGCC	19	−49.0	494
pda-miR169a-3p	CGGCGAGUCUGUCCUUGGCUAC	22	−49.0	16
pda-miR169a	UAGCCAAGGAUGACUUGCCUU	21	−80.1	37
pda-miR169j-3p	GGGCAGUCUCCUUGGCUAGUC	21	−80.1	1
pda-miR172b-5p	UGGCAUCAUCAAGAUUCACAU	21	−61.0	1
pda-miR172a	GGAAUCUUGAUGAUGCUGCA	20	−61.0	11
pda-miR390a	AAGCUCAGGAGGGAUAGCGCC	21	−63.8	11
pda-miR390a-3p	CGCUAUCUAUCCUGAGUUU	19	−63.8	11
pda-miR390a	AAGCUCAGGAGGGAUAGCGCC	21	−63.1	11
pda-miR390a-3p	CGCUAUCUAUCCUGAGCA	18	−63.1	4
pda-miR393a	UCCAAAGGGAUCGCAUUGAUC	21	−57.9	143
pda-miR393b-3p	AUCAUGCGAUCCUUUUGGAU	20	−57.9	2
pda-miR393a	UCCAAAGGGAUCGCAUUGAUCU	22	−54.5	517
pda-miR393b-3p	AUCAUGCGAUCCUUUUGGAU	20	−54.5	2
pda-miR393d	AUCAUGCGAUUCCUU	15	−50.3	28
pda-miR393a	UCCAAAGGGAUCGCAUUGAUUU	22	−50.3	2200
pda-miR394a-5p	UUGGCAUUCUGUCCACCUCC	20	−65.5	34
pda-MIR394a-p3	AGCUCUGUUGGCUUCUCUUUG	21	−65.5	11
pda-miR395a-5p	UUCCCUCAGACACUUCAUU	19	−52.3	1
pda-miR395	CUGAAGUGUUUGGGGGAACG	20	−52.3	147
pda-miR395a-5p	GUUCCCUCAGACACUUCAU	19	−62.7	155
pda-MIR395b-p3	AAGUGUUUGGGGGAACUCUAGG	22	−62.7	543
pda-miR395a-5p	GUUCCCUCAGACACUUC	17	−80.6	5
pda-miR395	CUGAAGUGUUUGGGGGAACU	20	−80.6	5317
pda-miR396a-5p	UCACAGGCUUUCUUGAACU	19	−56.9	3200
pda-miR396a-3p	GUUCAAGAAAGUCCG	15	−56.9	23
pda-miR396a-5p	ACAGCUUUCUUGAACCG	17	−57.3	2
pda-miR396g-3p	GGUCAAGAAAGCUGUGGAAG	20	−57.3	1
pda-miR396b	CAGCUUUCUUGAACUG	16	−47.0	22,642
pda-miR396a-3p	GUUCAAUAAAGCUGUGGGAAA	21	−47.0	148
pda-miR396	UUCCACGGCUUUCUUGAACU	20	−57.1	6208
pda-miR396g-3p	UCAAGAAAGACGUGGGAAAACA	22	−57.1	5

*Each pair of shaded rows represents a pair in a 5p-miRNA/3p-miRNA duplex of a single hairpin precursor*.

**Table 3:** **Novel 5p-miRNA/3p-miRNA libraries sequenced from untreated leaves (C-L), NaCl-treated leaves (Na-L), untreated roots (C-R), and NaCl-treated roots (Na-R)**.

**miRNA**				**Precursor**			**Number of reads**			
**5p-miRNA/3p-miRNA**	**Mature sequence (5′-3′)**	**nt**	**Strand**	**Genome location**	**MFE**	**MFEI**	**C-L**	**C-R**	**Na-L**	**Na-R**
pda-3p-117840_82-3p-R3	AGGCACUUAUCGCCGGGCUAAAGC	24	3′	769101	−217.9	1.6	17	0	86	6
pda-5p-85614_137-5p-R2	AGCUCCCUUGCCAGCUUUAGCCCG	24	5′	769101	−217.9	1.6	113	4	352	28
pda-3p-117840_82	AGGCACUUAUCGCCGGGCUAAAGC	24	3′	769101	−208.2	1.7	14	0	33	3
pda-5p-85614_137	AGCUCCCUUGCCAGCUUUAGCCCG	24	5′	769101	−208.2	1.7	78	3	274	24
pda-3p-130659_69	ACAAAGCUUUGGAGAACUGUU	21	3′	782981	−149.8	2	16	14	173	16
pda-5p-224392_29	CAGUUCCUCCAAAGCUUUGUA	21	5′	782981	−149.8	2	8	3	102	8
pda-3p-290154_20	UAAUGAUUAGUGGAACAGCGG	21	3′	688561	−137.9	1.8	7	1	21	2
pda-5p-83202_144	ACCAGUCCGAGAAUCGUUUCAAUC	24	5′	688561	−137.9	1.8	63	19	103	15
pda-3p-99813_107	AUUCGGGCCAGUCCACUCUAAAGC	24	3′	1013541	−115.5	1.6	31	0	76	0
pda-5p-295079_19	AGGAGCUUUGGAGUGAGCCGGCCC	24	5′	1013541	−115.5	1.6	8	0	11	0
pda-3p-117918_82	CGGAUGCUGCGCCUCGUGUA	20	3′	832241	−104.2	1	29	8	242	5
pda-5p-92754_121	CGUGUUCAGCAAGCUCUGGGACCC	24	5′	832241	−104.2	1	111	35	220	45
pda-3p-64863_213	UCUAUUCAAACAUUGACUGCU	21	3′	1147931	−100.8	1.2	113	29	652	35
pda-5p-152404_54	ACCGGCGGUAAGAUUUUGUAGG	22	5′	1147931	−100.8	1.2	34	2	134	12
pda-3p-313824_18	AGGAGUCGACUCCAGCUCUGCAGG	24	3′	930241	−100.6	1	3	3	18	5
pda-5p-321868_17	AGGGCUGGAGUCGACUCCUGCAGG	24	5′	930241	−100.6	1	2	1	12	2
pda-3p-107067_96	GCUGCCGGACUCUUGUUUCGAAGG	24	3′	991011	−90.8	1.3	46	1	47	2
pda-5p-311908_18	UGAAACAAGUGUUCGGCUGCU	21	5′	991011	−90.8	1.3	7	5	16	1
pda-3p-8373_3137	CUGUAAACCGCACGACCCUUC	21	3′	742991	−80.3	1	178	88	3426	33
pda-5p-396570_13	AGAGUCGUGCGGUCUACAGAG	21	5′	742991	−80.3	1	0	0	36	1
pda-3p-93572_119	CCUGUGCAUGCCUCCUCCACC	21	3′	799661	−75.4	0.9	0	5	128	5
pda-5p-85498_137	UGGAAGGGACAUGCAGAGGAA	21	5′	799661	−75.4	0.9	0	56	64	17
pda-3p-64787_213	GUGCUCUUUCUCGUUGUCACC	21	3′	715261	−74.2	0.9	31	10	265	12
pda-5p-178899_42	AGCACGCCGGUCGGCAGUCAUGCA	24	5′	715261	−74.2	0.9	12	6	27	3
pda-3p-21009_1073	UCUGUCCGGCACUGUUCAGCC	21	3′	1024561	−72.1	1.1	261	41	2262	89
pda-5p-367016_14	CCUUCUGUUCGGCUGCACAGUGCC	24	5′	1024561	−72.1	1.1	2	1	20	1
pda-3p-224491_29	AGGCUUCUGUUCGGCGAUCCAGUG	24	3′	1074861	−70.8	1.3	15	1	62	4
pda-5p-58294_251	UCCGUUCGGCACUGGAUCGCC	21	5′	1074861	−70.8	1.3	100	24	280	35
pda-3p-269912_22	ACUACUCUGCUGCGCUCAAUGAUC	24	3′	677511	−67.7	0.9	8	0	25	0
pda-5p-310639_18	UCAUUGGGCCUAGUAGACUAGUCC	24	5′	677511	−67.7	0.9	3	2	13	0
pda-3p-171233_45-3p-R16	UUCGGUCGUGCCACGCUGUGUGCGUC	26	3′	838941	−64.7	1.1	10	2	13	17
pda-5p-12958_1929	CGCGCAUGGCGUGGAACGAUCACC	24	5′	838941	−64.7	1.1	50	31	1504	704
pda-3p-44090_382	UUCAAGAAAGUCAGUGGAAG	20	3′	1094891	−56.9	1.1	76	69	499	42
pda-5p-15197_1588	UCACAGGCUUUCUUGAACUA	20	5′	1094891	−56.9	1.1	231	101	1675	34
pda-3p-138631_63	AGGACUGGGAUUCCUACU	18	3′	874461	−52.6	1.3	30	8	54	10
pda-5p-37236_491	ACUUCGAGUAGGAUUCCUAGAGUC	24	5′	874461	−52.6	1.3	214	2	268	7
pda-3p-231836_28	CAGAAGGCUUCUGUUCGGCACC	22	3′	919691	−48.6	1	6	2	31	2
pda-5p-131814_68	AACAAAAGGCUUCUGUUCGGC	21	5′	919691	−48.6	1	11	3	46	8
pda-3p-329752_16	AAACAGGUCAGACAUAUAAAACCC	24	3′	969931	−41.0	1	4	4	7	1
pda-5p-183654_40	ACCUGUUUAGACCUGUUUAGACCC	24	5′	969931	−41.0	1	16	0	20	4

*Each pair of shaded rows represents a pair in a 5p-miRNA/3p-miRNA duplex of a single hairpin precursor. The mature miRNA sequences shown were mapped on the chromosomes of the PDK_30s database. The negative minimum folding free energy (MFE) and minimum folding free energy index (MFEI) of the hairpin precursors are shown in the Table*.

**Figure 2:**
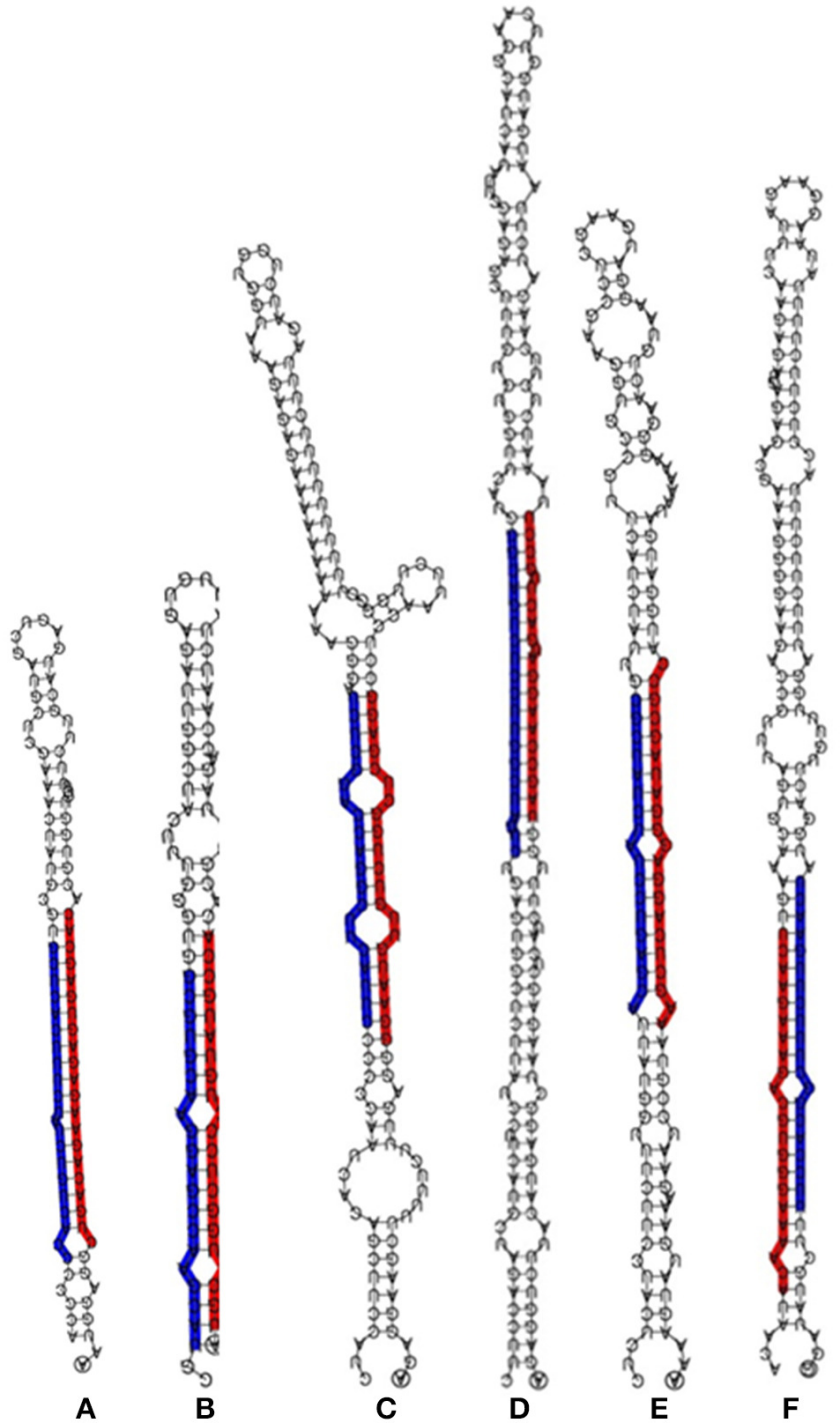
**Stem-loop RNA secondary structure of hairpin-forming precursors of some conserved miRNA sequenced from ***Phoenix dactylifera*** L**. The mature 5p-miRNA/3p-miRNA sequences are shaded in either red or blue. The stem loop precursors and 5p-miRNA/3p-miRNA represent miR156 **(A)**, miR160 **(B)**, miR166 **(C)**, miR169 **(D)**, miR390 **(E)**, and miR396 **(F)** sequences.

Together from the four libraries, a total of 153 unique conserved miRNA sequences, identical to the previously published miRNA sequences, were identified, of which 113, 149, 115, and 107 were specifically identified from the leaf-control, leaf-NaCl, root-control, and root-NaCl libraries, respectively (Table S1). Interestingly, the sequence analysis showed 38 with a complimentary miRNA sequence that originated from the same hairpin precursor and aligned on either the 5′ or 3′ arm of the hairpin precursor (Figure 2, Table 2 and Table S1). The conserved miRNA list shown in Table 2 comprises only those that have both 5p-miRNA and 3p-miRNA sequences. The most common length for the conserved miRNA sequences isolated form leaves and root is composed of 21 nt (Tables S1, S2), this is consistent with length distribution of the miRNA sequenced form date palm fruits at various developmental stages (Xin et al., 2015).

The expression levels measured as read counts in the majority of the paired miRNAs showed a significant difference between the sequences aligned on the 5′ and those aligned on the 3′ arm of the hairpin-like structure (Table 2 and Table S5). Of these duplexes, sequences with higher abundances are considered to be miRNAs. These miRNA are either located on the 5p or 3p end of the hairpin sequence. The presence of this combination within the sequenced sRNA libraries confirmed that these are true miRNAs.

**Figure 3:**
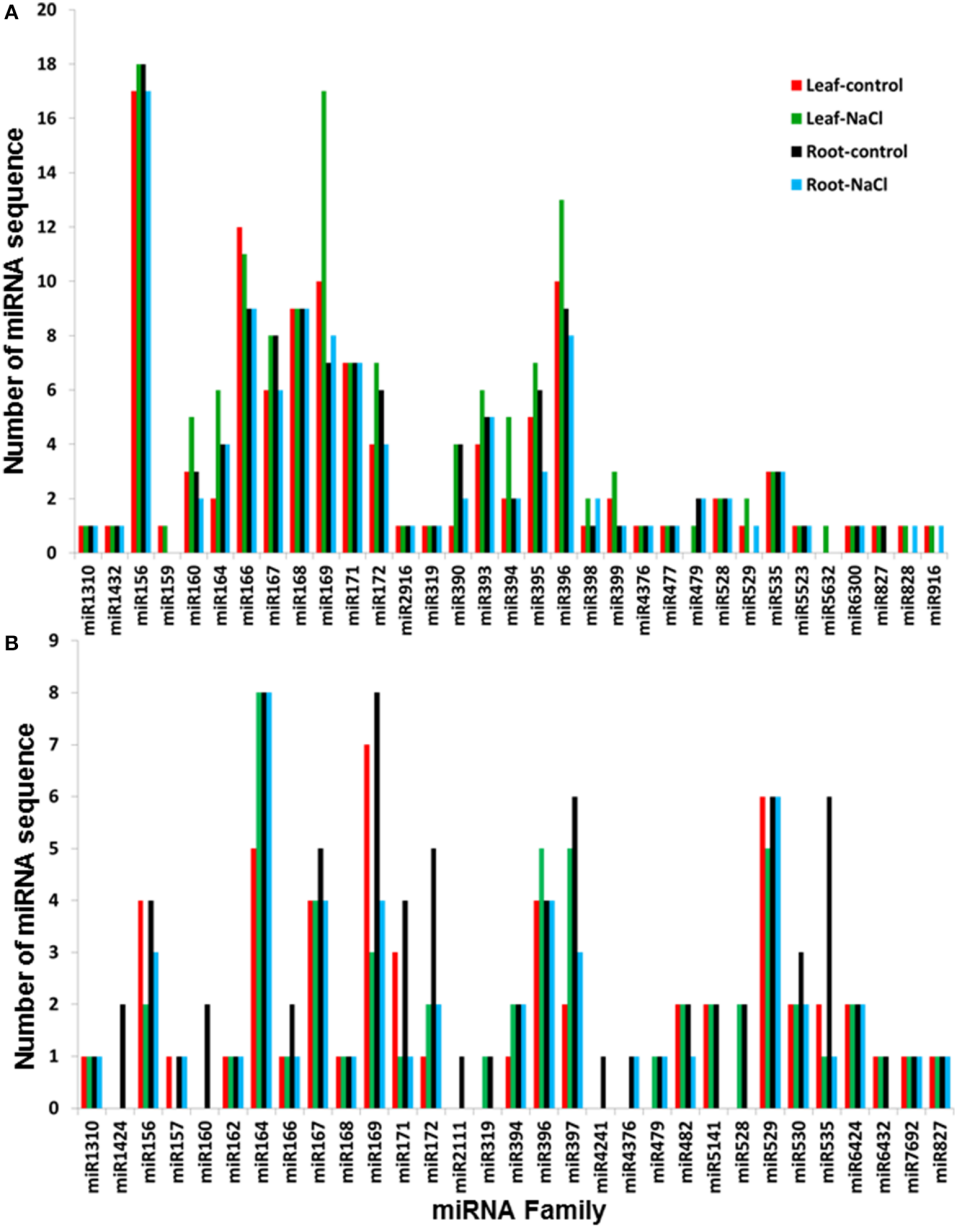
**Number of characterized miRNA sequences per plant miRNA family isolated from ***Phoenix dactylifera*** L. libraries and classified as conserved (A) and variants (B)**. The numbers represent the sum of the different miRNA sequences that comprise the conserved sequences of the families of plant miRNAs that were deposited in the miRBase.

BLAST analysis showed that the conserved miRNA sequences that were isolated in this study belong to 33 miRNA families, which were identified in 35 plant species available at the miRBase. The miR156, miR166, miR169, and miR396 families were the most diverse conserved miRNA families, with an average number of 17, 10, 10, and 10 miRNA variants per family, respectively (Figure 3A). Most of the conserved miRNA families were represented in the libraries that were generated from both the leaves and roots, and 26 of the 33 conserved miRNA families were common in the four libraries. Only three conserved miRNA sequences that belong to the miR169a and miR398a families were expressed in the NaCl-treated leaves and roots, while 15 conserved miRNA sequences were specifically expressed in leaves upon exposure to NaCl stress (Figure S2A, Table S4); no miRNA sequence was specifically expressed in NaCl-treated root tissue. miRNA-156 family was the most representative conserved sequence in roots and leaves libraries (Figure 3) however, this result is inconsistent with a previously reported miRNA-169 family form date palm fruits where miRNA-169 family is the most representative conserved sequence (Xin et al., 2015). Variation in miRNA expression could be due to the difference in the tissue type used in these studies.

The sRNA sequence similarity search against the miRBase showed the presence of 89 miRNA sequences that differed by 1 or 2 nt with the previously identified miRNA sequences at the miRbase (Table S2). The list of these variant miRNAs included 55, 87, 57, and 52 miRNA sequences that were identified from the leaf-control, leaf-NaCl, root-control, and root-NaCl libraries, respectively. This list also included 13 miRNAs with a sequenced a semi-complimentary miRNA sequence (Table S2). Sequence annotation revealed 31 variants, of which miR164, miR169, and miR529 were the most representative families among the libraries, with an average number of 7, 6, and 6 miRNA sequences per family, respectively (Figure 3B). Of the 31 families, 21 miRNA families were represented in the four sequenced libraries. Sequence analysis also showed the absence of any miRNA sequence that was specifically expressed in the NaCl-treated roots; however, miR164 and miR4376 sequence expressed in the NaCl-treated leaves and roots and a group of 15 variant miRNA sequences were specifically expressed in leaves when they were exposed to salinity stress (Figure S2B, Table S4).

Classification of the conserved and miRNA variants based on sequence homology with other orthologs showed that some date palm miRNAs were similar to the miRNA sequences of 35 plant species that were available during the preparation of the manuscript in the miRbase database (Figure S3). The highest number of conserved miRNA sequences (45) was identical to *Glycine max*, followed by *Medicago truncatula* (11 miRNA sequences) and the abiotic stress–tolerating moss *Physcomitrella patens* (Frank et al., 2005) (10 miRNA sequences) (Figure S3A). Similarly, the largest number of miRNA variants (13 miRNA sequences) isolated in this study was similar to *Glycine max*, and 10 miRNA sequences were similar to those isolated from *Arabidopsis lyrata* (Figure S3B). Phylogenetic analysis of the miR156 sequences family showed that there were two miRNAs (pda-miR156e/h) are more similar to monocots specific homologs (Figure S4). However, the phylogenetic analysis of miR396/390/166 families showed that these miRNA families were well conserved among monocots and dicots (Figures S5–S7).

The deeply conserved miRNA sequences are more abundantly expressed among different plant species (Chávez Montes et al., 2014). Thus, they are easier to detect and sequence, and the conserved miRNA isolated from the date palm is expected to have a high homology level similar to those previously identified from different plant species. Sequence conservation among plant species can reflect a common evolutionary history and function for the miRNAs that are involved in common plant growth, development and stress response processes (Nozawa et al., 2012).

### Date palm encodes a large family of novel miRNAs

**Figure 4:**
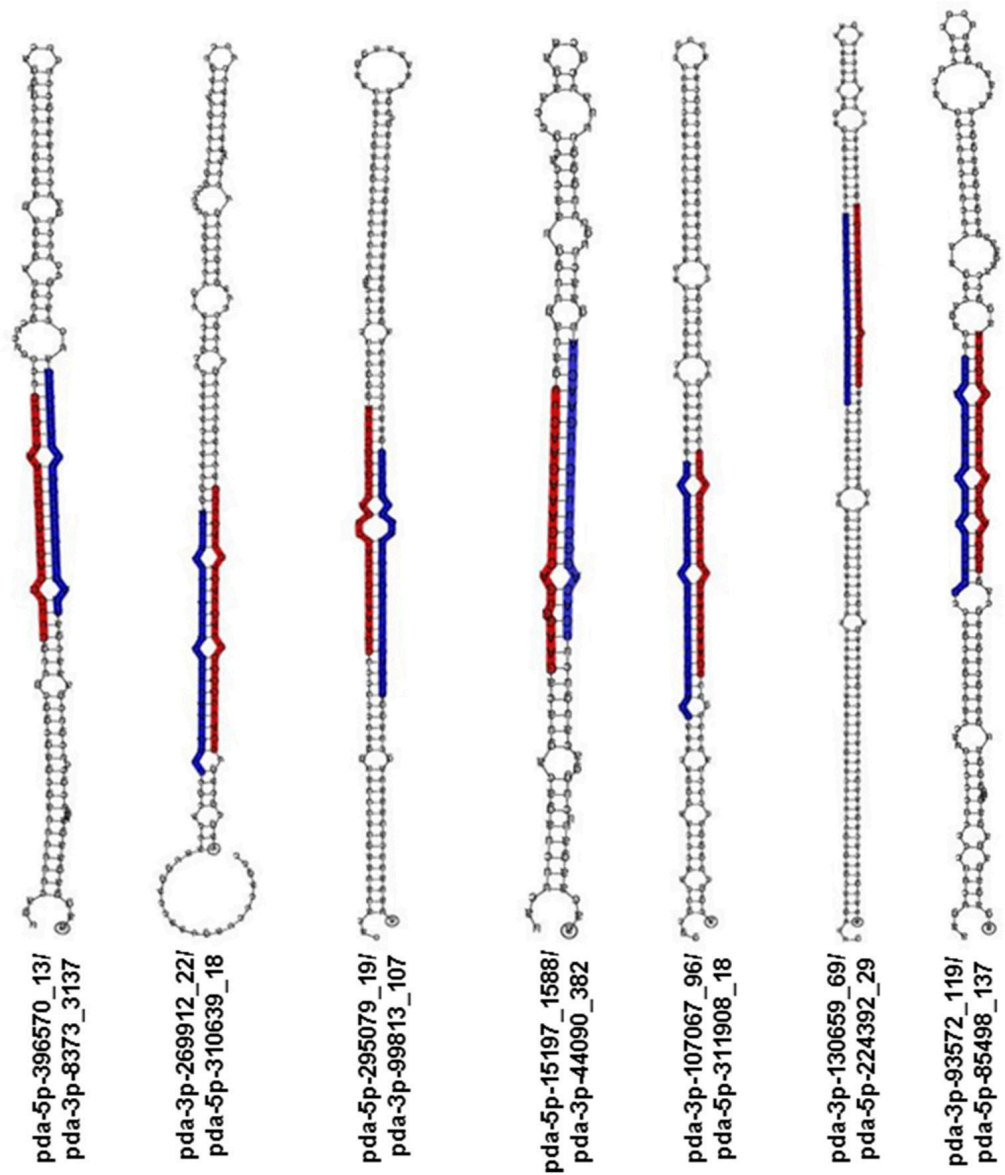
**Stem-loop RNA secondary structure of hairpin-forming precursors of some novel miRNA sequenced from ***Phoenix dactylifera*** L**. The mature 5p-miRNA/3p-miRNA sequences are shaded in either red or blue.

For novel miRNA annotation in date palm, the following criteria was used: sRNAs that have no similarity to miRNAs previously published in the miRbase and that have a minimum of 10 reads in total from the four sequenced libraries coupled with a predictable stem-loop structure for their precursors. Therefore, novel miRNA annotation is based mainly on their biogenesis from the corresponding hairpin-like precursor; thus, sRNAs with unavailable hairpin precursors were removed from further analysis because these lack a genomic origin. To ensure the appropriate location of the miRNAs on the stem, the predicted miRNA sequences were aligned on the corresponding hairpin precursor, and miRNAs with an improper location on the precursor were also ignored (Figure S1). This analysis showed 180 putative mature miRNAs sequenced from the four libraries (Table S3). The novel putative miRNA sequences included 150, 157, 109, and 117 sequences identified from the leaf-control, leaf-NaCl, root-control, and root-NaCl libraries, respectively. Of 180 putative miRNAs, the annotation of 15 novel miRNAs was based on the identification of the complimentary miRNA partner (Table 3, Figure 4), as recommended by Meyers et al. (2008). However, the absence of a complimentary sequence for some miRNAs could simply be due to its instability (Bartel, 2004; Guo and Lu, 2010) therefore, these miRNA sequences should not be overlooked as long as they are expressed in and being generated from a valid hairpin precursor. Nevertheless, the presence of an miRNA duplex (5p-miRNA/3p-miRNA) with a 2-nt overhang at the 3′ ends within the libraries, such as pda-396570_13 and pda-8373_3137; pda-269912_22 and pda-310639_18; and pda-130659_69 and pda-224392_29 (Figure 4), provides evidence that these sRNA pairs are true miRNA molecules (Figure 4, Figure S1).

The hairpin length and loop size affect the dicer processing capability (Ji, 2008). The predicted length of the identified hairpin precursors ranged from 57 to 264 nt; this range is similar to those previously identified in Arabidopsis (Zhang et al., 2006a), wheat (Yao et al., 2007), and apple (Yao et al., 2007).

The calculated average MFE negative value of the hairpin precursors of the putative predicted novel miRNA was 85.9 kcal mol^−1^ (Table S3). This MFE value was lower than the average MFE (59.5 kcal mol^−1^) of the Arabidopsis counterparts and much lower than the tRNAs (–27.5 kcal mol^−1^) or rRNAs (–33.0 kcal mol^−1^) (Bonnet et al., 2004). A cutoff value 0.85 MFEI was used to filter the miRNAs from the other RNA molecules, such as mRNA, rRNA, and tRNAs, because 90% of the miRNA precursors have an MFEI value that is greater than 0.85 (Zhang et al., 2006b). The calculated MFEI value for the predicted novel miRNA precursors of the date palm presented in this study ranges from 0.9 to 2.1, with an average value of 1.24 (Table S3). The MFEI values of the miRNA precursors were higher than the average MFEI values (0.97) of the previously identified miRNA precursors and much higher than those for tRNAs (0.64), rRNAs (0.59), and mRNAs (0.62–0.66) in seven plant species (Zhang et al., 2006b). The notion that these predicted novel miRNA sequences were generated from the corresponding hairpin precursors is supported by the fact that the pre-miRNAs have appropriate MFE and MFEI values and the miRNA was appropriately localized on the precursor stem (Figure 4, Figure S1).

The read count of the predicted miRNA and miRNA complimentary duplex sequences varied between and within the four libraries (Table 3, Tables S3, S5), which was likely because of the tissue specificity and NaCl stress treatment; however, in all of the available cases, a significant difference in the count number was observed in most 5p-miRNA/3p-miRNA pairs within the same date palm library regardless of the position of the miRNA on the arm of the precursor (5′ or 3′). In 11 out of the 20 cases, novel miRNA that originated from the 5′ strand showed a higher abundance than the 3′ partners (Table 3). The low-abundance miRNA sequence originates from the opposite arm of the same precursor. However, recent studies showed that some less abundant miRNA sequence (previously named miRNA^*^) molecules play a role in regulating their targets (Guo and Lu, 2010).

Similar to the conserved miRNAs, statistical analysis revealed the absence of a specific novel miRNA sequence that is solely expressed in the NaCl-treated roots; however, six miRNA sequences were expressed in both NaCl-treated leaf and root tissue, and two sequences were specifically expressed in the NaCl-treated leaves (Figure S2C and Table S4).

The number of novel miRNAs (180) sequenced in this study was high, although the date palm has a medium-size genome of approximately 670 Mb (Al-Dous et al., 2011), distributed among 18 chromosomes (Beal, 1937). Presumably, intensive duplication events occurred over time, which led to a large number of miRNAs, as previously shown by a computational study that was conducted on the genome (Xiao et al., 2013). It is interesting to note that 67% of the date palm miRNA members are located within duplicate fragments of the genome. Although conserved miRNAs have evolved a common role in gene regulation, variants and novel miRNAs could also be important in regulating their target gene expression (Qin et al., 2014).

### miRNAs are differentially regulated in leaf and root tissues

Similar to many other genes, the abundance of miRNAs is affected by the environmental factors including the salinity and this regulation could vary based on the type of cell- or tissue. The data obtained in the present study on differential expression in root and leaf tissue support this view. The expression profile of miRNAs in this study was quantified based on the number of reads obtained. Significantly regulated miRNAs were identified on the basis of *P* < 0.05, (FDR) < 0.05.

**Figure 5:**
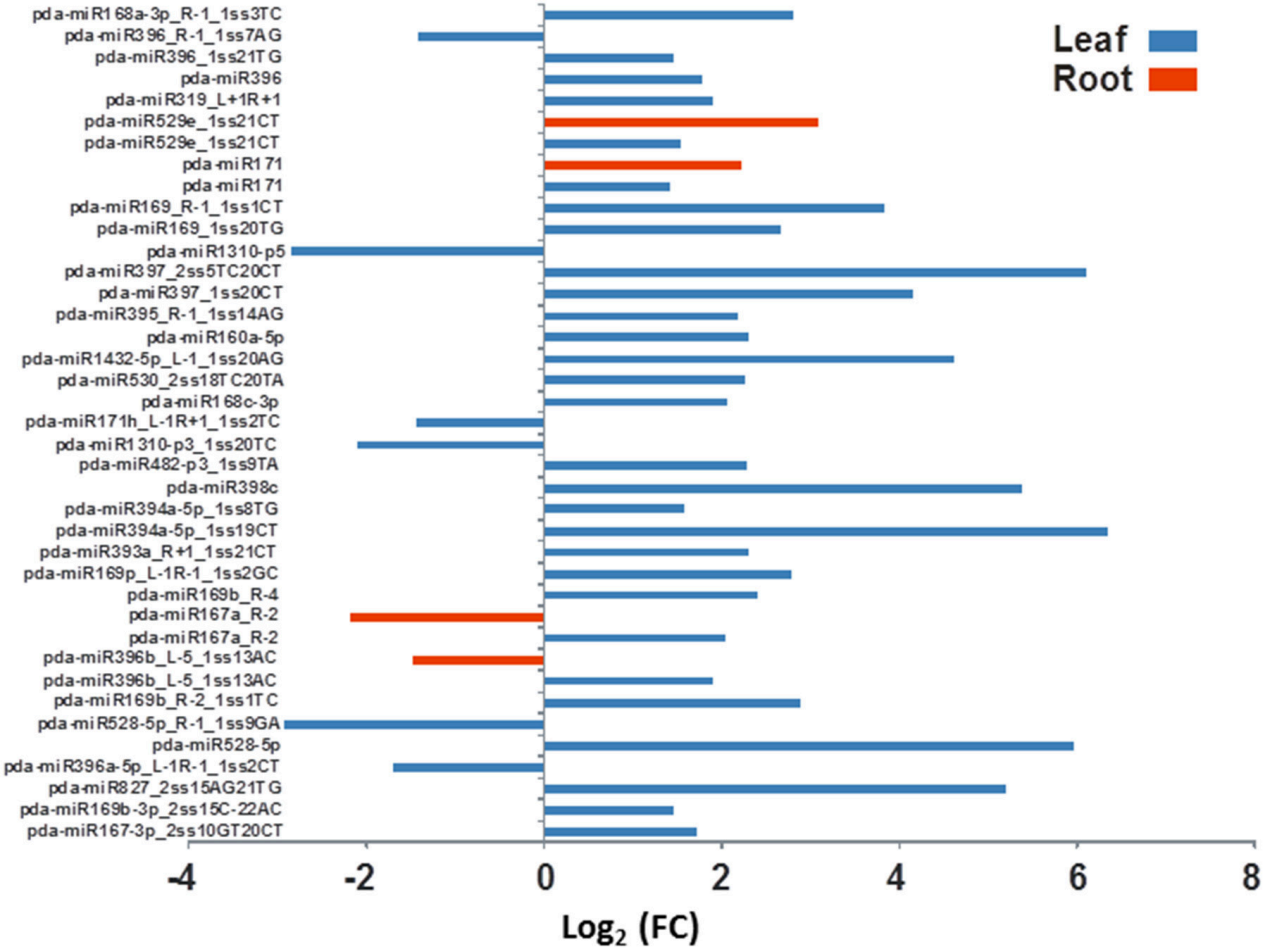
**The effect of salinity stress on the expression levels of some conserved miRNA sequenced from leaves and roots**. Fold of control expression (FC) was calculated using the normalized read counts of each miRNA. These miRNA sequences are significantly [*P* < 0.05, (FDR) < 0.05] regulated due to salinity treatment.

**Figure 6:**
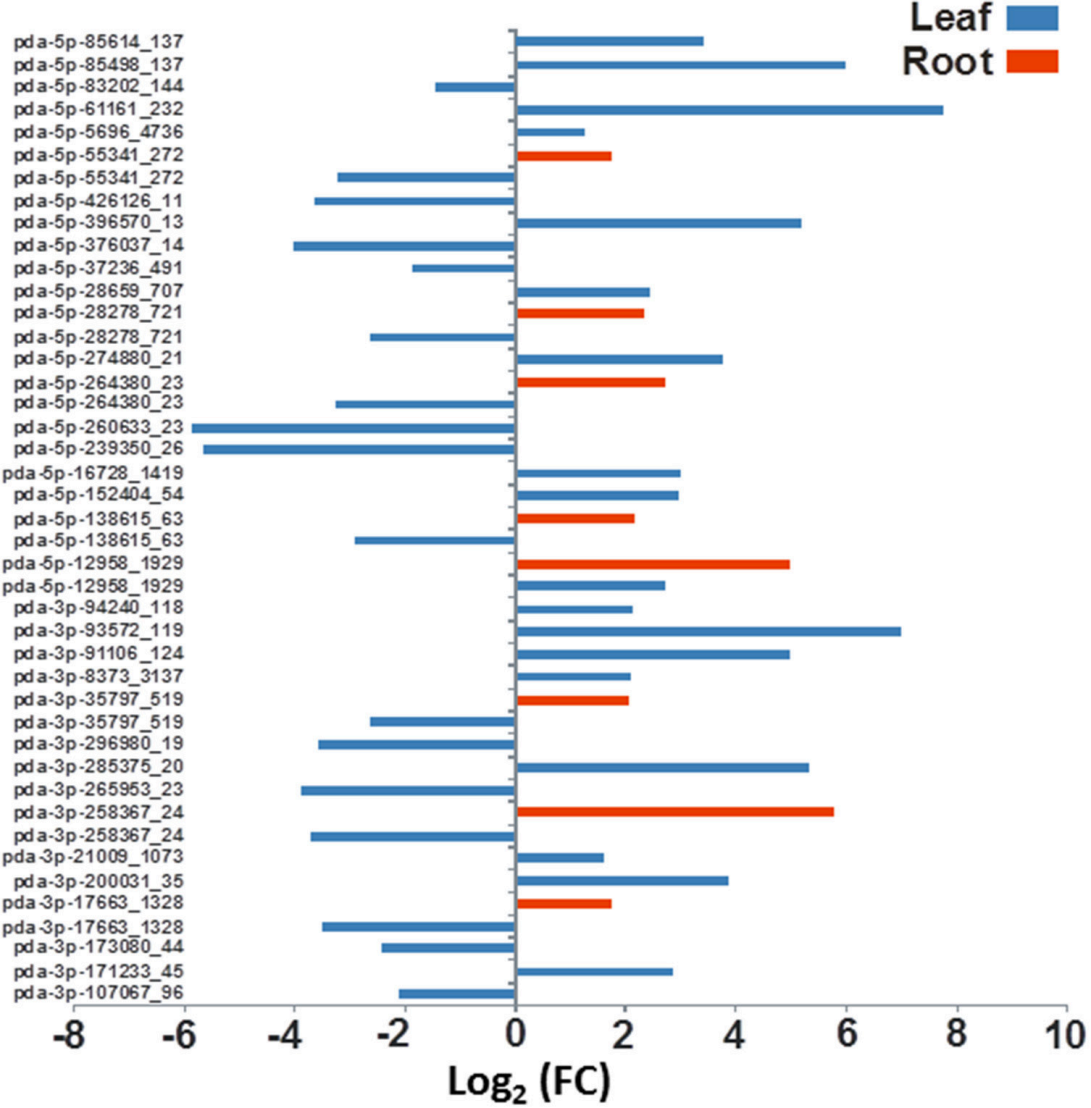
**The effect of salinity stress on the expression levels of some predicted novel miRNA sequenced from leaves and roots**. Fold of control expression (FC) was calculated using the normalized read counts of each miRNA. These miRNA sequences are significantly [*P* < 0.05, (FDR) < 0.05] regulated due to salinity treatment.

Based on the statistical analysis, 57 and 25 miRNAs are predicted as significantly regulated in leaf and root tissues under salt stress, respectively. At least 12 miRNAs were commonly regulated both in leaves and roots (Table S5). Differential expression analysis based on the normalized miRNA count values of the leaf showed that 66% of the miRNA sequences were upregulated and the rest were downregulated by salinity treatment. Similar to the leaf miRNAs, the majority of the miRNA sequenced from root (76%) were significantly induced and the rest were downregulated by the salinity treatment (Table S5, Figures 5, 6).

**Figure 7:**
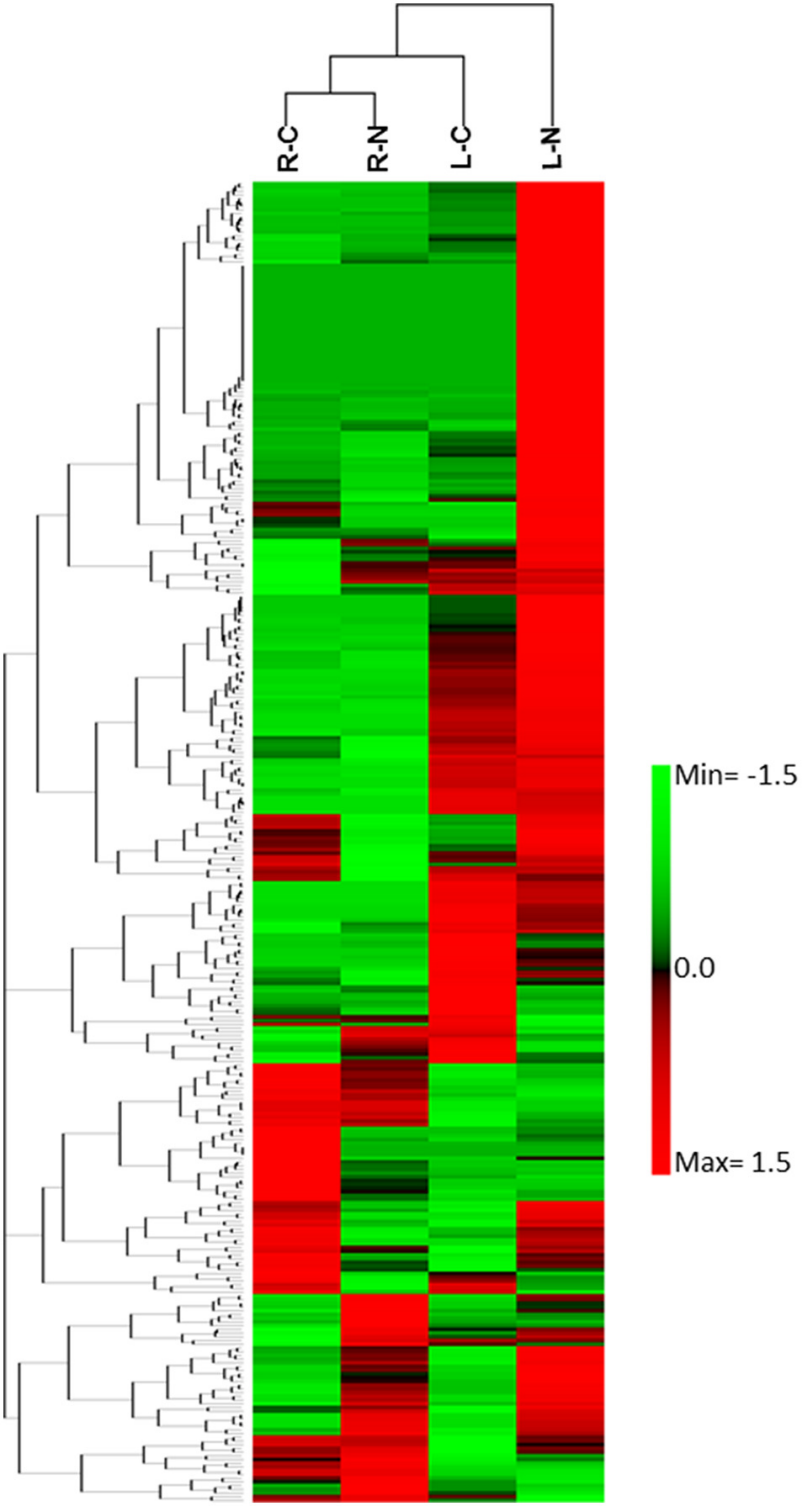
**Heat map of the hierarchical cluster analysis and a dendrogram of 355 sRNA (rows) identified in leaf growing under normal conditions (LC), leaf growing under NaCl stress, root growing under normal conditions (RC), and roots growing under NaCl stress conditions (RN)**.

The differential gene expression due to the salinity treatment in the leaves and roots was observed for conserved miRNAs (Figure 5) as well as for potential novel miRNAs (Figure 6). Hierarchical cluster analysis of the normalized miRNA count values of 355 miRNAs identified in leaves and roots grown in normal and salt stress conditions revealed a differential expression pattern among the four libraries. In general, the analysis showed clear upregulation of miRNAs expressed in leaves when they were exposed to salinity stress however, libraries prepared from roots showed a mixture of high and low count values due to salinity treatment. Although the two libraries that were prepared from roots shared a conserved expression profile regardless of the salinity treatment, the expression profiles of the two leaf libraries were highly divergent (Figure 7).

The miRNAs profiles showed an inconsistent pattern of gene expression due to salinity in different plant species. For example, in Arabidopsis a group of miRNA including miR156, miR158, miR159, miR165, miR167, miR168, miR169, miR171, miR319, miR393, miR394, miR396, and miR397 were induced, while the miR398 was inhibited due to salinity stress (Liu et al., 2008). The hierarchical clustering analysis of a group of miRNA sequences identified from the roots of the salt-sensitive maize line Huangzao4 revealed a moderate inhibition in miRNA expression due to 200 mM NaCl treatment for 24 h (Ding et al., 2009). Similarly, 11 miRNAs identified from *Solanum linnaeanum* roots were downregulated, and only three were induced due to salt stress (Zhuang et al., 2014). In *Thellungiella salsuginea* roots, six miRNAs (miR156a, miR156k, miR162a, miR164a, miR171c, and miR824) were downregulated and only four miRNAs (miR160a, miR168a, miR395a, miR395b) were upregulated. However, the same treatment in leaves induced five miRNAs (miR160a, miR164a, miR168a, miR359a, and miR359b), repressed four miRNAs (miR156k, miRA162a, miR171c and miR824), and stabilized the expression of one miRNA (miR156a) (Zhang et al., 2013). Salinity stress was shown to downregulate global miRNA expression patterns in *Brassica juncea* (Bhardwaj et al., 2014). These observations suggest downregulation of miRNAs during salinity is commonly observed in different plant species.

Most of the available reports on deferential miRNA expression studies focused on investigating miRNA abundance in whole plants. Some of the reports showed a fluctuation in miRNA expression levels in roots and leaves when they were exposed to abiotic stresses. For example, in barley, dehydration induces miR166 in leaves but represses the same in roots; and induces miR156a, miR171, and miR408 in leaves but does not change their expression in roots (Kantar et al., 2010). A similar expression variation pattern was found in *Medicago truncatula* that was exposed to water stress; miR398 was induced in leaves relatively more than in roots (Trindade et al., 2010).

**Figure 8:**
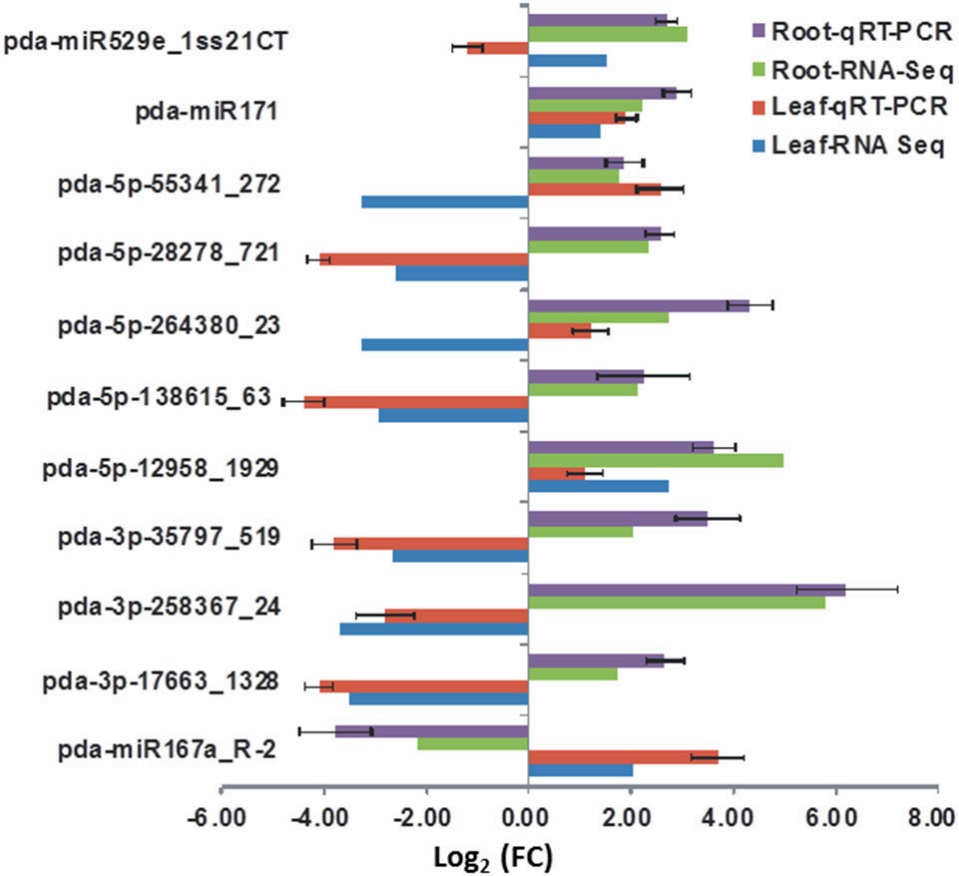
**The expression levels of selected miRNA detected by RT-qPCR and their corresponding normalized read counts (RNS-Seq) which were significantly (***P*** < 0.05, (FDR) < 0.05) regulated due salinity treatment**. The expression value was calculated as the fold of control (FC) of each expression category. The RT-qPCR value represents an average of three biological samples ± S.E.

The validity of read count of known and novel miRNAs observed in sequencing platform was verified using RT-qPCR. miRNAs expressed in both leaves and roots with a significant (*P* < 0.05, FDR < 0.05) change in the read numbers in response to salinity were selected for RT-qPCR analysis. The RT-qPCR analysis revealed that the expression level is not necessarily consistent as compared to the normalized count numbers obtained using the high-throughput sequencing approach (Figure 8). Similar inconsistencies between the two approaches were found in alfalfa (Long et al., 2014) and artichoke (De Paola et al., 2012). Regardless of this inconsistency, the RT-qPCR results confirmed the presence and expression of the tested miRNAs in date palm tissues. Optimizing and verifying the expression level of every miRNA identified in this study are beyond the scope of the present study.

### miRNAs target the potential key genes that are involved in the salt adaptation mechanism

The date palm genes were recently annotated (Al-Dous et al., 2011) but not experimentally characterized. A functional miRNA can regulate a single gene or multiple genes of the same or different families (An et al., 2010). To obtain insights into the potential functions of miRNAs, the target genes for the identified conserved miRNAs and miRNA variants, and novel miRNAs were predicted using a small plant RNA target analysis server, which was pre-uploaded with the date palm cDNA sequences. The results showed that 94.3% of the characterized miRNAs in the leaves and roots have potential targets in the date palm genome. Of the 372 miRNAs expressed in the leaves, 351 were predicted to target 598 genes, and of the 298 miRNAs expressed in the roots, 284 were predicted to target 473 genes (Table S6).

miRNAs can inhibit the protein production of the target gene by base pairing with the perfectly complementary mRNA sequence or even when the base pairing is imperfect and has mismatches around the center of the complementary region on the target mRNA. In the latter case, the miRNAs inhibit the translation of the mRNA on the ribosomes (Brodersen et al., 2008). In date palm leaves, out of 898 predicted targets, 660 (73.5%) possess potential sites for miRNA-guided cleavage, and 239 targets possess sites for potential translational inhibition. Similarly, of 721 predicted target genes for miRNAs in the roots, 531 have sites for potential cleavages and 190 have sites for translational blockage. The miRNA sequences that were identified from plant species exhibit a high degree of sequence complementarity with their target mRNA. Our findings are in agreement with the previously assumed notion that mRNA cleavage is the principal mechanism of mRNA deactivation in plants (Jones-Rhoades et al., 2006), unlike animal miRNAs, most plant miRNAs can be predicted to target a few genes through perfect base pairing, which leads to cleavage of the target mRNA (Voinnet, 2009).

The miRNA target analysis also showed that an miRNA sequence could have one or more mRNA targets. For example, the pda-miR156i-3p_R-3_1ss6AT and pda-3p-44090_382 miRNA sequences each targeted 16 mRNA sequences in the leaf and root libraries. In addition, some mRNA sequences were targeted by different miRNAs. For example, PDK_30s756061g001 was targeted by 13 miRNAs in the leaf and root tissues (Table S6).

**Figure 9:**
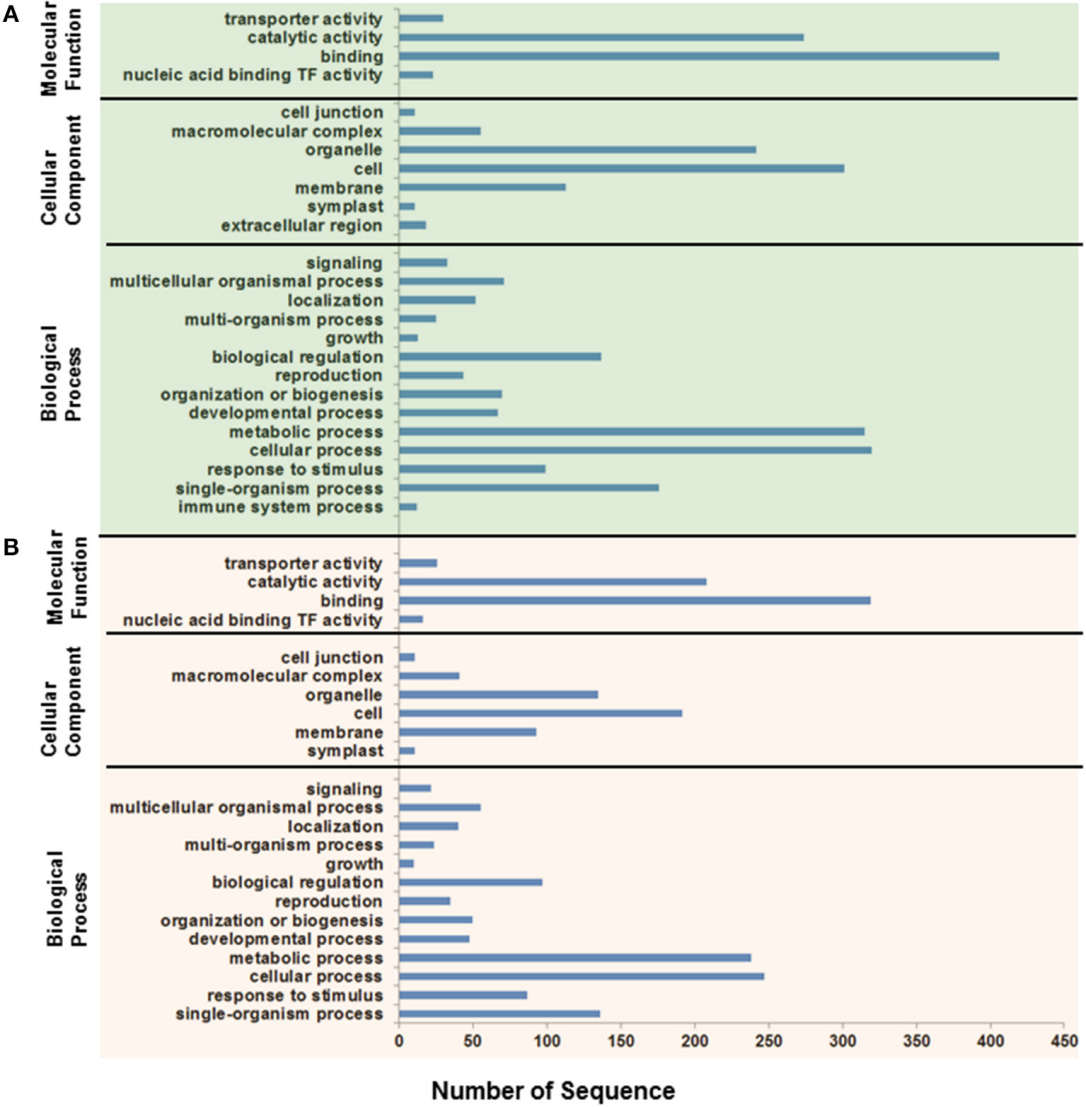
**Functional classification of ***Phoenix dactylifera*** L. miRNA putative targets expressed in leaves (A) and roots (B) based on Gene Ontology (GO) annotation**. The gene targets were classified based on the molecular function, cellular component, and biological processes.

To obtain an overview of the biological functions, the predicted targets were subjected to gene annotation and ontology (GO) analysis at the molecular function, cellular component, and biological process levels. The GO analysis showed that the largest subset of the predicted targets displayed binding molecular function, which was localized in the cellular component and involved in metabolic and cellular processes (Figure 9). Of particular interest in salinity adaptation is that a significant number of the targets (26) of the root miRNAs were predictably involved in transporter functions, with 93 targets localized in membranes, and 87 were involved in response processes to stimulus (Figure 9B). A similar GO distribution was also found in the leaves for miRNA targeted sequences (Figure 9A). A closer look at the list of target genes revealed the presence of some gene orthologs, which could be involved in the adaptation to salinity in the date palm (Table 4, Table S6). A short list includes genes that encode for phytohormone-related proteins, such as ABA responsive elements-binding factor, Cytokinin dehydrogenase 3, and IAA-amino acid hydrolase ilr1-like 6-like. Among the targets are a group of genes that encode transcription factors, such as C-myb, ethylene-responsive transcription factor rap2, floral homeotic protein APETALA2-like, NAM (no apical meristem), Arabidopsis transcription activation factor, and cup-shaped cotyledon (NAC), and genes that code for calcium-dependent and mitogen-activated protein kinases and ion sorting and transport, such as potassium channel AKT2-like and vacuolar protein sorting-associated protein (Table 4, Table S6). Interestingly, predicted novel miRNAs such as pda-3p-130659_69, pda-3p-44090_382, pda-3p-313824_18, and pda-5p-5696_4736 were among those miRNAs that were significant in salinity tolerance. Previous studies that were conducted on other plant species showed that these target gene orthologs are potentially important for salt stress tolerance (Roy et al., 2014). Although these computationally generated data are important and could provide a basis for identifying the salt adaptation mechanisms in the date palm, this information should be taken with some caution until it has been validated/confirmed.

**Table 4:** **Putative targets of selected miRNAs**.

**Class**	**miRNA_Acc**.	**Target Acc. Number**	**Inhibition**	**Target description**
H.O.R.	pda-miR529e	PDK_30s774191g002	Cleavage	Abscisic acid responsive elements-binding factor
	pda-miR160b, pda-miR160a, pda-miR160a	PDK_30s951701g002	Cleavage	Auxin response factor 17-like
	pda-miR156g, pda-miR156c	PDK_30s928741g002	Cleavage	Cytokinin dehydrogenase 3-like
	pda-3p-130659_69	PDK_30s65509272g006	Cleavage	Gibberellin 2-beta-dioxygenase 8-like
	pda-3p-44090_382	PDK_30s1006281g001	Cleavage	IAA-amino acid hydrolase ilr1-like 6-like
K.I.N.	pda-miR166a	PDK_30s6550978g002	Cleavage	Calcium-dependent protein kinase 28-like
	pda-miR397	PDK_30s903491g006	Cleavage	Mitogen-activated protein kinase
T.F	pda-3p-313824_18	PDK_30s770411g002	Cleavage	C-myb-like transcription factor myb3r-4
	pda-miR396	PDK_30s1150791g004	Translation	Ethylene-responsive transcription factor rap2-2-like
	pda-miR167a, pda-miR167c	PDK_30s1201081g001	Cleavage	F-box protein skip16-like
	pda-miR172a, pda-miR172i, pda-miR172c	PDK_30s749921g002	Cleavage	Floral homeotic protein apetala 2-like
	pda-miR164, pda-miR164, pda-miR164a	PDK_30s1198591g001	Cleavage	Nac domain-containing protein 21 22-like
	pda-miR156a, pda-miR156c, pda-miR156h, pda-miR156e, pda-miR156g, pda-miR529e, pda-miR156b	PDK_30s756061g001	Cleavage	Promoter-binding protein spl9
	pda-miR156h	PDK_30s1139181g002	Translation	Squamosa promoter-binding-like protein 16
T.S.P.	pda-miR828a	PDK_30s927361g003	Cleavage	Abc transporter f family member 3
	pda-miR393a	PDK_30s947241g003	Cleavage	Transport inhibitor response 1-like protein
	pda-miR535	PDK_30s1187081g002	Cleavage	potassium channel akt2-like
	pda-5p-5696_4736	PDK_30s693381g001	Cleavage	Vacuolar protein sorting-associated protein 41 homolog

*The genes were classified as hormones (H.O.R.), Kinases (K.I.N.), Transcription factors (T.F.), or transportation (T.S.P.)-related function. The miRNAs were selected based on their targets, which could have a functional relationship to salinity tolerance mechanisms in plants*.

At present, validating the expression level of a certain miRNA and correlations with the expression level of the potential target gene in date palm is not an easy approach because unlike other plant species, such as Arabidopsis or rice, the date palm genome has been partially sequenced or assembled and the predicted mRNAs for some genes are still not well-defined. The available date palm genes in the database, including those with potential function in salinity tolerance, are not functionally characterized but are completely annotated based on sequence homology with other orthologous genes of known function (Al-Dous et al., 2011). In addition, unlike the conserved miRNA sequences (Zhang et al., 2006a), these mRNA sequences may have several known or unknown polymorphic alleles which make the RT-qPCR unreliable at this stage. Furthermore, the available date palm genome belong to *Khalas* cultivar which may not completely match with the cultivar we are using in this study given that the nomenclature of date palm cultivars is not uniformly standardized worldwide (Yaish and Kumar, 2015). This can be addressed by sequencing the mRNA of the same date palm cultivar growing under the same conditions.

To obtain more detailed information on the respective biochemical functionalities, the predicted target genes were mapped to metabolic pathways using the KEGG tool. The results showed that 123 and 85 target genes that coded for 41 and 30 enzymes were mapped on 64 and 55 known pathways in the leaves and roots, respectively (Tables S7, S8). The list also included pathways such as purine metabolism, glycolysis/gluconeogenesis; fructose, mannose metabolism; starch and sucrose metabolism, which have been previously characterized as important for salt tolerance mechanisms in plants.

Intriguingly, eight of the predicted target enzymes in leaves were mapped on the starch and sucrose metabolic pathways (Figure S8). It was previously reported that salinity stress affects the global carbon metabolism and the starch/sucrose ratio in plants (Vinocur and Altman, 2005). According to Wang et al. (2013), the majority of differentially accumulated proteins in *Thellungiella halophila* leaves are associated with carbohydrate metabolism. Date palms can adapt to high salinity levels and can even grow on the shoreline and be exposed to seawater (unpublished data); however, in this situation, the plants are unable to produce edible fruit probably because the carbohydrate metabolism is impaired and possibly used most of the photosynthates for salt tolerance instead of storing the energy in the fruits. KEGG mapping tools showed eight other genes that encoded for five enzymes mapped on the purine synthesis pathway in leaves and roots (Figure S9). Previous studies also showed that a group of purine-related proteins are differentially accumulated in salt-treated rice seedlings (Yan et al., 2006), and accumulation of this group of proteins is important for salt tolerance in rice and other plant species (Stasolla et al., 2003).

In conclusion, our results revealed the identification of miRNAs that share common biogenesis, structure, and expression features with the miRNAs that have been isolated from other plant species. These miRNAs and their targets could play a critical role in the date palm tree, which has the ability to grow under severe environmental conditions, including high temperature, drought, and salinity. These abiotic stress tolerance activities could require a complex coordination of physiological processes, including miRNA-dependent gene regulation. The miRNAome information that was revealed by this study is an important component of gene regulation circuitry and could provide more insight in understanding the salinity tolerance in date palm.

## Author contributions

MY designed, performed the experiments, analyzed data and wrote the paper. RS analyzed data and wrote the paper. YZ and BJ analyzed data. RA wrote the paper. SF wrote the paper.

### Conflict of interest statement

The authors declare that the research was conducted in the absence of any commercial or financial relationships that could be construed as a potential conflict of interest.
