# Supplementary material for: A genome-wide identification of the miRNAome in response to salinity stress in date palm (Phoenix dactylifera L.)
Source: Front Plant Sci. 2015 Nov 5;6:946. doi: 10.3389/fpls.2015.00946 (PMC4633500; doi:10.3389/fpls.2015.00946)
Supplement: Supplementary file 4 [file Table4.DOCX]

**Table S4.** Tissue-specific miRNA produced by leaves and roots under normal and saline conditions.

| **miRNA family** | **Sequence** | **Locus** | **Length (nt)** | **MFE** | **mi** | **Reference miRNA** |
| --- | --- | --- | --- | --- | --- | --- |
| **Conserved Common miRNA in "Leaf-NaCl" and "Root-NaCl"** | |  |  |  |  |  |
| pda-miR169a | UAGCCAAGGAUGACUUGCCA | PDK_30s729731 | 20 | -74.4 | 1 | cca-miR169a_R-1_1ss20TA |
| pda-miR169a | UAGCCAAGGAUGACUUGCU | PDK_30s729731 | 19 | -73.6 | 0.8 | cca-miR169a_R-2_1ss19CT |
| pda-miR398a | UGUGUUCUCAGGUCGCCCCUUU | PDK_30s874111 | 22 | -48.9 | 0.9 | gma-miR398a_R+1_1ss15AG |
| **Conserved miRNA family expressed only in Leaf-NaCl** | |  |  |  |  |  |
| pda-miR169j | GGGCAGUCUCCUUGGCUAGUC | PDK_30s1124371 | 21 | -80.1 | 1 | aly-miR169j-3p_L+1R+1_2ss19TG20CT |
| pda-miR396e | GGUCAAGAAAGCUGUGGAAG | PDK_30s756541 | 20 | -56.4 | 0.9 | zma-miR396e-3p_R-1_3ss13CT18GA20AG |
| pda-miR172b | UGGCAUCAUCAAGAUUCACAU | PDK_30s955041 | 21 | -61.0 | 1.2 | gma-miR172b-5p_L-1R+2_1ss3AG |
| pda-miR164 | UUGCACGUGCCCUGCUUCUCC | PDK_30s747091 | 21 | -64.9 | 1 | vun-miR164-p3 |
| pda-miR394a | AGGUGGGCAUCCUGCCAACUG | PDK_30s918941 | 21 | -83.9 | 1.1 | zma-miR394a-3p_R+1_3ss11AC19TC20GT |
| pda-miR160a | GCGUGCAAGGAGCCAAGCAU | PDK_30s65509285 | 20 | -48.7 | 1.1 | osa-miR160a-3p_R-1 |
| pda-miR160a | ACAGCUUUCUUGAACCG | PDK_30s999911 | 17 | -57.3 | 1.1 | osa-miR160a-3p_R-1 |
| pda-miR399d | UGCCAAAGGAGAUUUGCCCAG | PDK_30s868971 | 21 | -64.4 | 0.8 | gma-miR399d |
| pda-miR169e | GGCAGUCUCCUUGGCUAGUA | PDK_30s1187501 | 20 | -78.4 | 0.9 | bdi-miR169e-3p_R+1_1ss19CT |
| pda-miR395g | UUGAAGUGUUUGGGGGAACU | PDK_30s849361 | 20 | -51.3 | 0.7 | mtr-miR395g_R-1 |
| pda-miR5632 | UUGGAUUUAUAGUUGGAU | PDK_30s987991 | 18 | -19.8 | 0.8 | ath-miR5632_R-3 |
| pda-miR395a | GUUCCCUCAGACACUUC | PDK_30s6550926 | 17 | -80.6 | 0.8 | ppe-miR395a-5p_R-3_1ss10AG |
| pda-miR169 | CAGCCAAGGAUGAUUUGC | PDK_30s891391 | 18 | -57.1 | 1 | pde-miR169_R-3_1ss14CT |
| pda-miR394a | AGCUCUGUUGGCUUCUCUUUG | PDK_30s801231 | 21 | -67.7 | 0.9 | mdm-miR394a-p3 |
| pda-miR529 | GCUGUACCCUCUCUCUUCUUC | PDK_30s912631 | 21 | -58.2 | 1.1 | bdi-miR529-3p |
| **Variant-Common elements in "Leaf-NaCl" and "Root-NaCl":** | |  |  |  |  |  |
| pda-miR164 | CACGTGCCCTACCTCTCC | PDK_30s946341 | 18 | -58.9 | 0.8 | cca-miR164-p3_1ss8TC |
| pda-miR4376 | TTCGCAGGAGAGATGATGCT | PDK_30s1122511 | 20 | -59.9 | 1.3 | sly-miR4376-p5_1ss2AT |
| **Variant-conserved Elements only in "Leaf-NaCl":** | |  |  |  |  |  |
| pda-miR1424 | CUGAACAACAAGAUCAUA | M01000043814 | 18 | -18.1 | 0.8 | osa-miR1424-p3_1ss15TC |
| pda-miR160e | GCGUGCGAGGAGCCAAGCAUA | PDK_30s969081 | 21 | -75.4 | 0.9 | osa-miR160e-3p_2ss11TA21GA |
| pda-miR171j | UGUUGGCCCGGUUCACUCAGA | PDK_30s886631 | 21 | -53.0 | 0.9 | gma-miR171j-5p_2ss2AG9TC |
| pda-miR172a | UUUGCUGGUGUGGCAUCAUC | PDK_30s661361 | 20 | -57.0 | 1.2 | aly-miR172a-p5_1ss8CG |
| pda-miR167d | CAUACAUGUGUGUAUAUAU | PDK_30s874571 | 19 | -35.4 | 1.5 | aly-miR167d-p3_1ss4GA |
| pda-miR169d | UAGCCAAGAAUGAAUUGCCU | PDK_30s729731 | 20 | -72.4 | 0.9 | bdi-miR169d-p5_1ss14CA |
| pda-miR4241 | UCAGUCGAUGUAAGAAAU | M01000042451 | 18 | -61.4 | 1.2 | aly-miR4241-p3_1ss4TG |
| pda-miR530a | UUUCUUUCUUUGGUUUUUG | PDK_30s726751 | 19 | -17.9 | 0.7 | ptc-miR530a-p3_1ss12TG |
| pda-miR535 | UGACAACGAGAGGGAGCACGC | PDK_30s939421 | 21 | -57.7 | 0.8 | pab-miR535_1ss13AG |
| pda-miR2111b | CAGGAUUGGGUAAUUUGC | M01000000011 | 18 | -41.2 | 0.5 | ptc-miR2111b-p3_1ss15CT |
| pda-miR166j- | UUCGGGAGAUGGAUU | PDK_30s889281 | 15 | -58.2 | 0.7 | lus-miR166j-p5_1ss13GA |
| pda-miR535 | GUGCUCUUUCUCGUUGUCA | PDK_30s715261 | 19 | -74.8 | 0.9 | tcc-miR535-p3_1ss8CT |
| pda-miR172b | GUGGCAUCAUCAAGAUUCAC | PDK_30s1019081 | 20 | -48.1 | 1 | gma-miR172b-5p_1ss3AG |
| pda-miR397 | CCAGCGCUGCAUUCGA | PDK_30s816511 | 16 | -54.6 | 0.9 | rco-miR397-p3_1ss15AG |
| pda-miR160b | UGCCUGGCUCCCUGAAUGCCA | PDK_30s969081 | 21 | -75.4 | 0.9 | ppt-miR160b_2ss1CT15TA |
| **Predicted Novel** |  |  |  |  |  |  |
| **Predicted Novel Elements only in "Leaf-NaCl":** | |  |  |  |  |  |
| pda-3p-349991_15 | UAAAUGCUGCUGUUGAUUCAA | PDK_30s684081 | 21 | -90.1 | 1.4 |  |
| pda-5p-255507_24 | CACCGUUGGAUCAUCCACAGG | PDK_30s682841 | 21 | -90.7 | 1 |  |
| **Predicted Novel Common elements in "Leaf-NaCl" and "Root-NaCl":** | |  |  |  |  |  |
| pda-5p-283724_20-R1 | UCUGGAGACCCAUAGCCCAUG | PDK_30s692211 | 21 | -71.0 | 2 |  |
| pda-5p-448267_11 | AGAUCUUAGGUCGGCACAGUAGA | PDK_30s869491 | 23 | -118.6 | 1.8 |  |
| pda-5p-420745_12 | AGGAUUGUCGAGUCGACUCGA | PDK_30s809831 | 21 | -48.4 | 0.9 |  |
| pda-5p-353741_15 | AUUGAGAUGUUUGGGGACCUGGUG | PDK_30s1146771 | 24 | -46.3 | 0.9 |  |
| pda-5p-396570_13 | AGAGUCGUGCGGUCUACAGAG | PDK_30s742991 | 21 | -80.3 | 1 |  |
| pda-3p-285375_20 | AUUAGGACUCUAUUUGAAUC | PDK_30s6550957 | 20 | -47.9 | 1.2 |  |
