# Supplementary material for: A genome-wide identification of the miRNAome in response to salinity stress in date palm (Phoenix dactylifera L.)
Source: Front Plant Sci. 2015 Nov 5;6:946. doi: 10.3389/fpls.2015.00946 (PMC4633500; doi:10.3389/fpls.2015.00946)
Supplement: Supplementary file 9 [file Image1.PDF]

Figure S1. Stem-loop RNA secondary structure of hairpin forming precursors of the 180 novel miRNA sequenced from *Phoenix dactylifera* L. and characterized in this study. The mature 5p-miRNA/3p-miRNA sequences are shaded either in red or blue color.

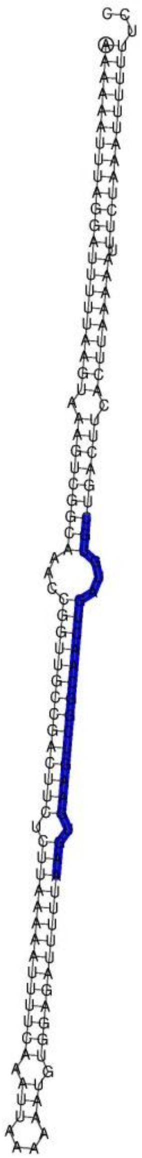

pda-3p-105149_99

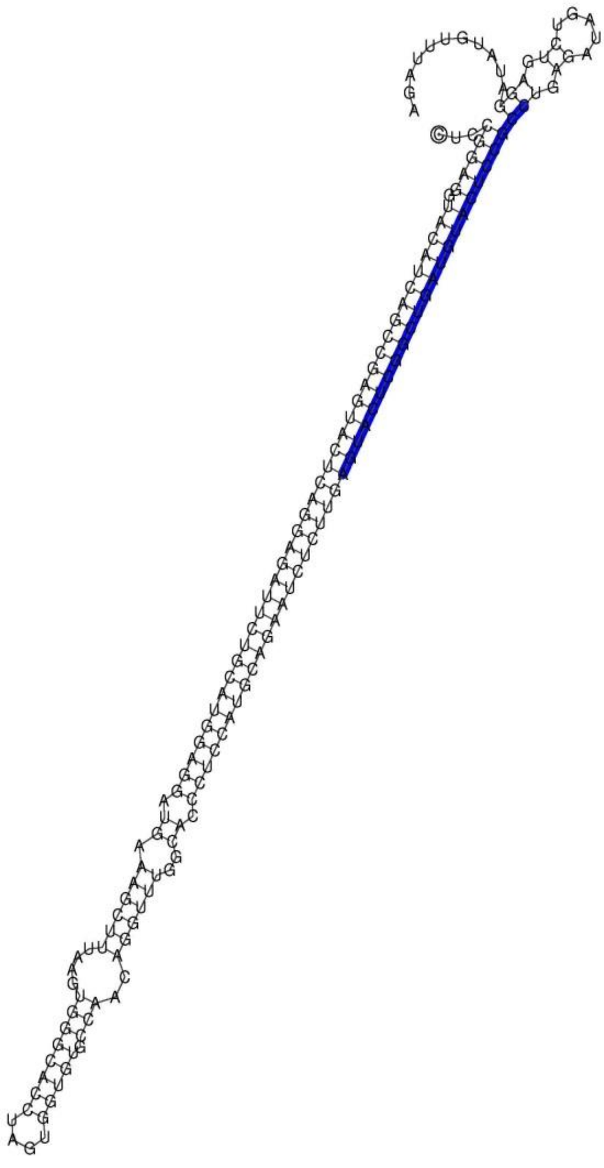

pda-3p-132860_68

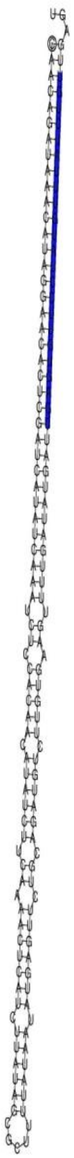

pda-3P-241095_26

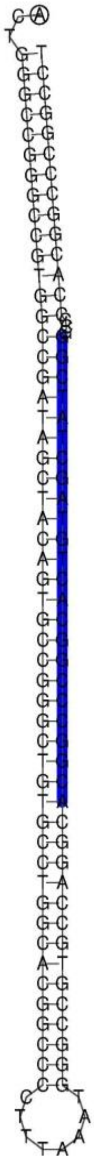

pda-3p-258367_24

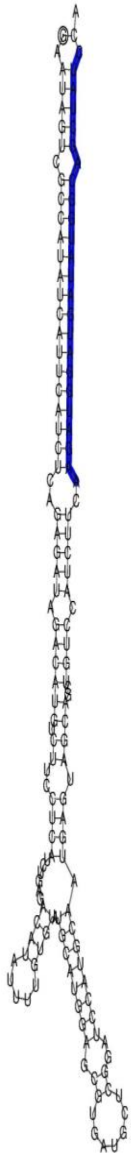

pda-3P-258784_23

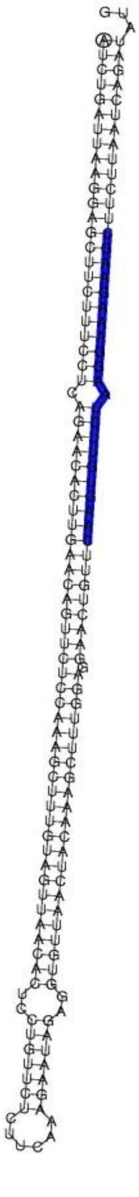

pda-3P-274391_21

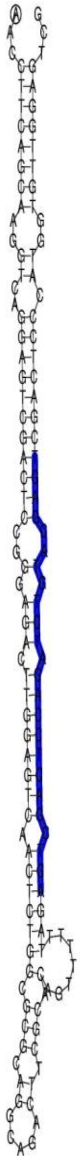

pda-3p-296860_19

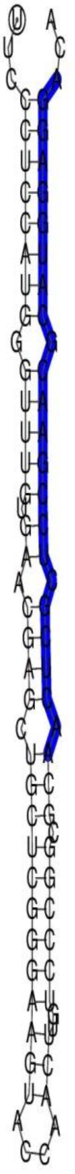

pda-3P-302733_19

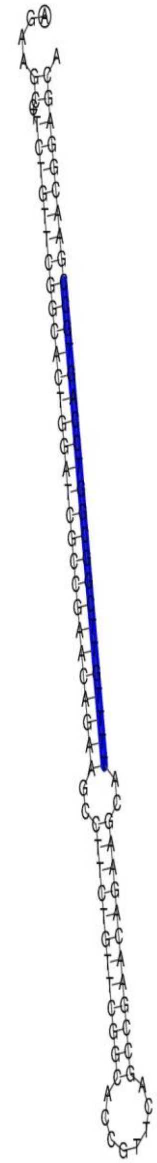

pda-3p-313542_18

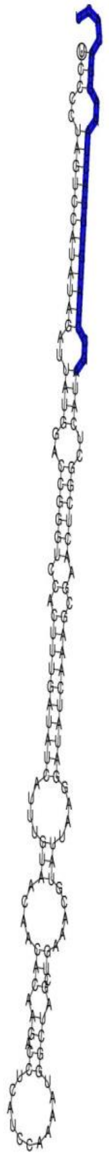

pda-3P-386334_13

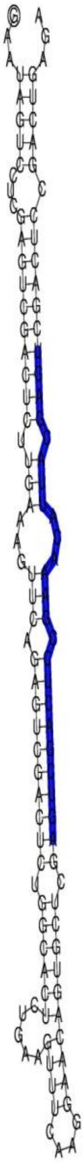

pda-3P-472966_10

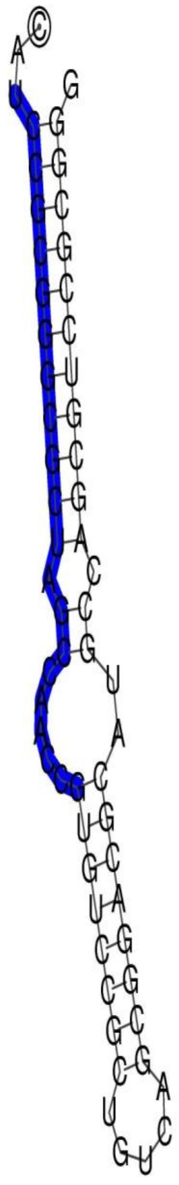

pda-5P-115656_85

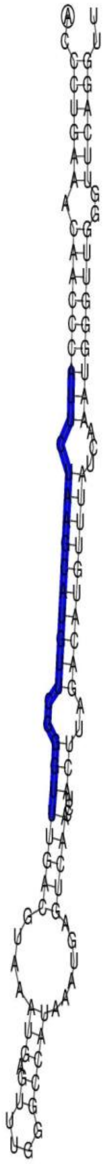

pda-5P-172763_44

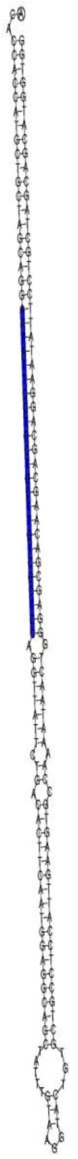

pda-5p-214434_31

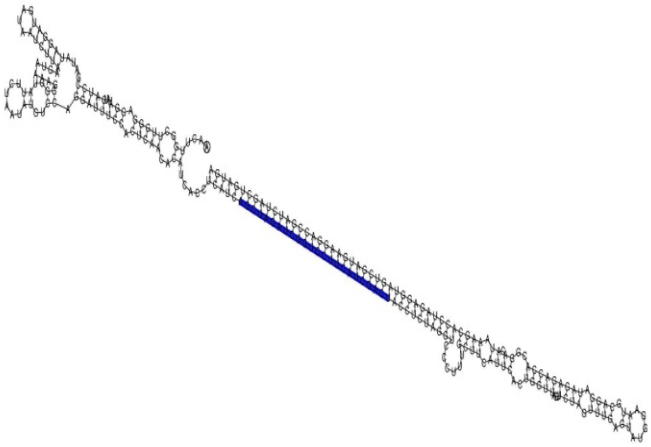

pda-5P-271611_22

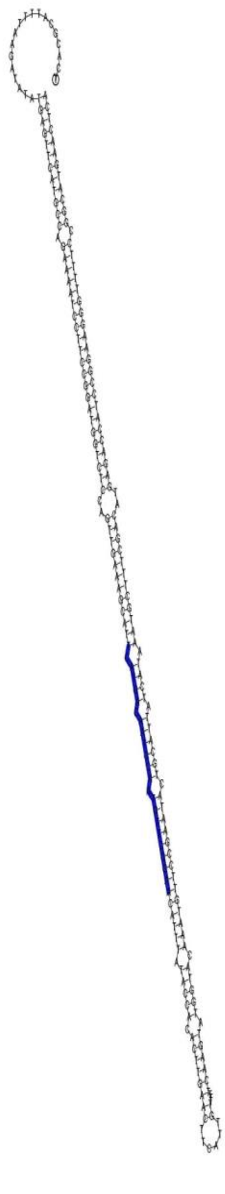

pda-5p-350550_15

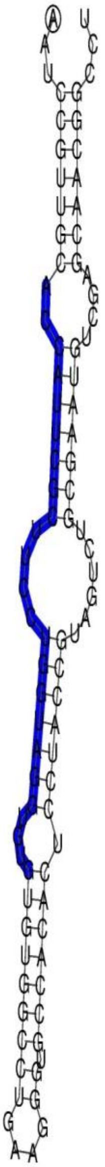

pda-5P-370156_14

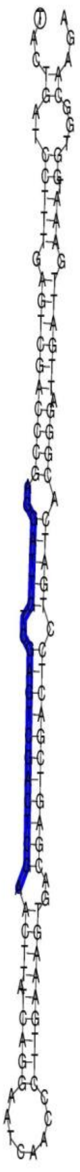

pda-5p-420745_12

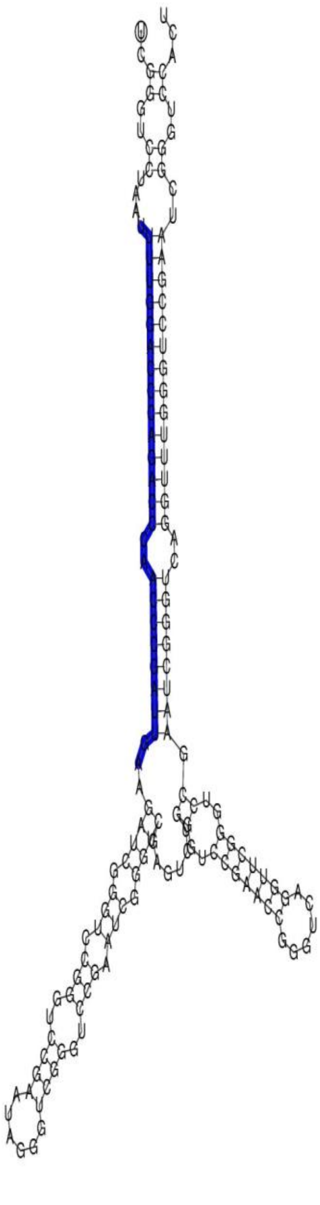

pda-5P-426126_11

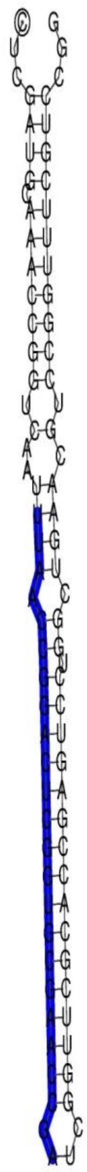

pda-5P-438543_11

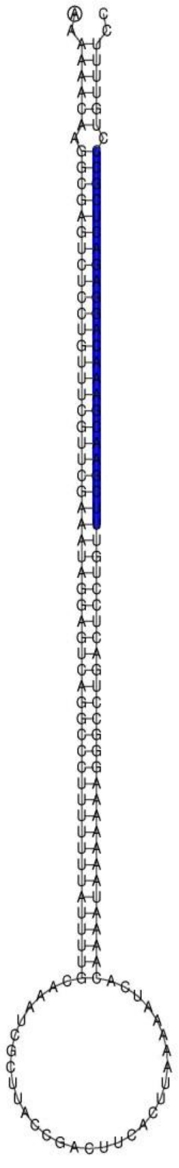

pda-3p-217326_31-R4

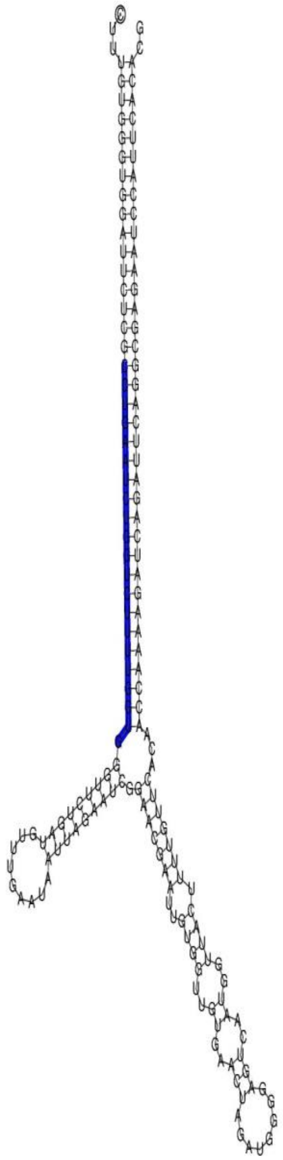

pda-5p-217347_31-R5

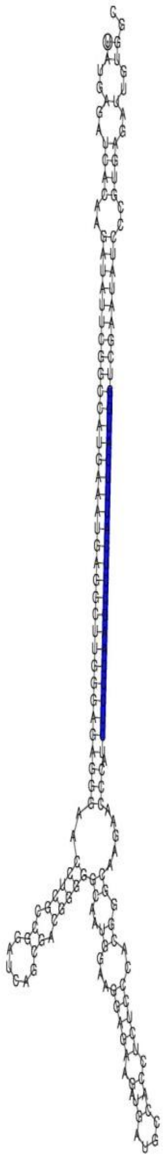

pda-3p-165824_47-R18

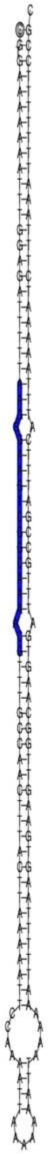

pda-5p-48683_330

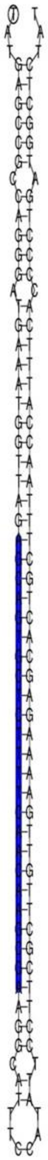

pda-5p-231728_28

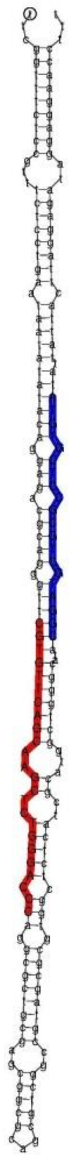

pda-3p-117918_82/pda-5p-92754_121

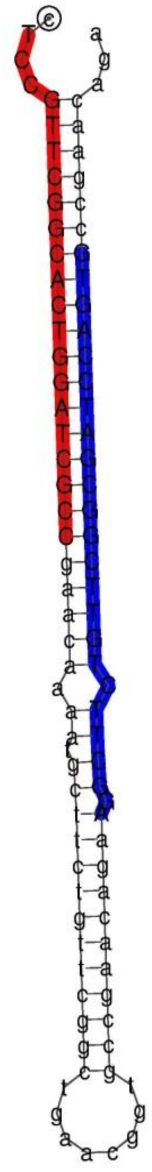

pda-3p-224491_29/pda-5p-58294_251

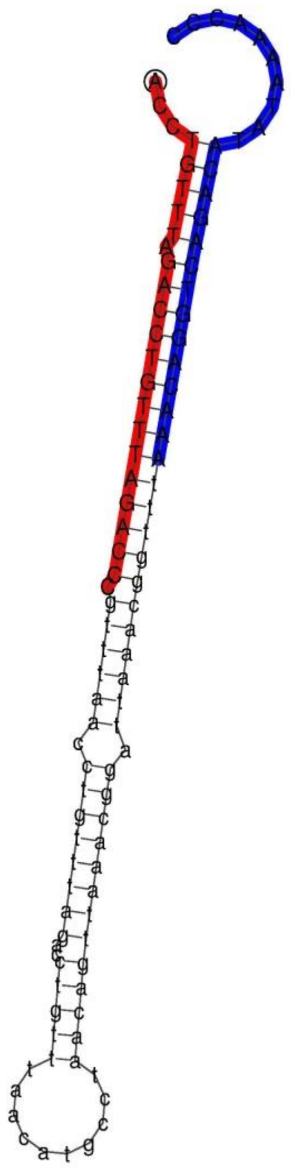

pda-3p-329752_16/pda-5p-183654_40

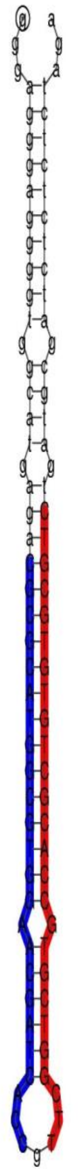

pda-5p-12958_1929/pda-3p-171233_45-3p-R16

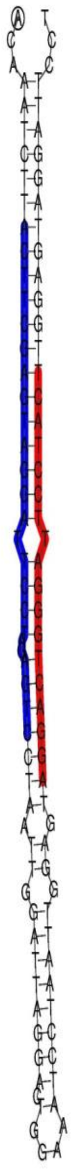

pda-5p-37236_491/pda-3p-138631_63

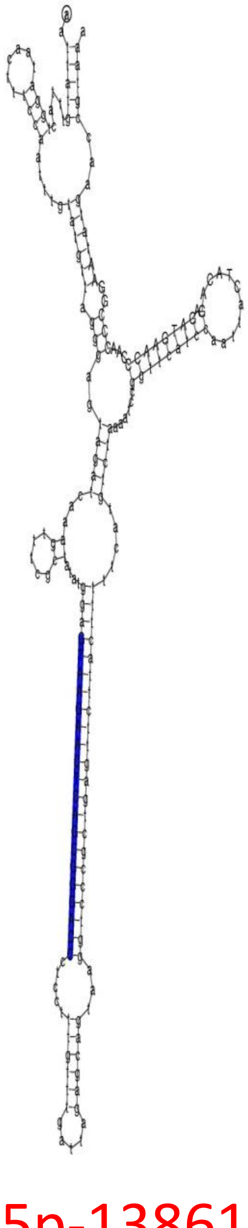

pda-5p-138615_63

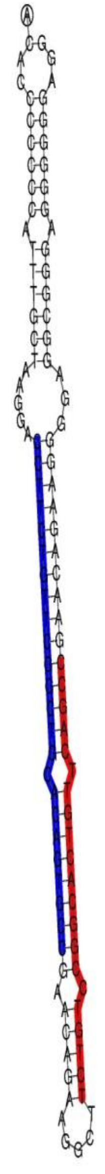

pda-5p-367016_14/pda-3p-21009_1073

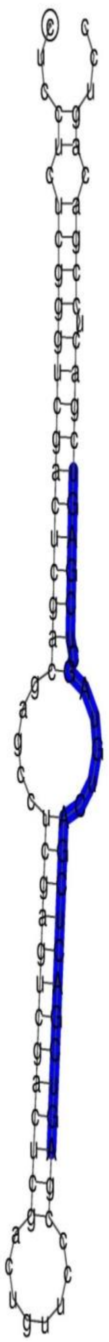

????

pda-3p-5998_4496

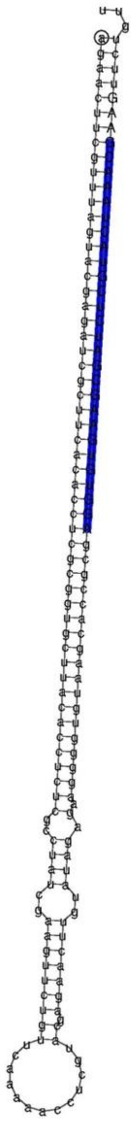

pda-3p-17663_1328

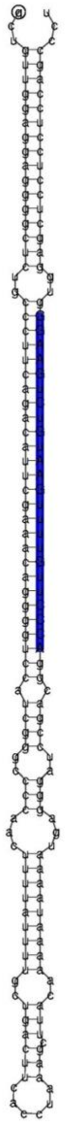

pda-3p-35797_519

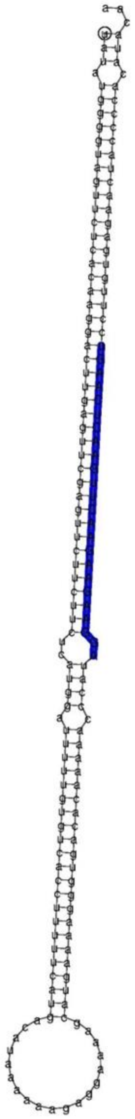

pda-3p-88602_130

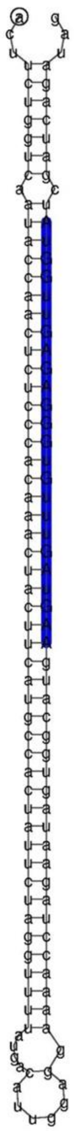

pda-3p-89170_129

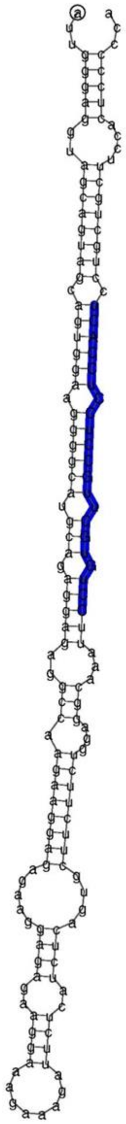

pda-3p-91106_124

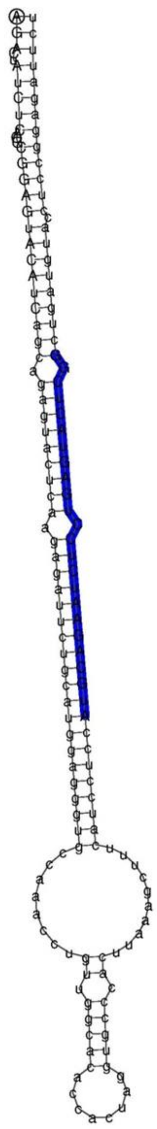

pda-3p-94240_118

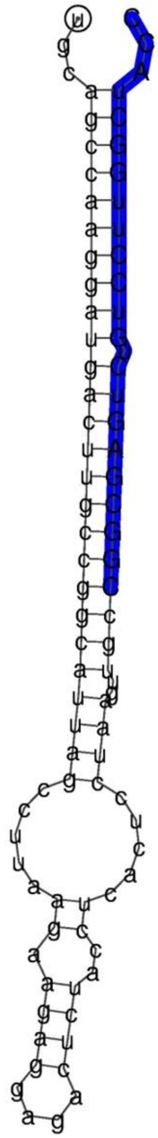

pda-3p-98773_109

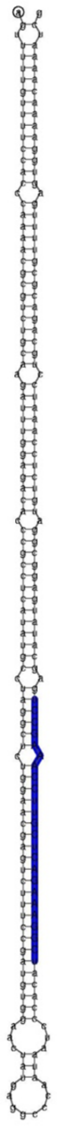

pda-3p-98930_109

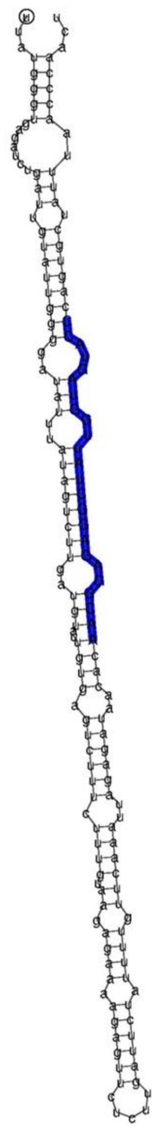

pda-3p-123856_76

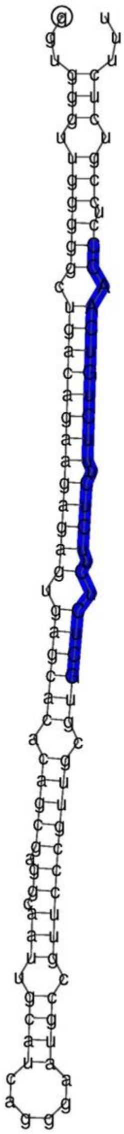

pda-3p-149076_56

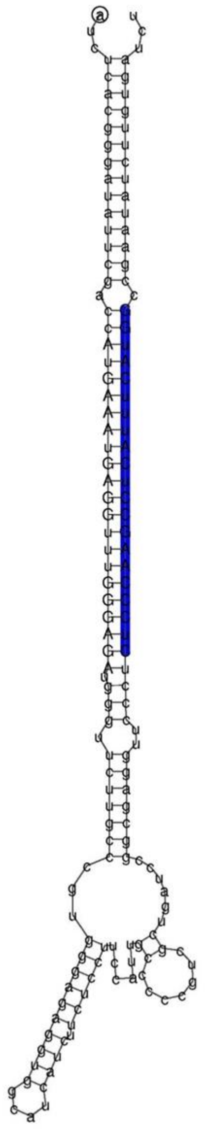

pda-3p-165824_47

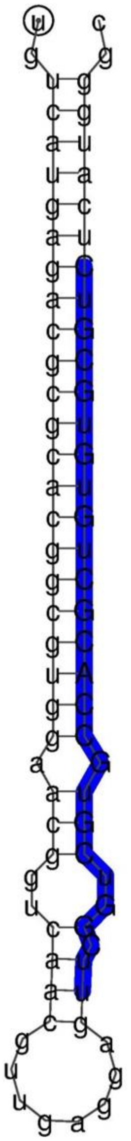

pda-3p-171233_45

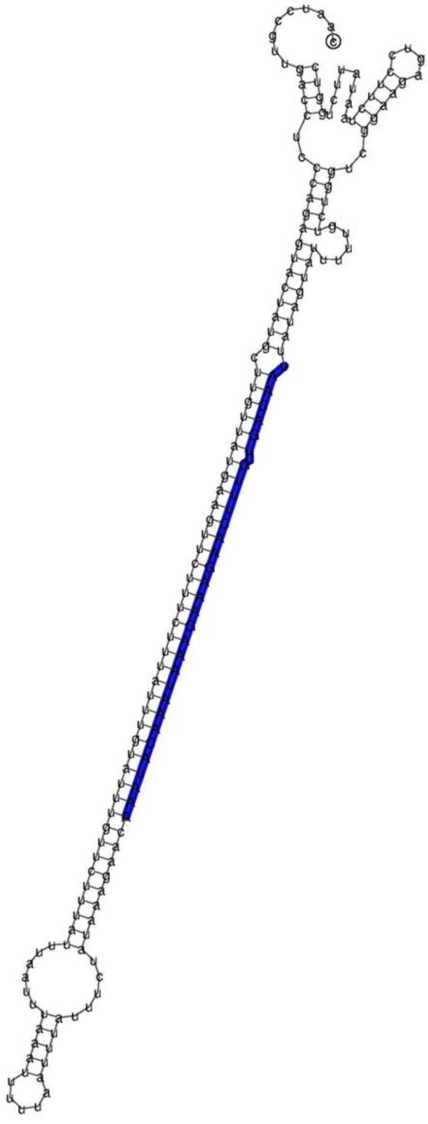

pda-3p-173080_44

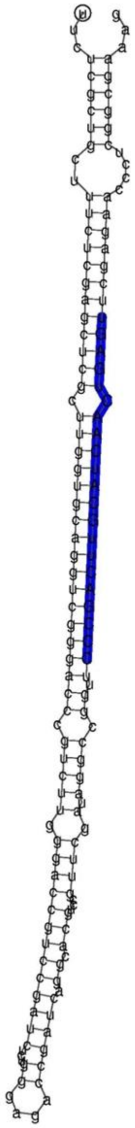

pda-3p-200031_35

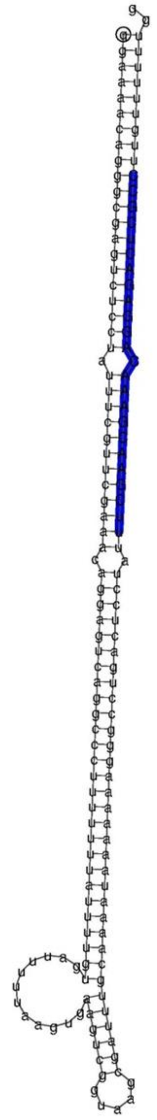

pda-3p-217326_31

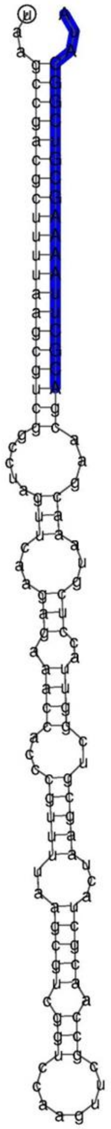

pda-3p-228238_28

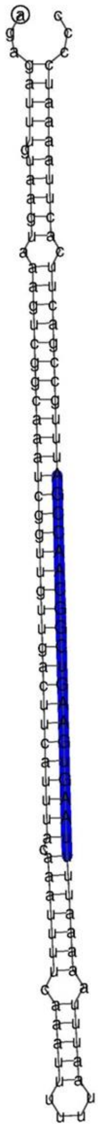

pda-3p-250092_25

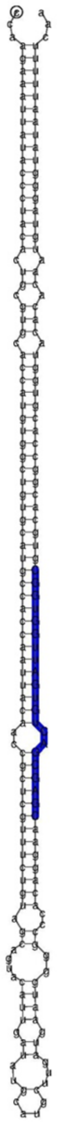

pda-3p-259371_23

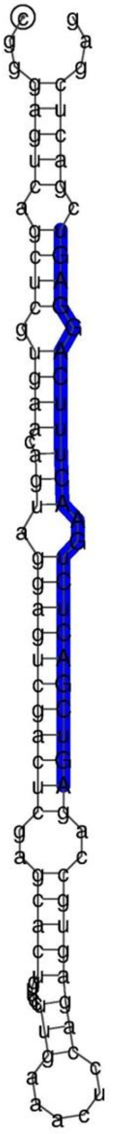

pda-3p-261846_23

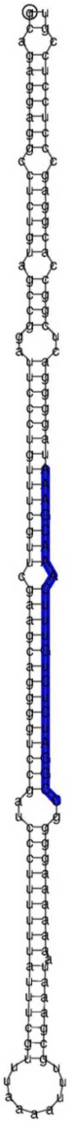

pda-3p-262764_23

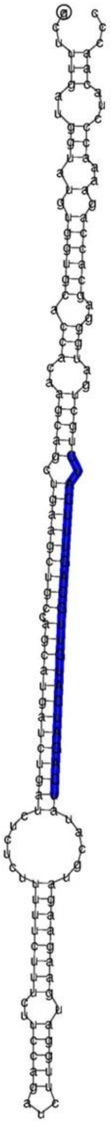

pda-3p-263356_23

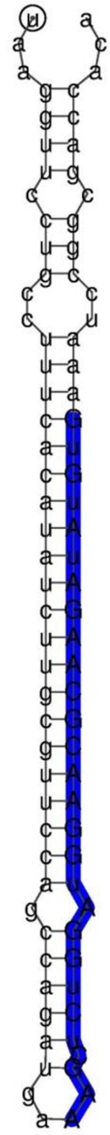

?????

pda-3p-265953_23

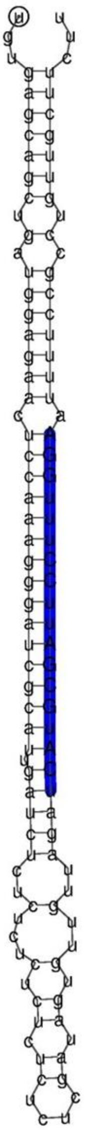

pda-3p-280606_21

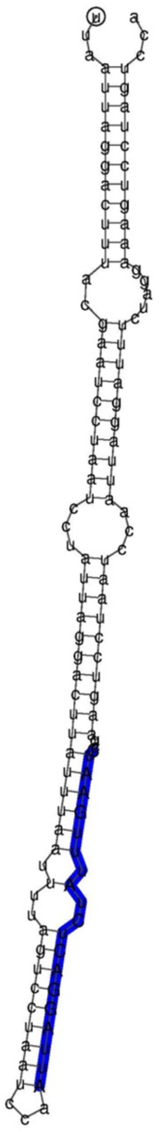

pda-3p-285375_20

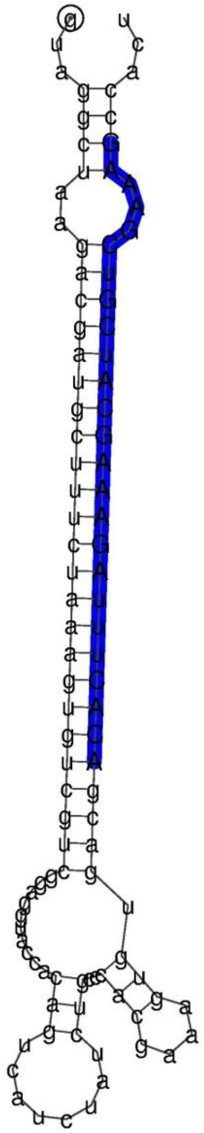

pda-3p-296980_19

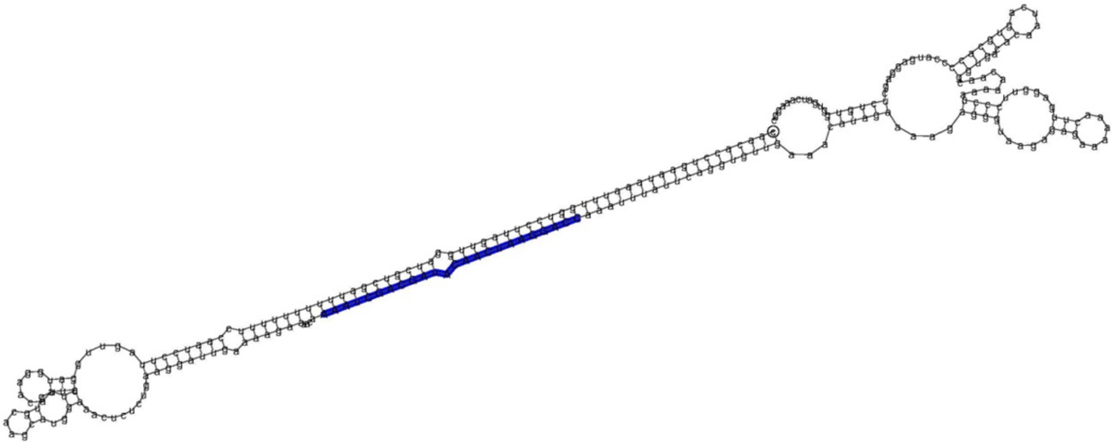

pda-3p-313473_18

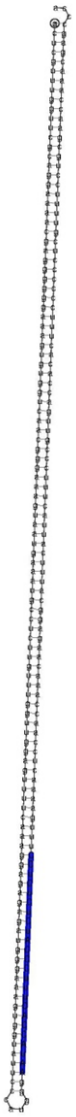

pda-3p-316346_17

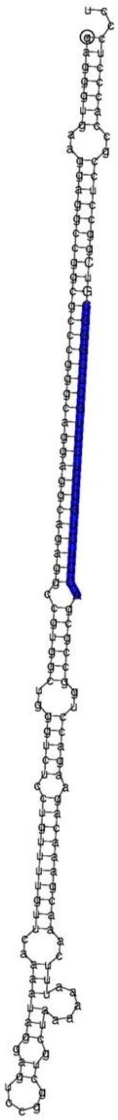

pda-3p-336513_16

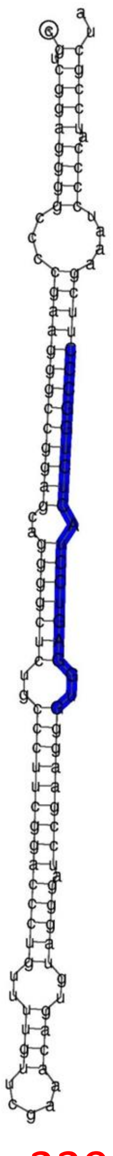

pda-3p-339262_16

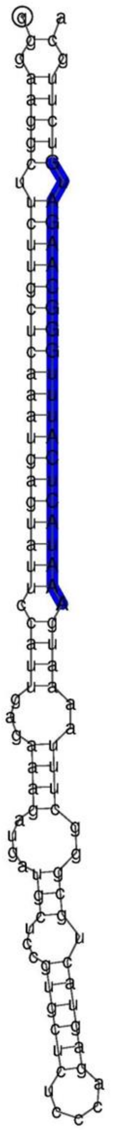

pda-3p-340060_16

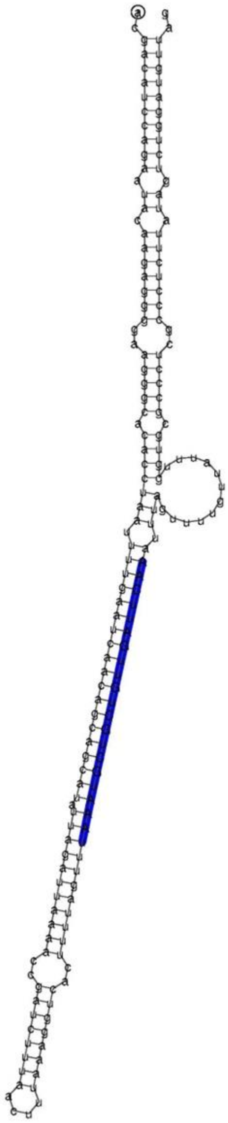

pda-3p-349991_15

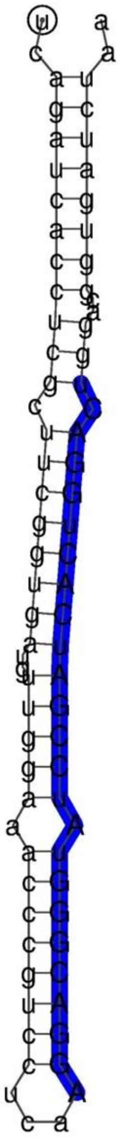

pda-3p-373581_14

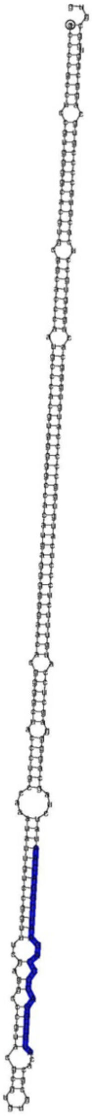

pda-3p-374825_14

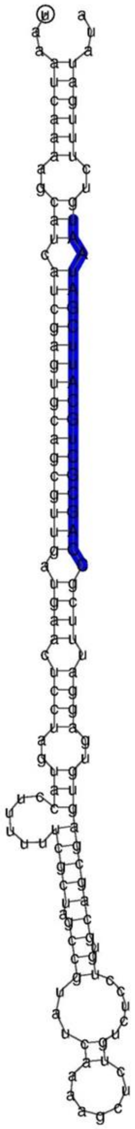

pda-3p-398260_13

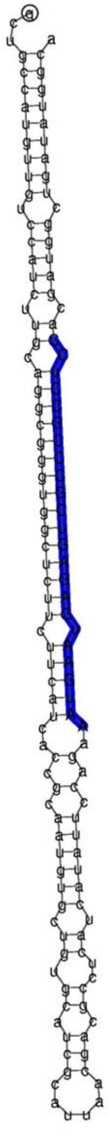

pda-3p-398802_13

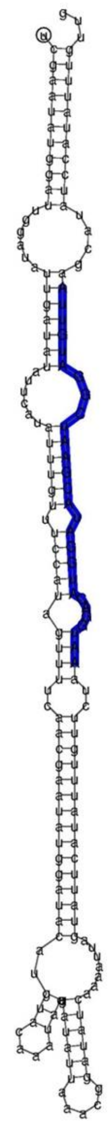

pda-3p-413562_12

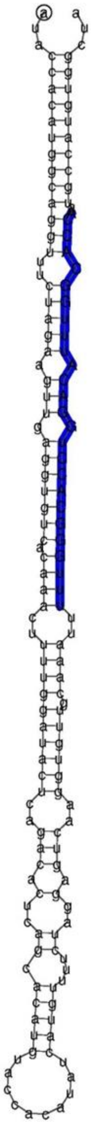

pda-3p-417972_12

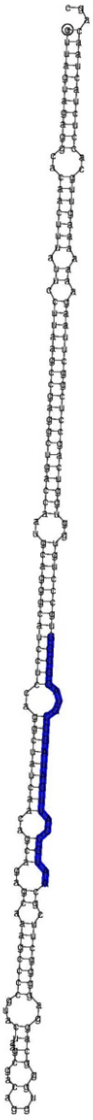

pda-3p-423087_12

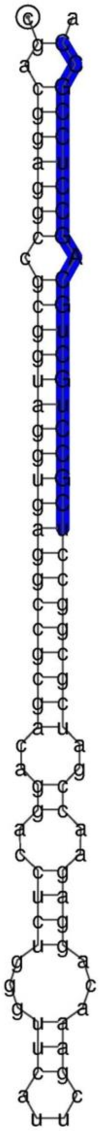

pda-3p-437970_11

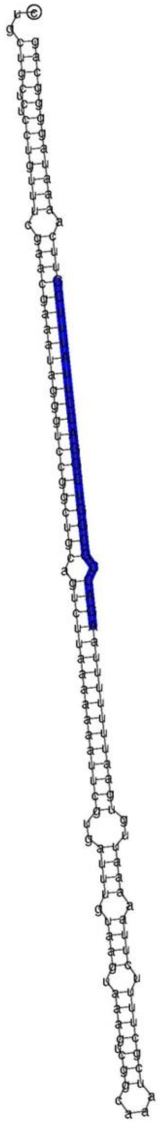

pda-3p-444722_11

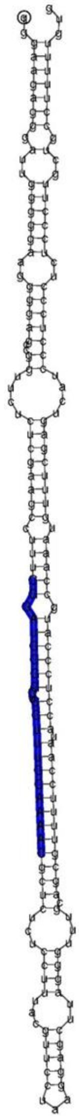

pda-5p-2741_10004

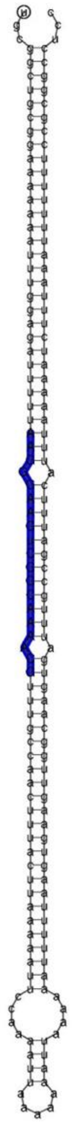

pda-5p-5696_4736

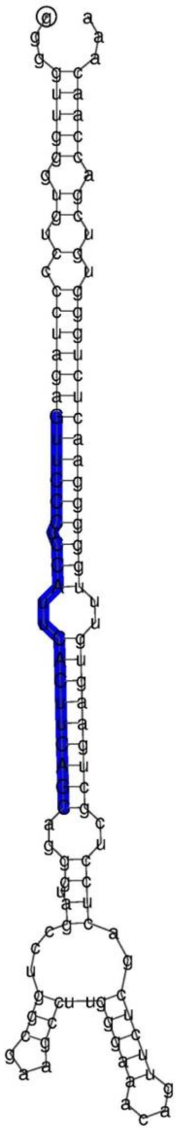

pda-5p-16728_1419

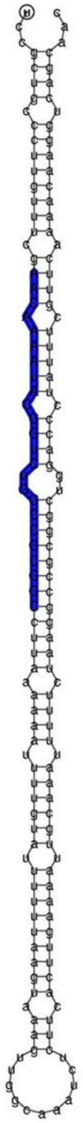

pda-5p-28278_721

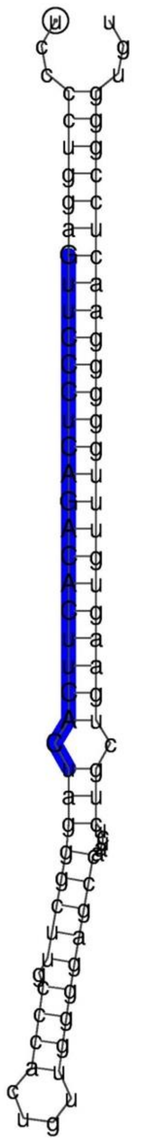

pda-5p-28659_707

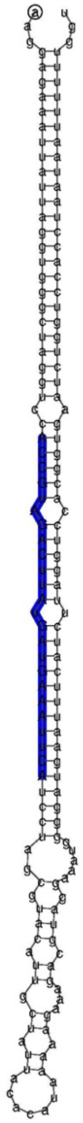

pda-5p-55341_272

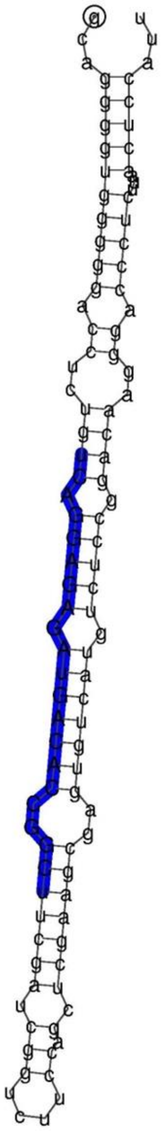

pda-5p-61161_232

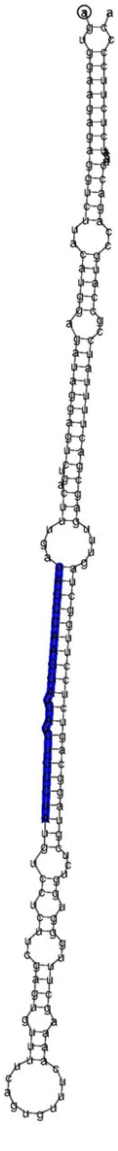

pda-5p-93628_119

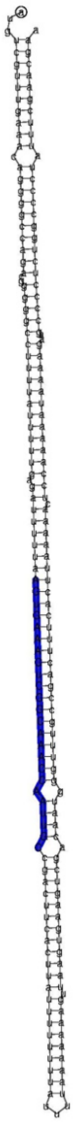

pda-5p-104109_100

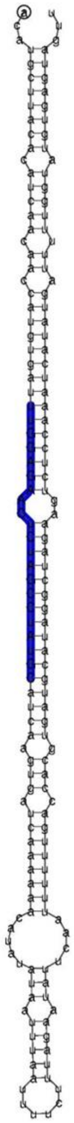

pda-5p-110153_92

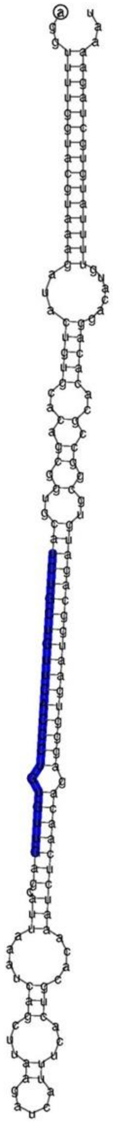

pda-5p-113778_87

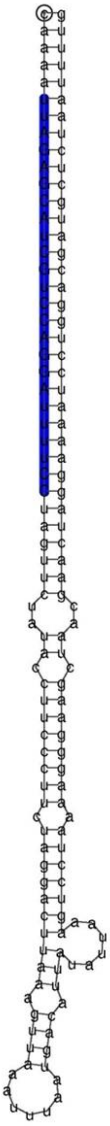

pda-5p-124075_75(closed)

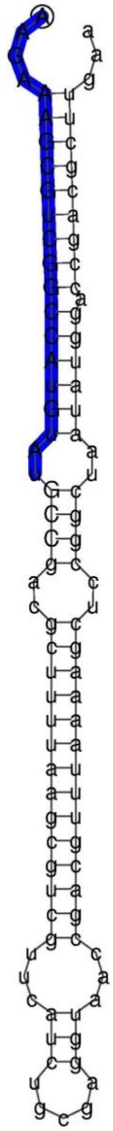

pda-5p-136470_65

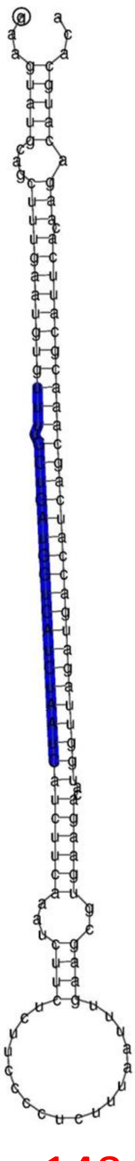

pda-5p-148449_56

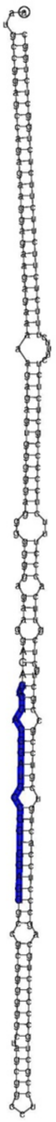

pda-5p-155899_52

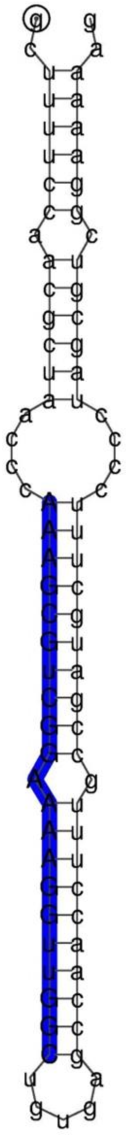

pda-5p-164396_48

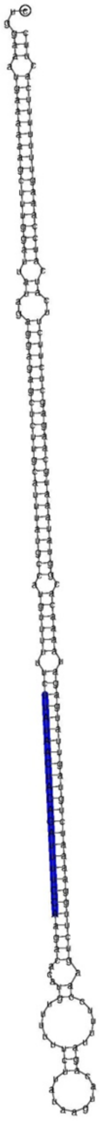

pda-5p-181586_41

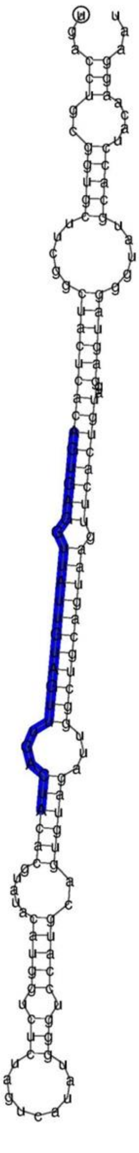

pda-5p-184243_40

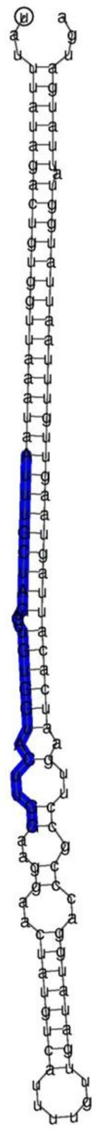

pda-5p-190444_38

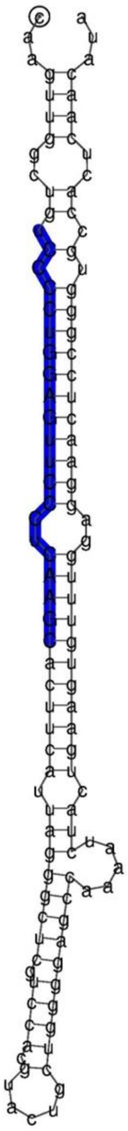

pda-5p-192728_37

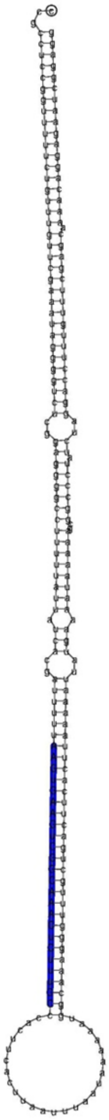

pda-5p-195988_36

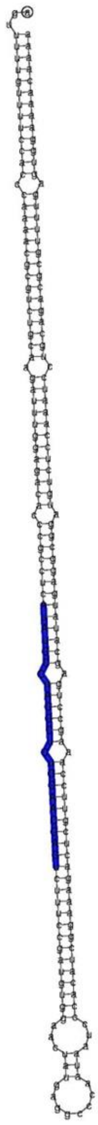

pda-5p-196234_36

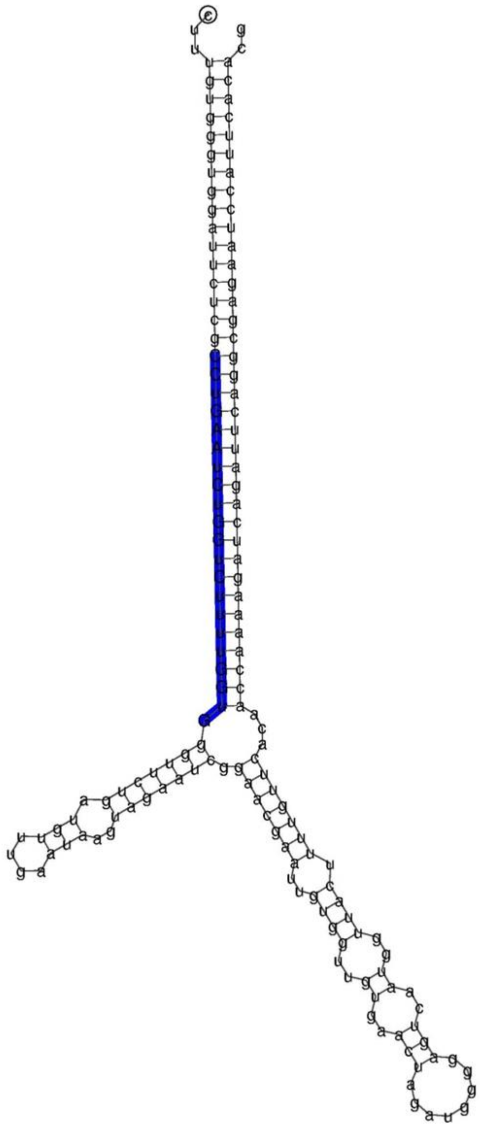

pda-5p-217347_31

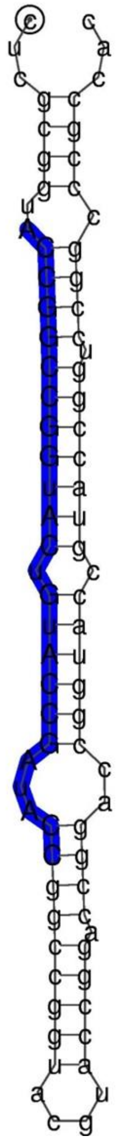

pda-5p-223801_29

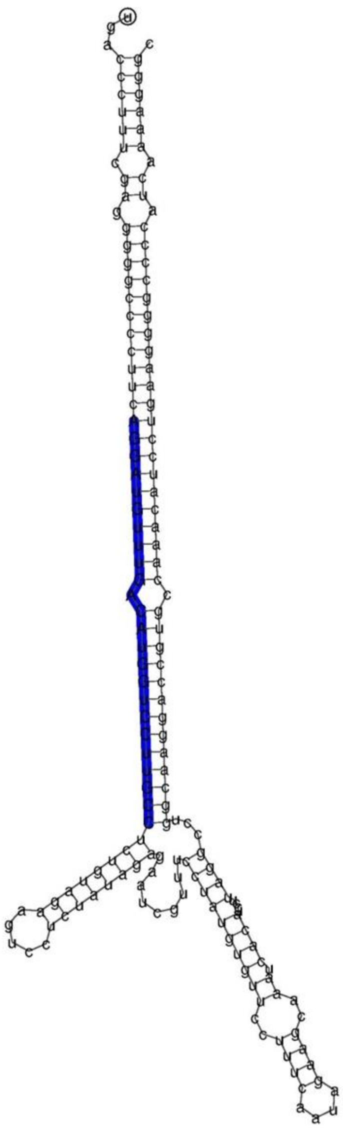

pda-5p-228808_28

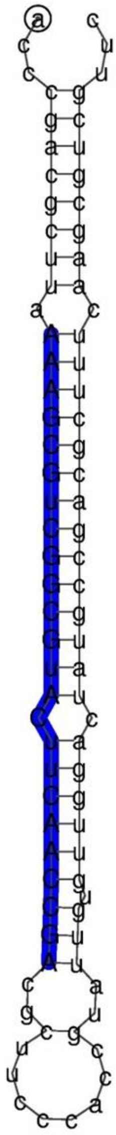

pda-5p-233999_27

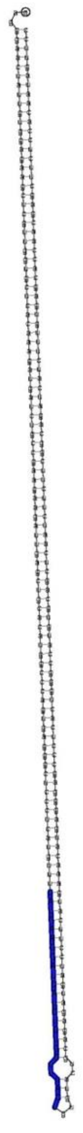

pda-5p-239350_26

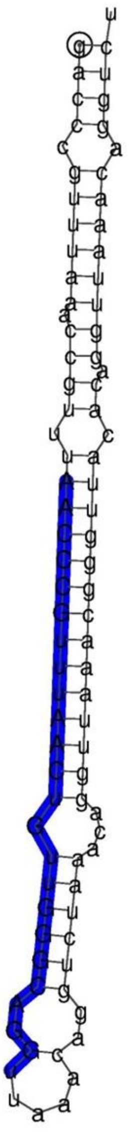

pda-5p-243982_26

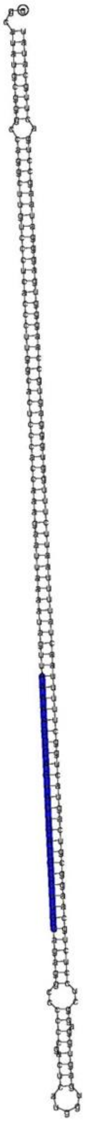

pda-5p-253816_24

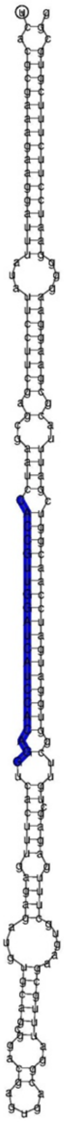

pda-5p-255507_24

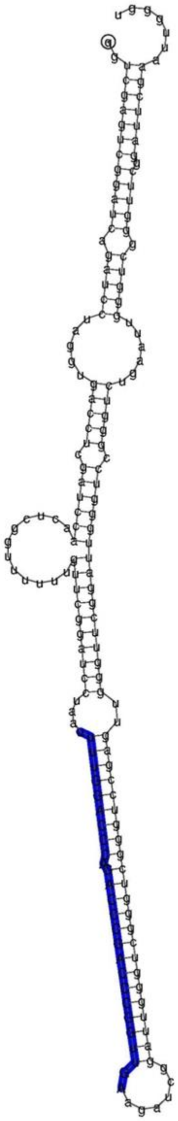

pda-5p-260633_23

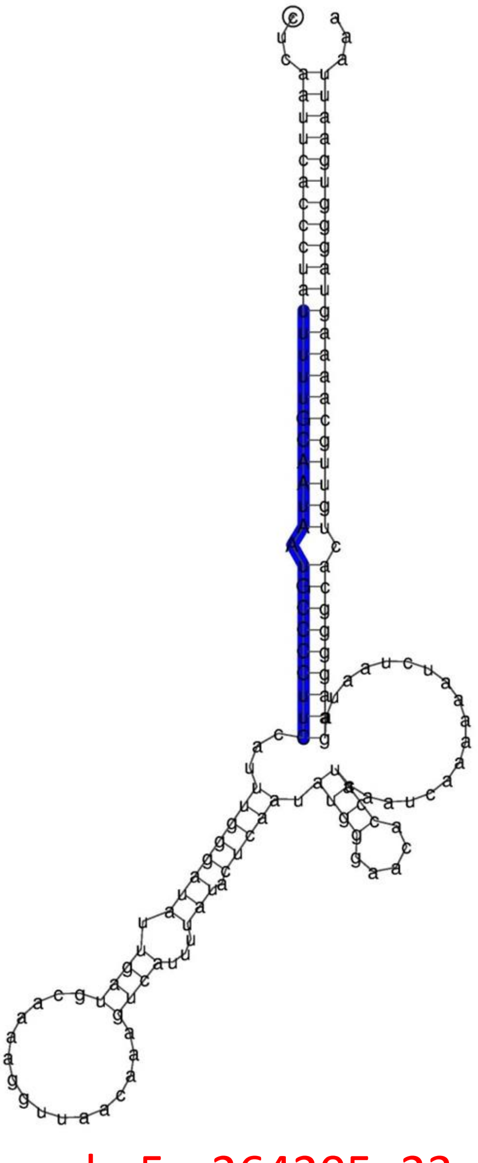

pda-5p-264205_23

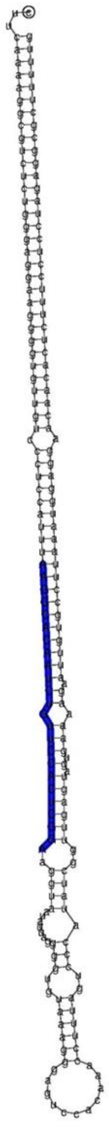

pda-5p-264380_23

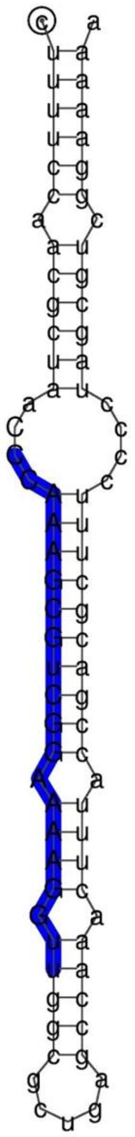

pda-5p-266573_22

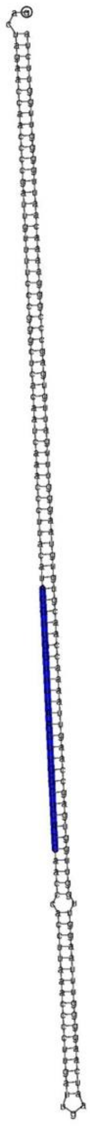

pda-5p-266719_22

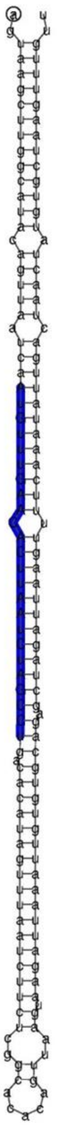

pda-5p-270991_22

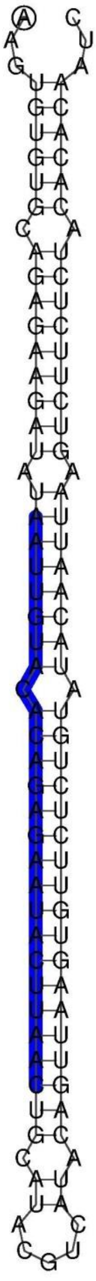

pda-5p-274880_21

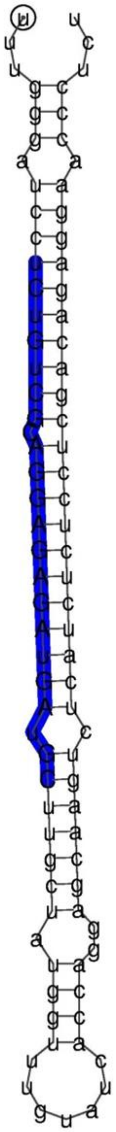

pda-5p-276361_21

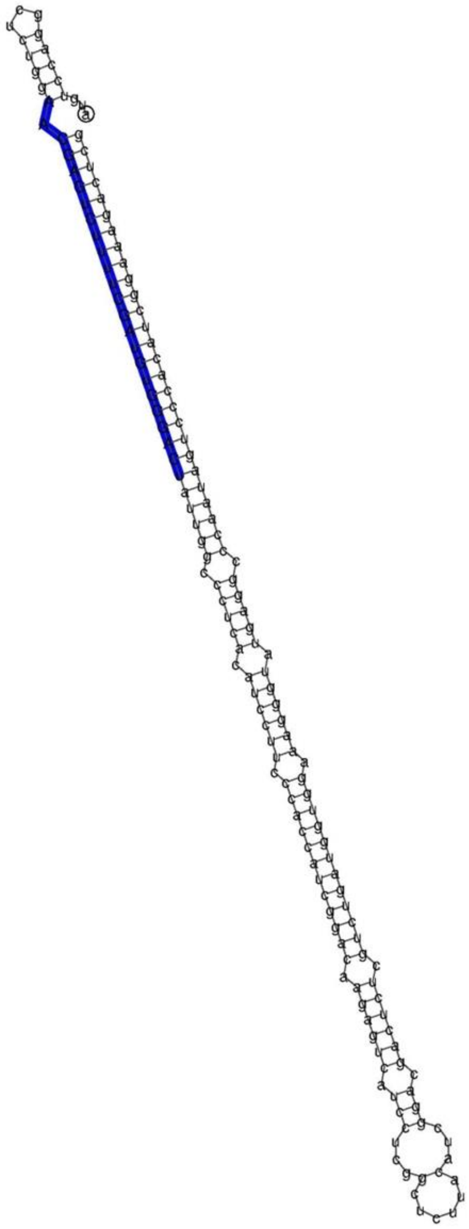

pda-5p-277581_21

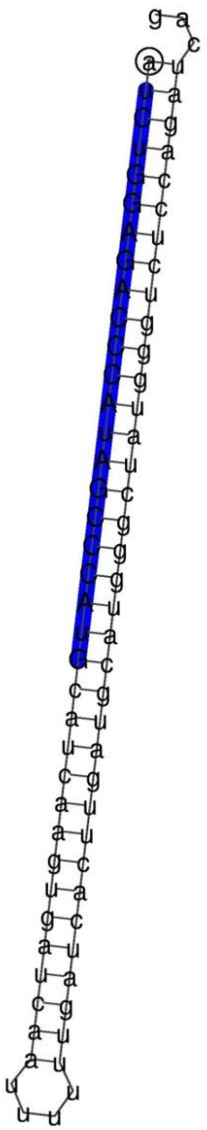

pda-5p-283724_20

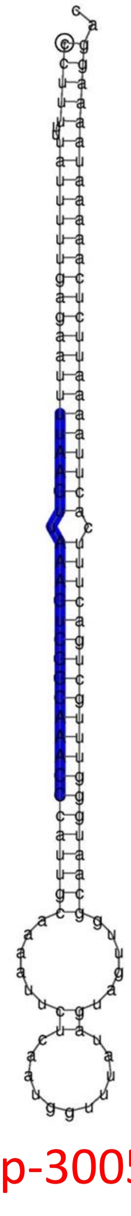

pda-5p-300513_19

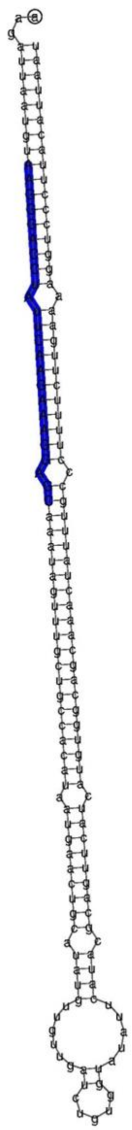

pda-5p-303859_18

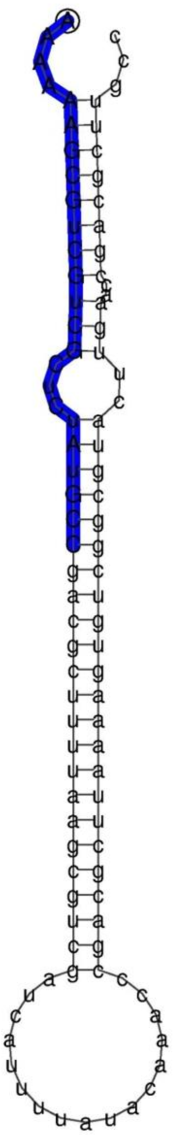

pda-5p-323301_17

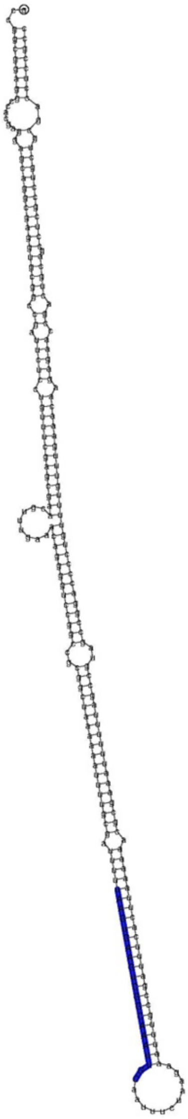

pda-5p-342543_16

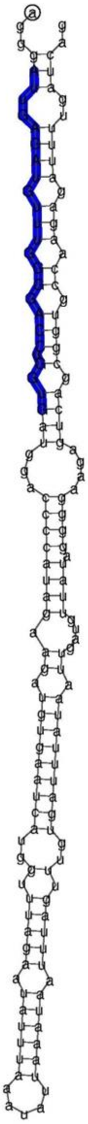

pda-5p-353741_15

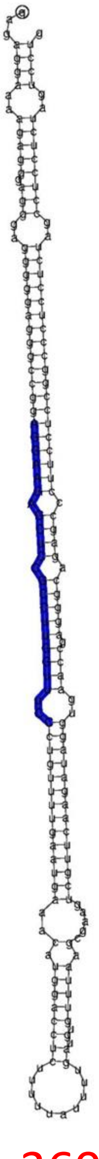

pda-5p-360119_14

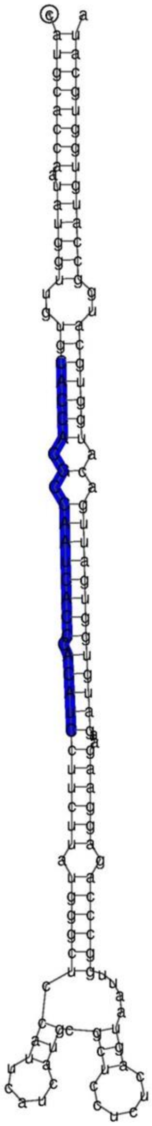

pda-5p-362724_14

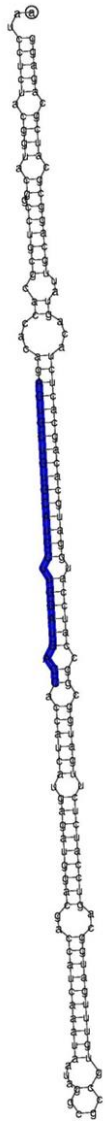

pda-5p-364804_14

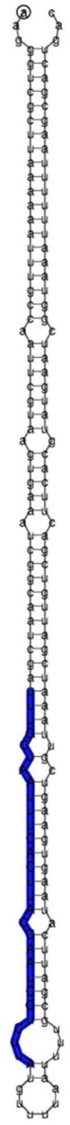

pda-5p-376037_14

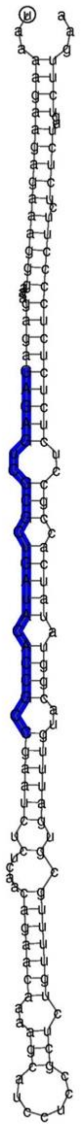

pda-5p-382296_13

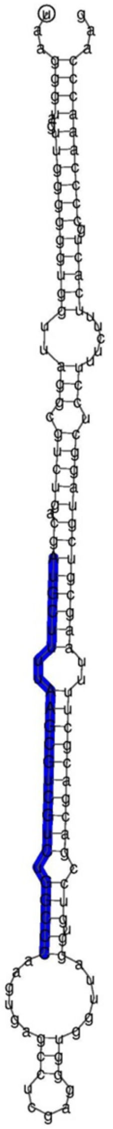

pda-5p-403658_12

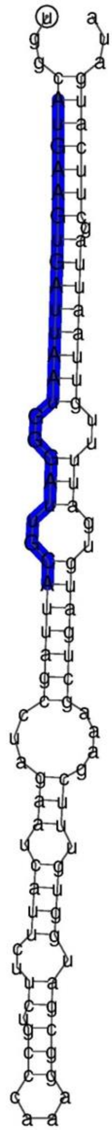

pda-5p-414524_12

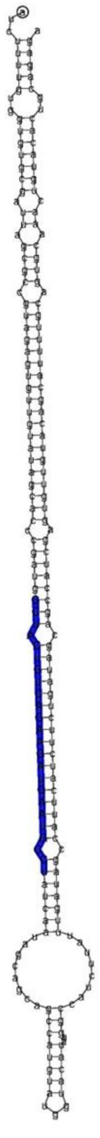

pda-5p-418800_12

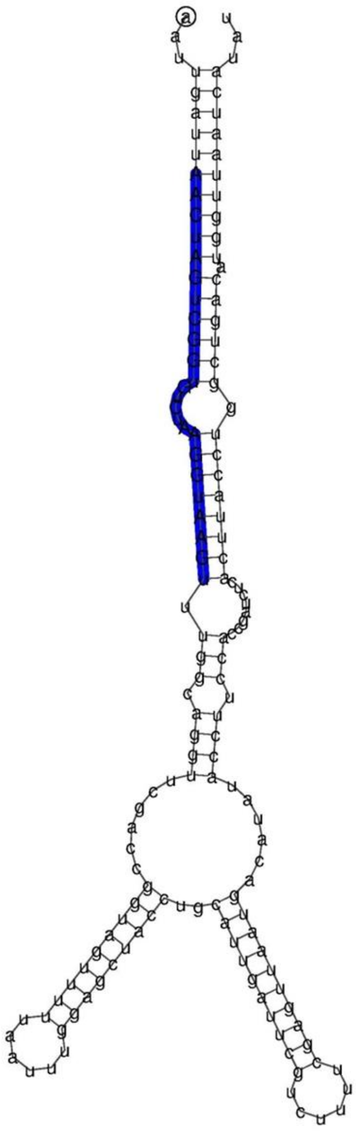

pda-5p-421095_12

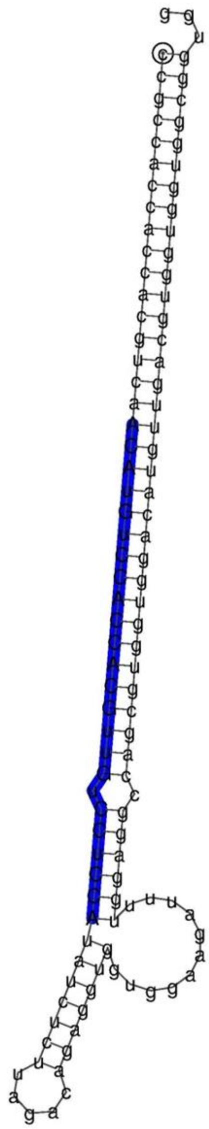

pda-5p-424271_12

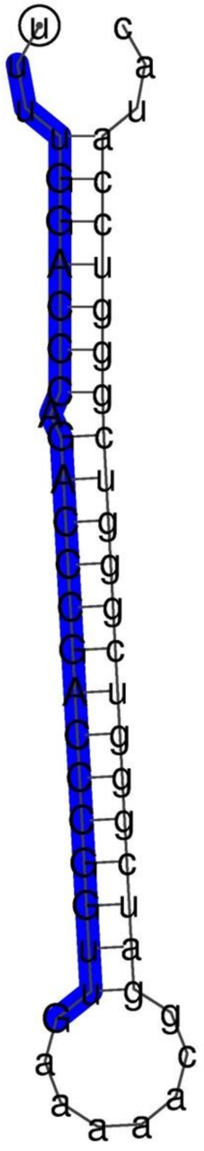

pda-5p-427445_11

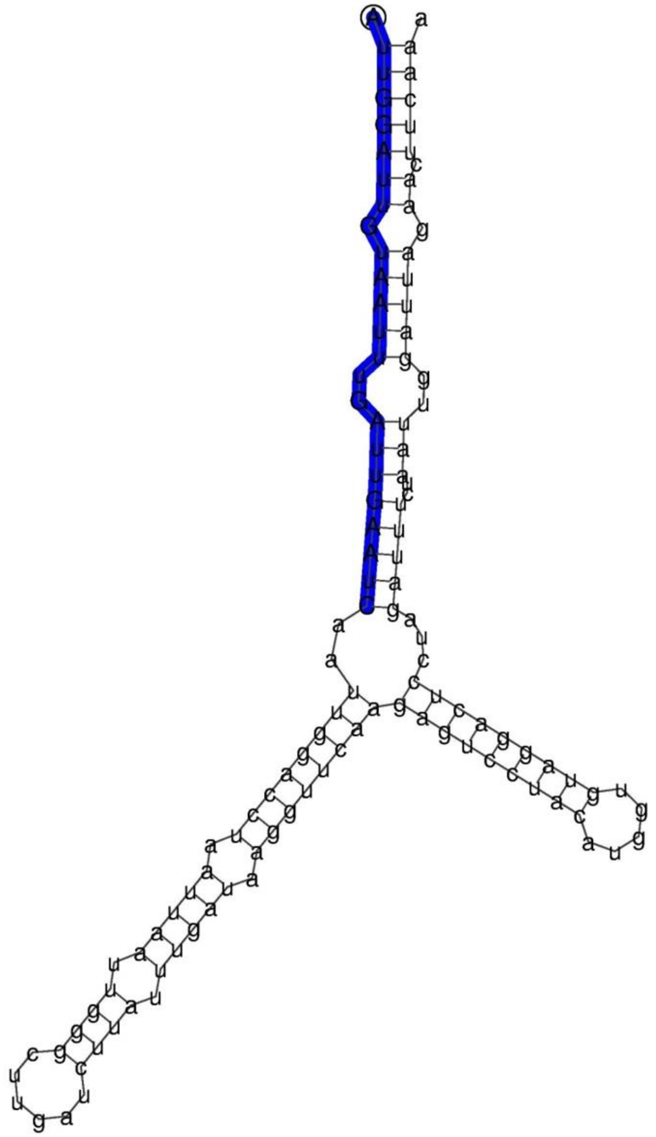

pda-5p-432618_11

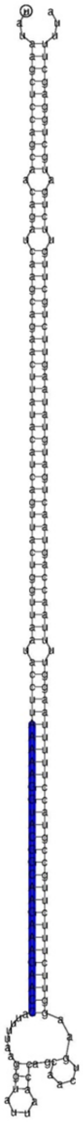

pda-5p-441054_11

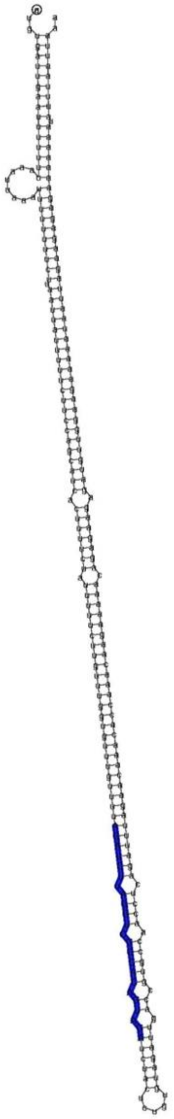

pda-5p-448267_11

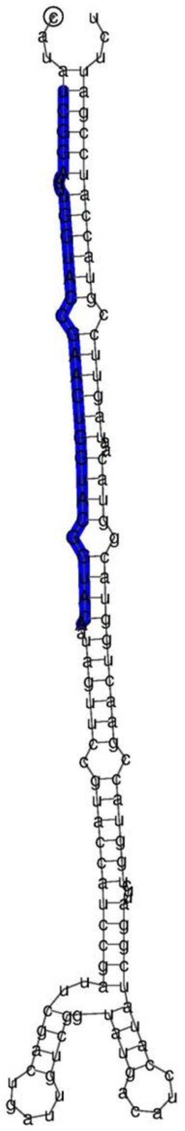

pda-5p-458916_10

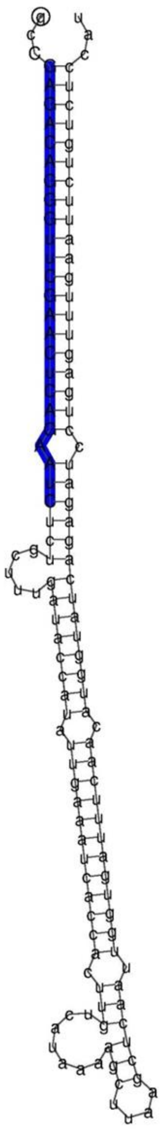

pda-5p-482466_10

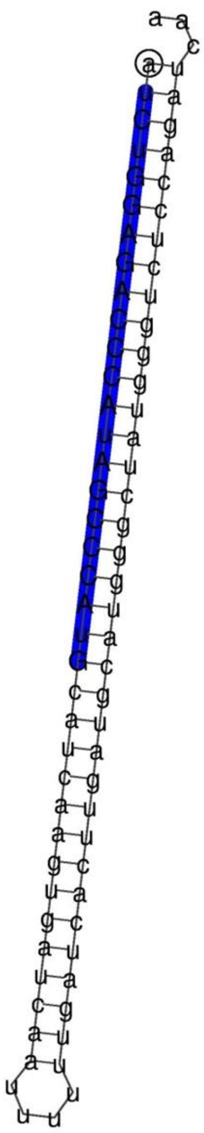

pda-5p-283724_20-R1

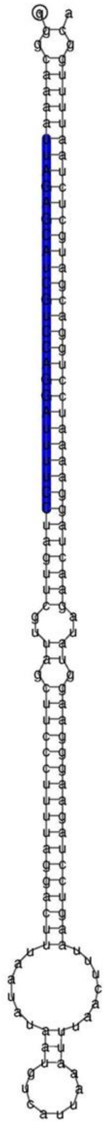

pda-5p-124075_75-R6

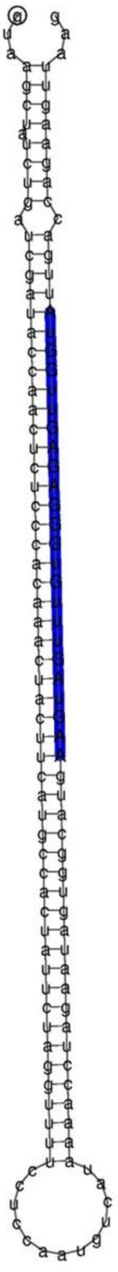

pda-3p-89170_129-R7

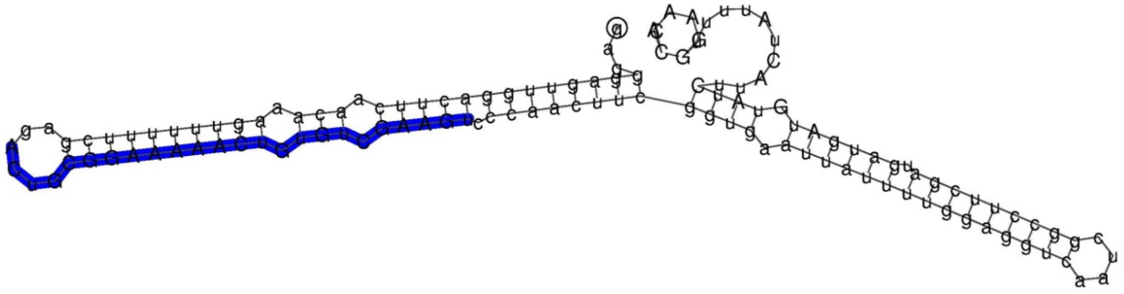

pda-5p-52910_291-R9

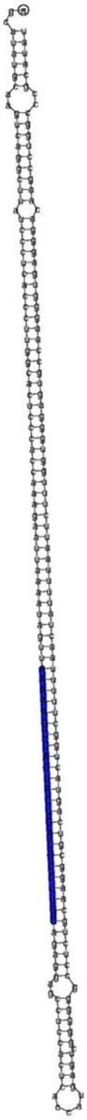

pda-5p-253816_24-R11

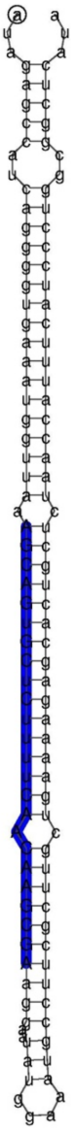

pda-5p-231728_28-R12

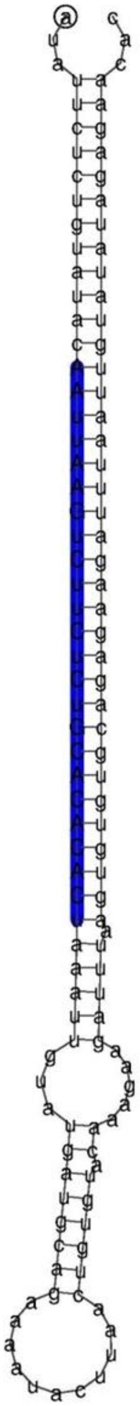

pda-5p-400528_12-R13

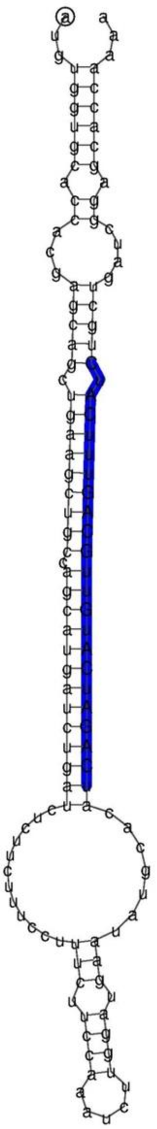

pda-3p-263356_23-R14

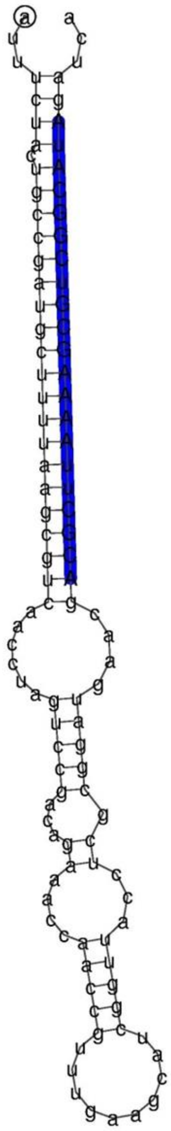

pda-3p-228238_28-R15

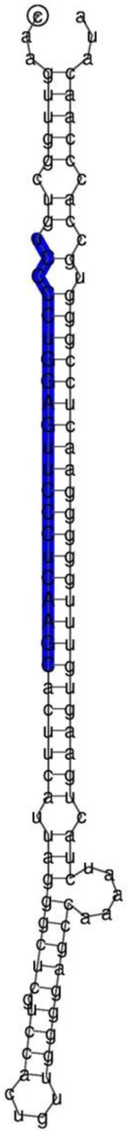

pda-5p-192728_37-R17

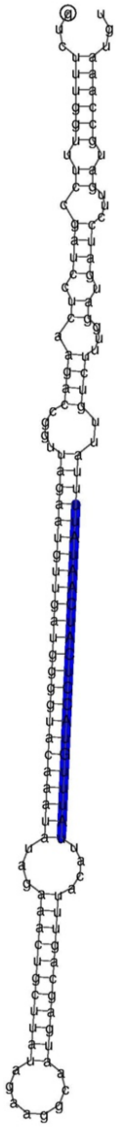

pda-3p-246312_25-R19

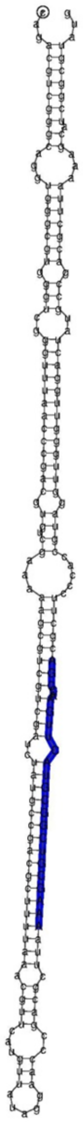

pda-5p-233999_27-R21

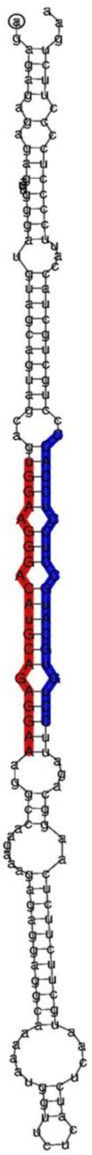

pda-3p-93572_119/pda-5p-85498_137

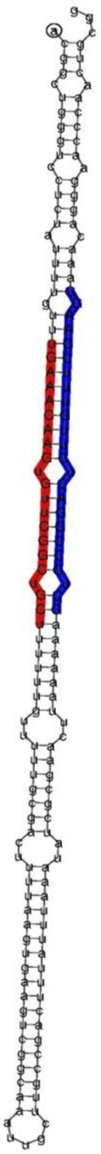

pda-3p-107067_96/pda-5p-311908_18

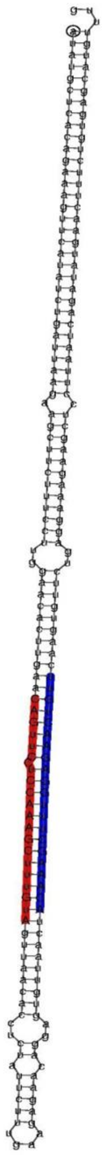

pda-3p-130659_69/pda-5p-224392_29

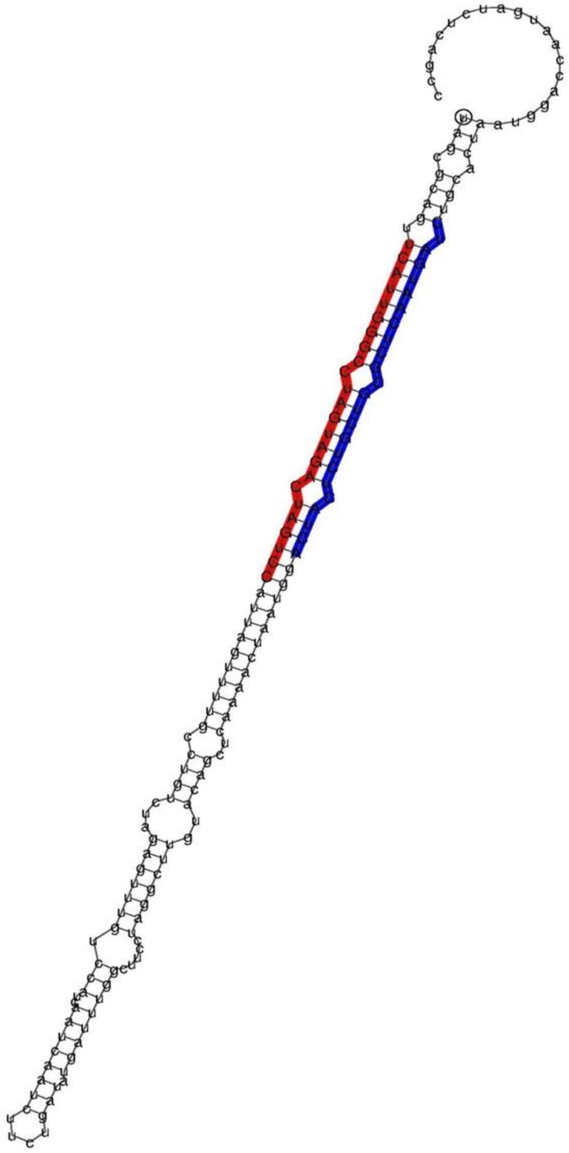

pda-3p-269912_22/pda-5p-310639_18

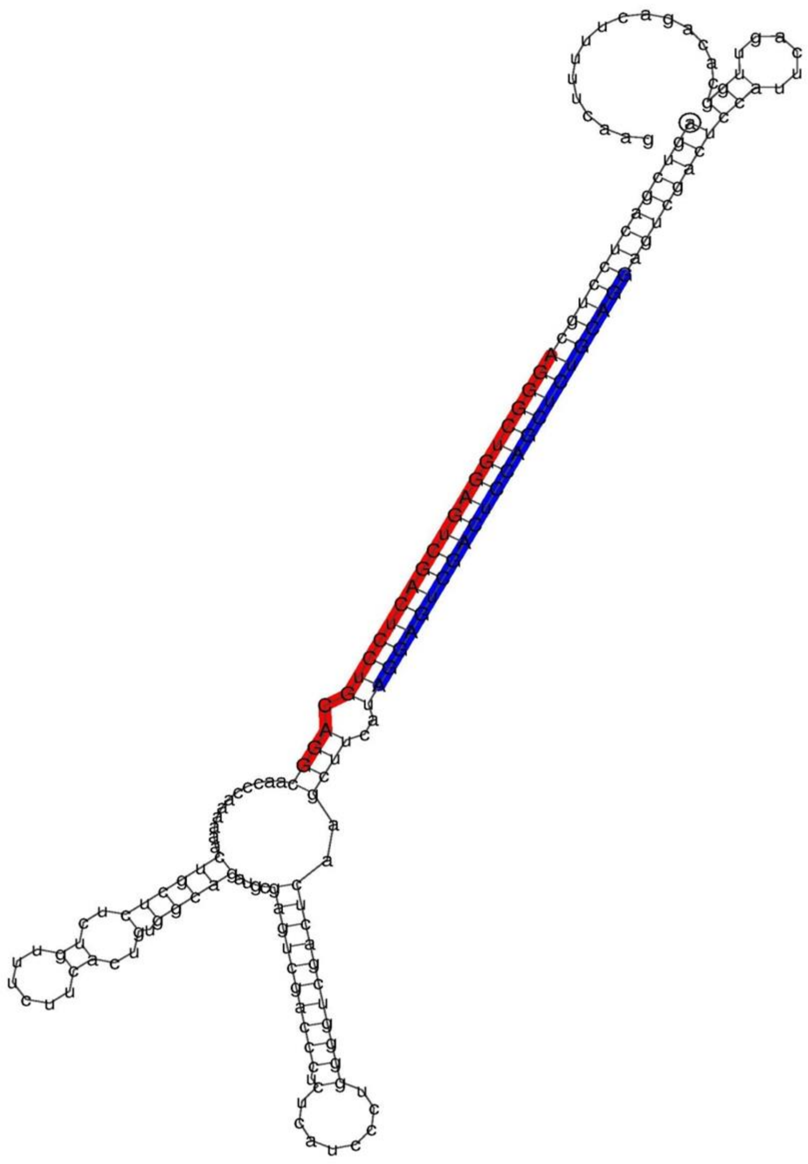

pda-3p-313824_18/pda-5p-321868_17

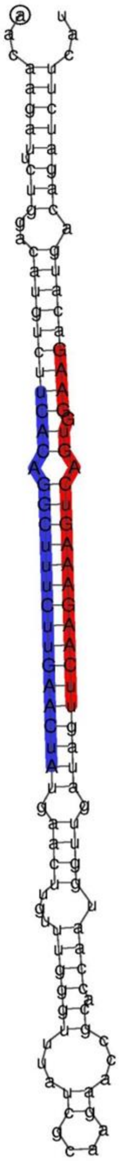

pda-5p-15197_1588 pda-3p-44090_382

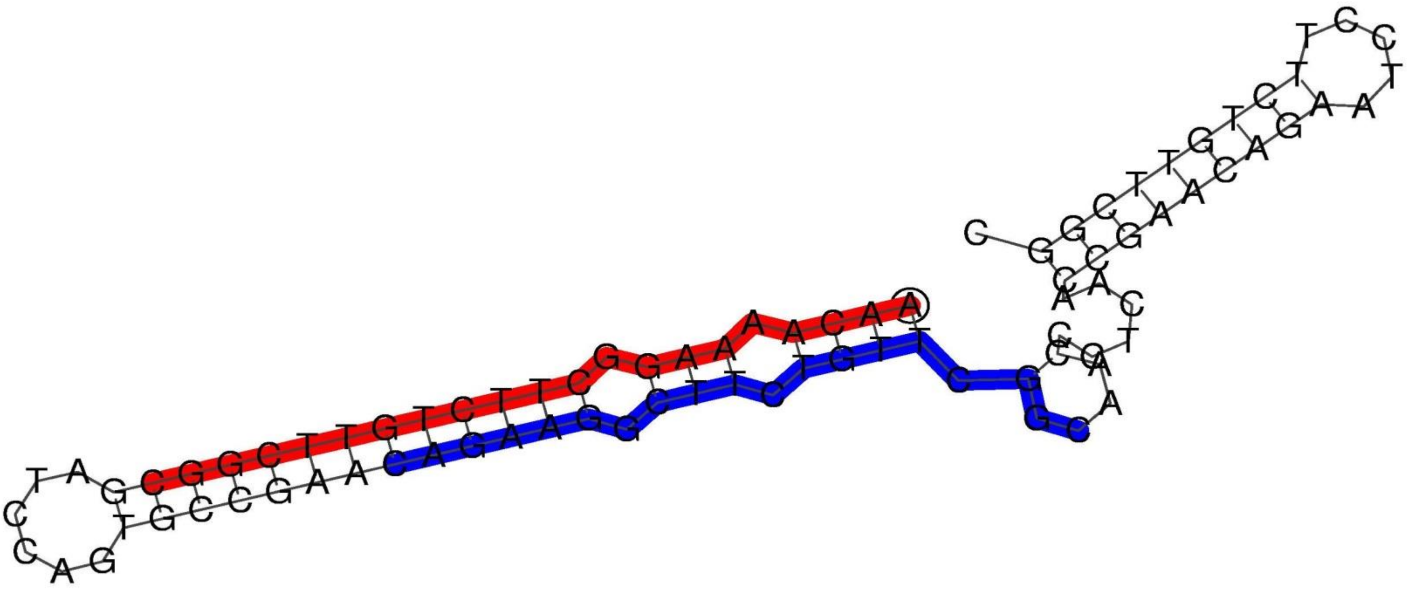

pda-5p-131814_68/ pda-3p-231836_28

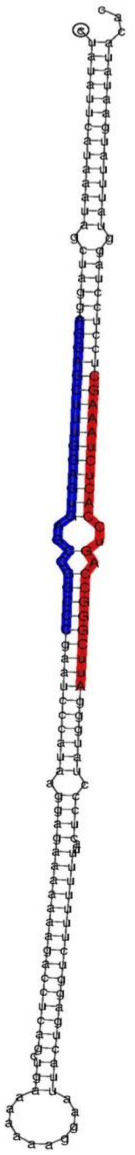

pda-5p-295079_19/ pda-3p-99813_107

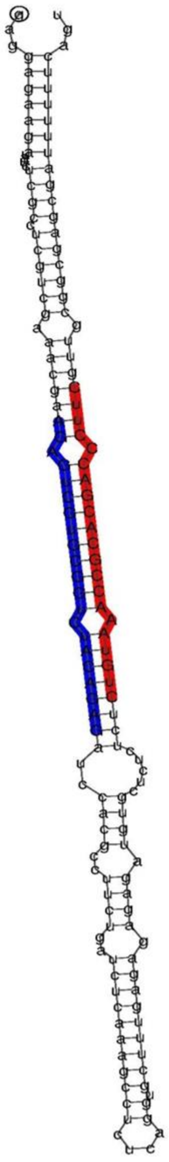

pda-5p-396570_13/pda-3p-8373_3137

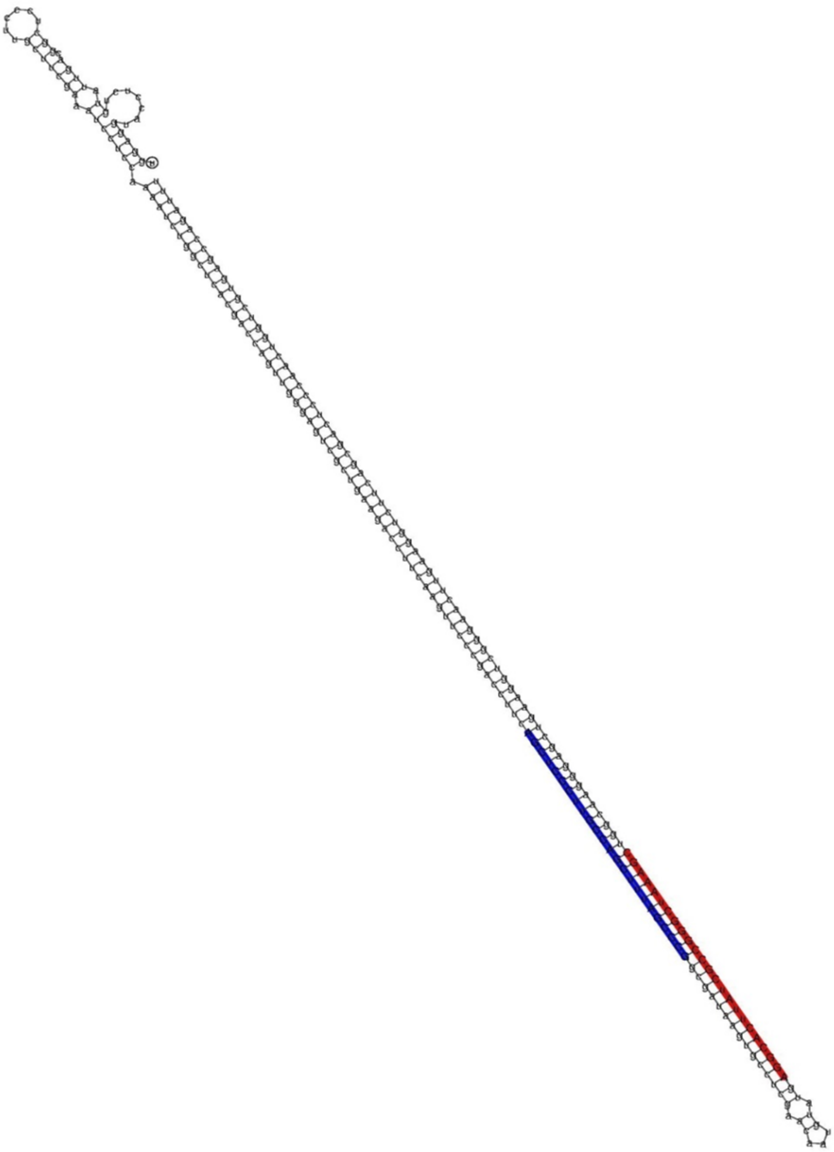

pda-5p-85614_137-R2/pda-3p-117840_82-R3

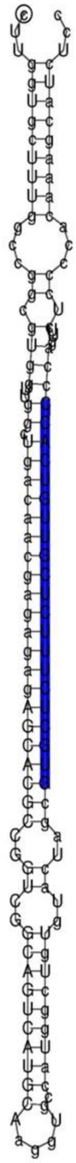

pda-3p-64787_213

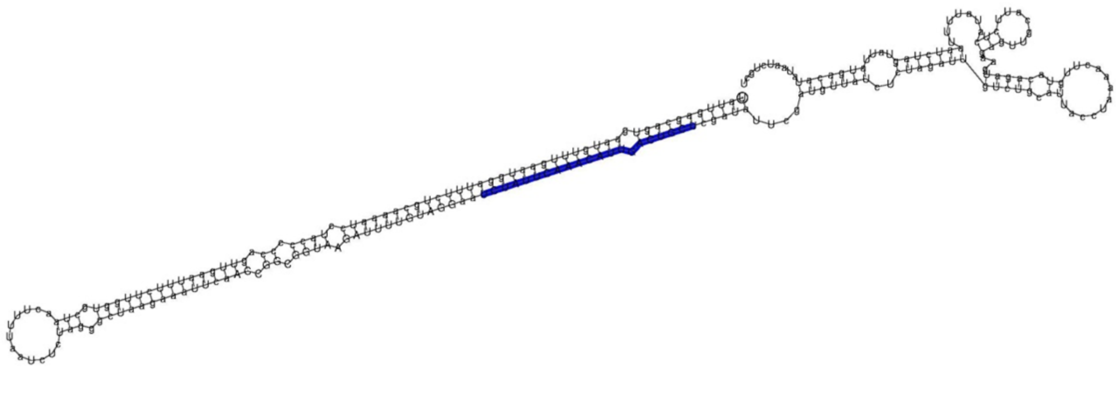

pda-3p-64863_213

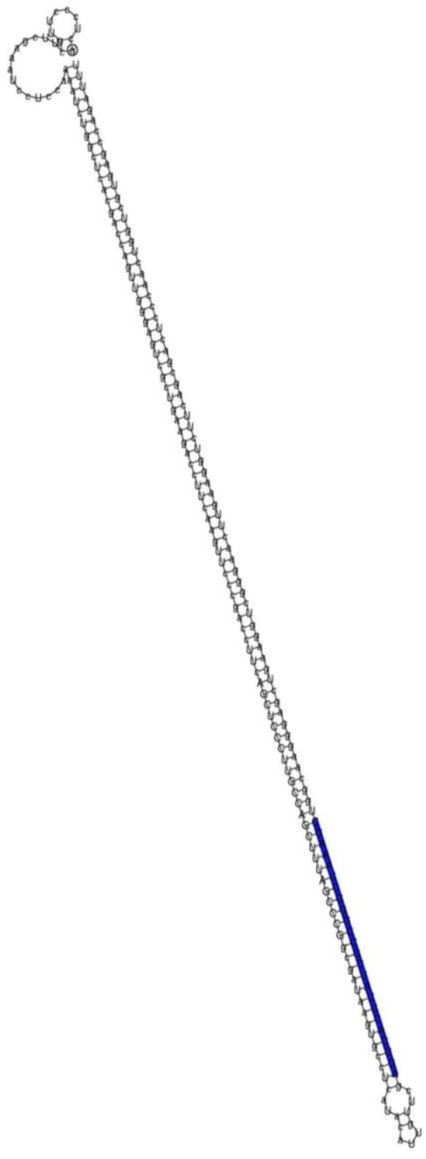

pda-3p-117840_82

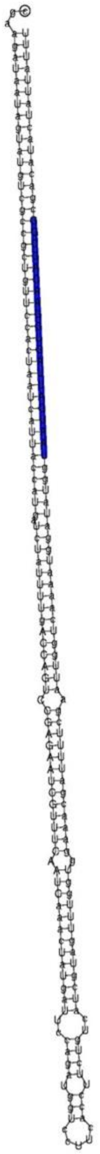

pda-3p-290154_20

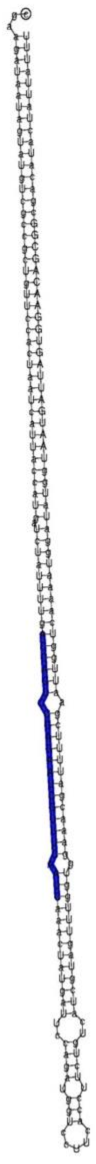

pda-5p-83202_144

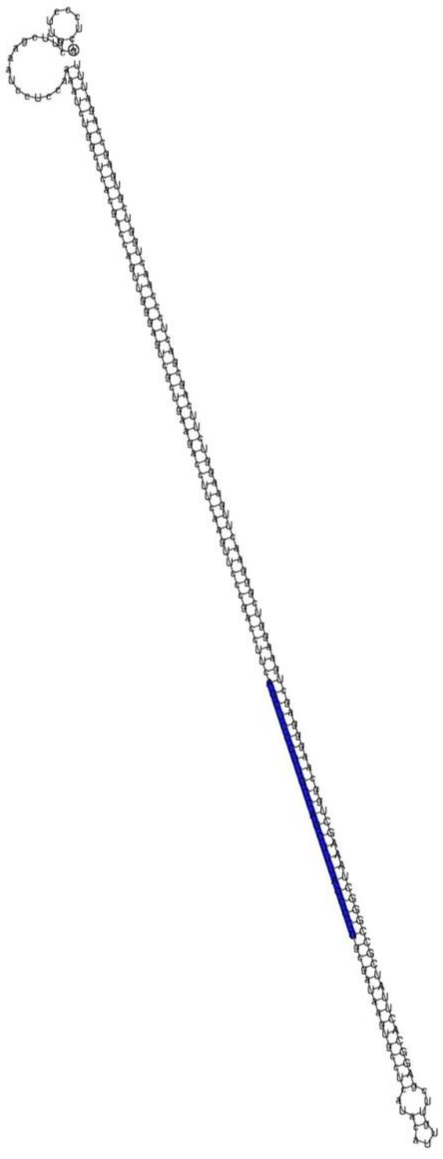

pda-5p-85614_137

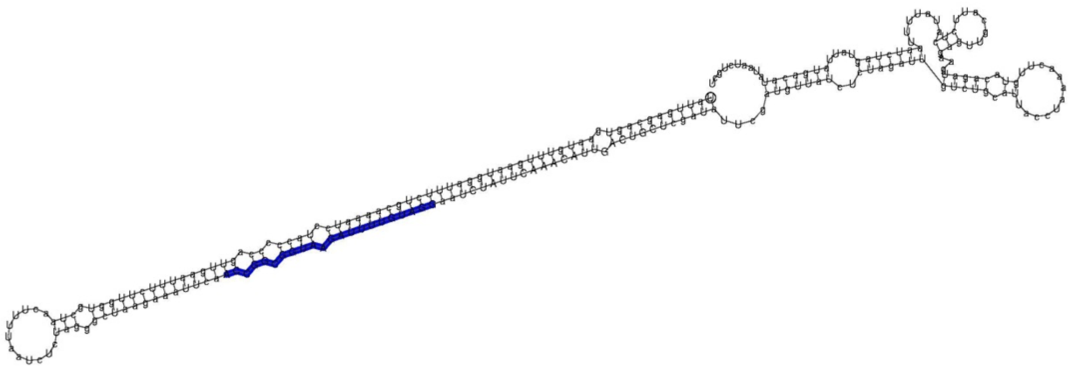

pda-5p-152404_54

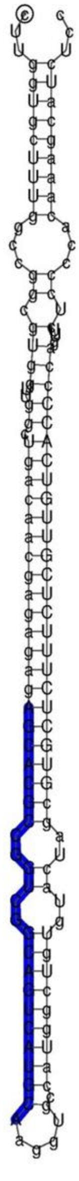

pda-5p-178899_42

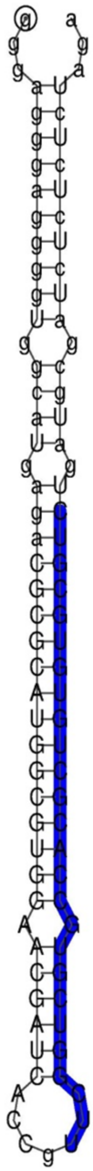

pda-3p-171233_45-R16
