# Supplementary material for: A genome-wide identification of the miRNAome in response to salinity stress in date palm (Phoenix dactylifera L.)
Source: Front Plant Sci. 2015 Nov 5;6:946. doi: 10.3389/fpls.2015.00946 (PMC4633500; doi:10.3389/fpls.2015.00946)
Supplement: Supplementary file 10 [file Image2.PDF]

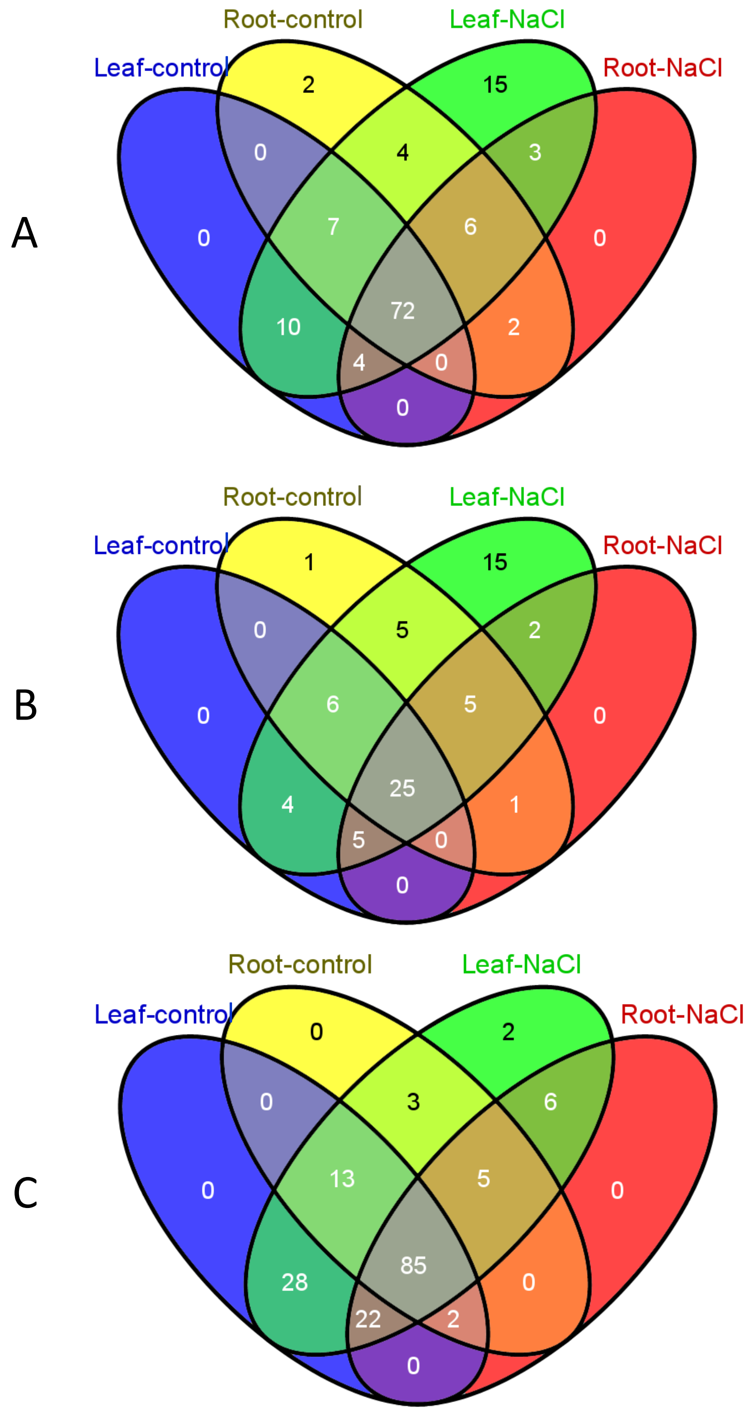

Figure S2: Venn Diagrams illustrate the number of conserved (A), variants (B) and potentially novel (C) miRNA identified in untreated and NaCl-leaves as well as untreated and NaCl-treated roots sRNA libraries. The numbers that are located in the overlapping area represent the number of shared miRNA in different libraries.
