# Supplementary figures and images for: A genome-wide identification of the miRNAome in response to salinity stress in date palm (Phoenix dactylifera L.)
Source: Front Plant Sci. 2015 Nov 5;6:946. doi: 10.3389/fpls.2015.00946 (PMC4633500; doi:10.3389/fpls.2015.00946)

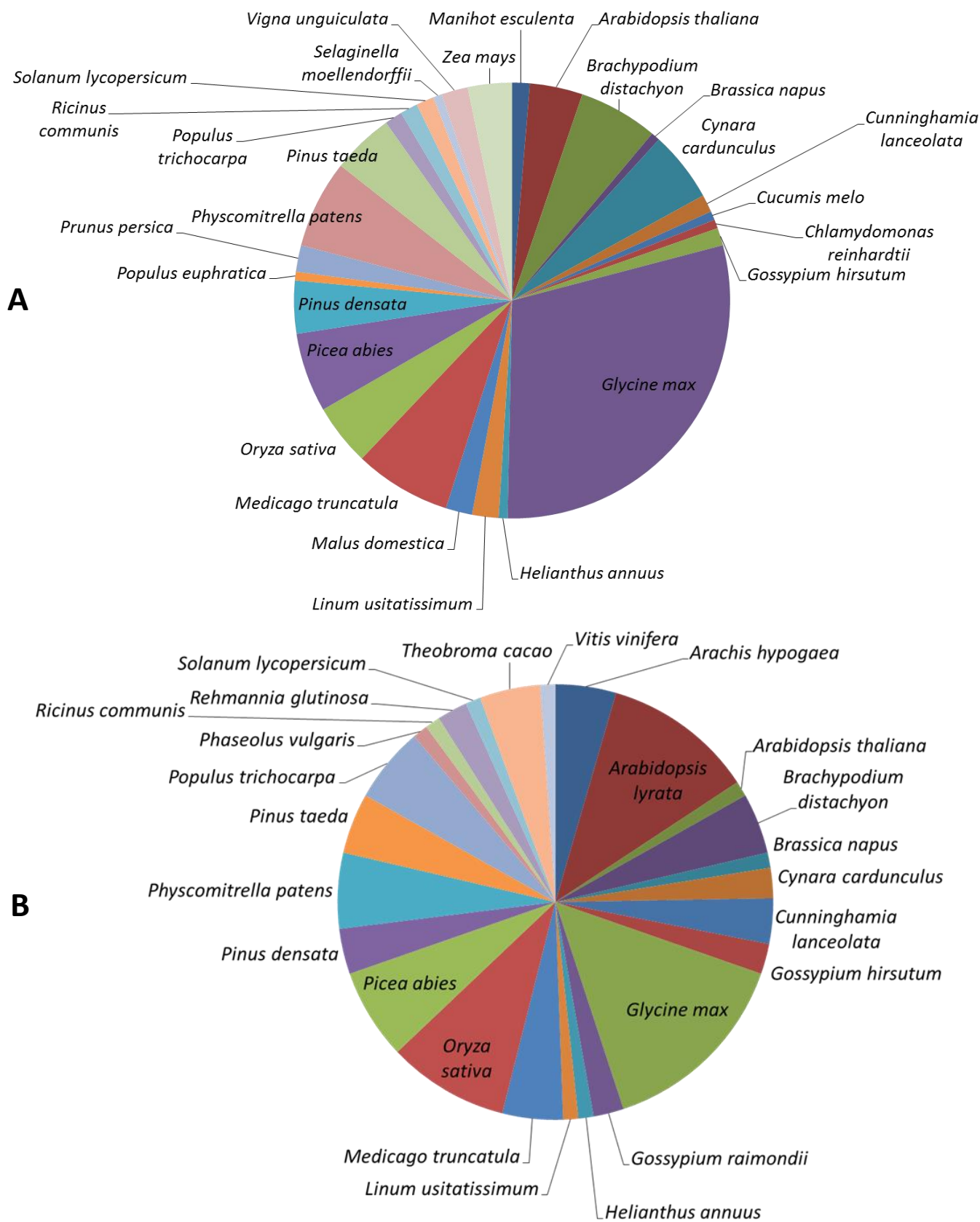

Supplement: Supplementary file 11 [file Image3.PDF]
