# Supplementary material for: A genome-wide identification of the miRNAome in response to salinity stress in date palm (Phoenix dactylifera L.)
Source: Front Plant Sci. 2015 Nov 5;6:946. doi: 10.3389/fpls.2015.00946 (PMC4633500; doi:10.3389/fpls.2015.00946)
Supplement: Supplementary file 12 [file Image4.PDF]

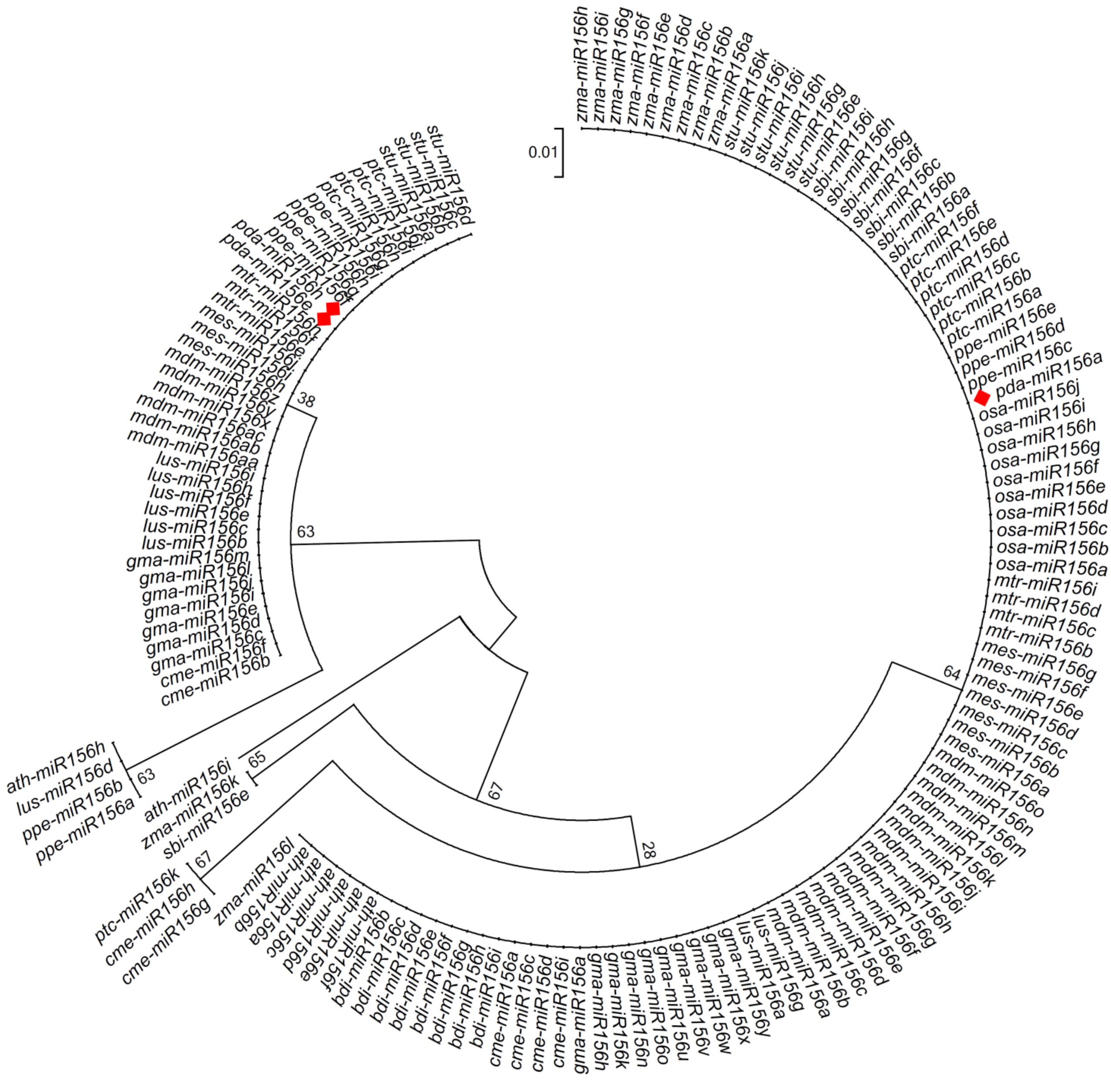

Figure S4. Phylogenetic analysis of the miRNA-156 sequences. The phylogenetic tree showed the relationship between the miRNA sequenced from date palm (marked in red color) and those previously sequenced from other plant species.
