# Supplementary material for: A genome-wide identification of the miRNAome in response to salinity stress in date palm (Phoenix dactylifera L.)
Source: Front Plant Sci. 2015 Nov 5;6:946. doi: 10.3389/fpls.2015.00946 (PMC4633500; doi:10.3389/fpls.2015.00946)
Supplement: Supplementary file 17 [file Image9.PDF]

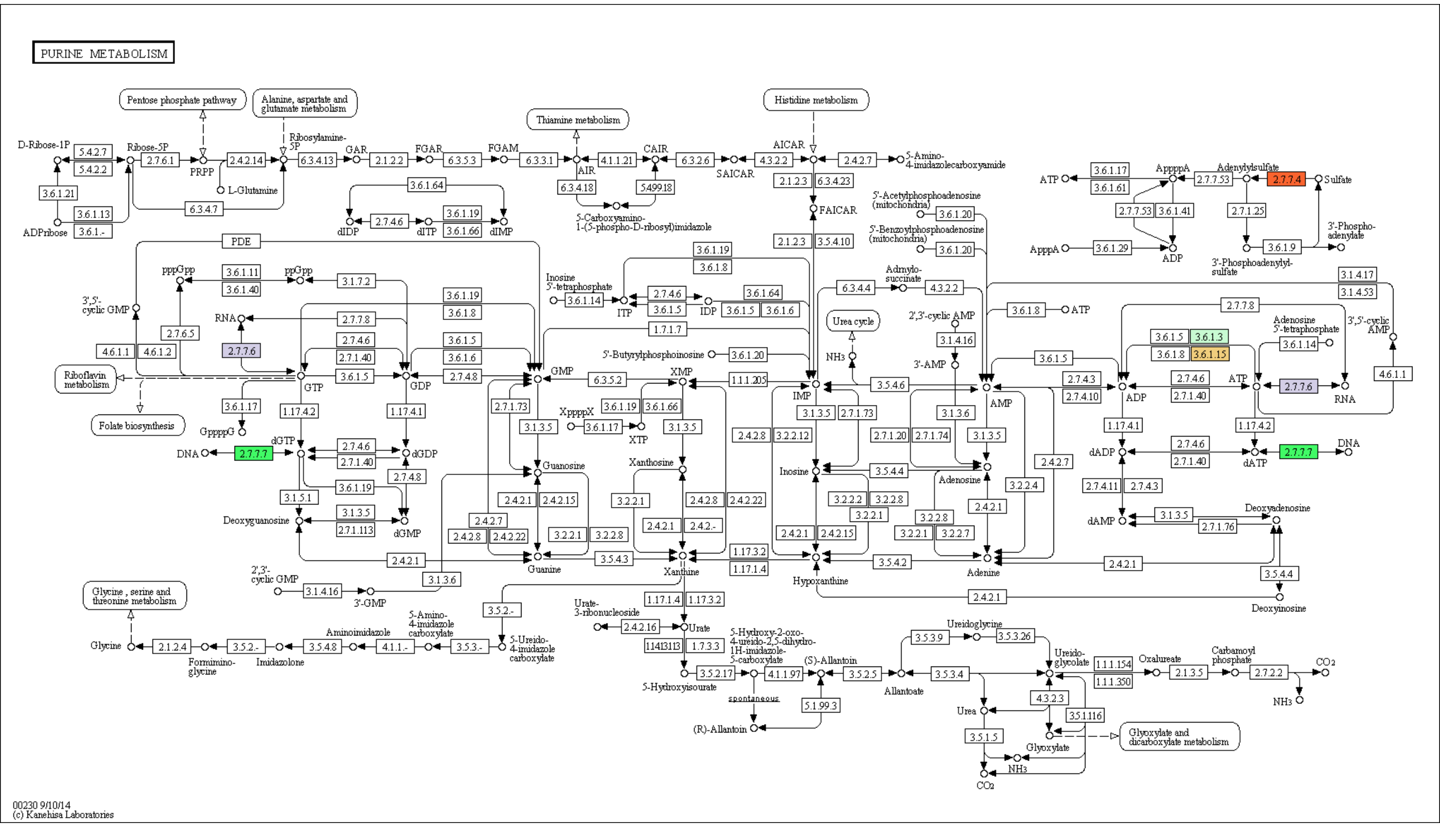

Figure S9. Purine metabolic pathway that shows the position of potential targets for miRNA isolated from *P. dactylifera* roots using the Kyoto Encyclopaedia of Genes and Genomes (KEGG).
