# Supplementary material for: A genome-wide identification of the miRNAome in response to salinity stress in date palm (Phoenix dactylifera L.)
Source: Front Plant Sci. 2015 Nov 5;6:946. doi: 10.3389/fpls.2015.00946 (PMC4633500; doi:10.3389/fpls.2015.00946)
Supplement: Supplementary file 14 [file Image6.PDF]

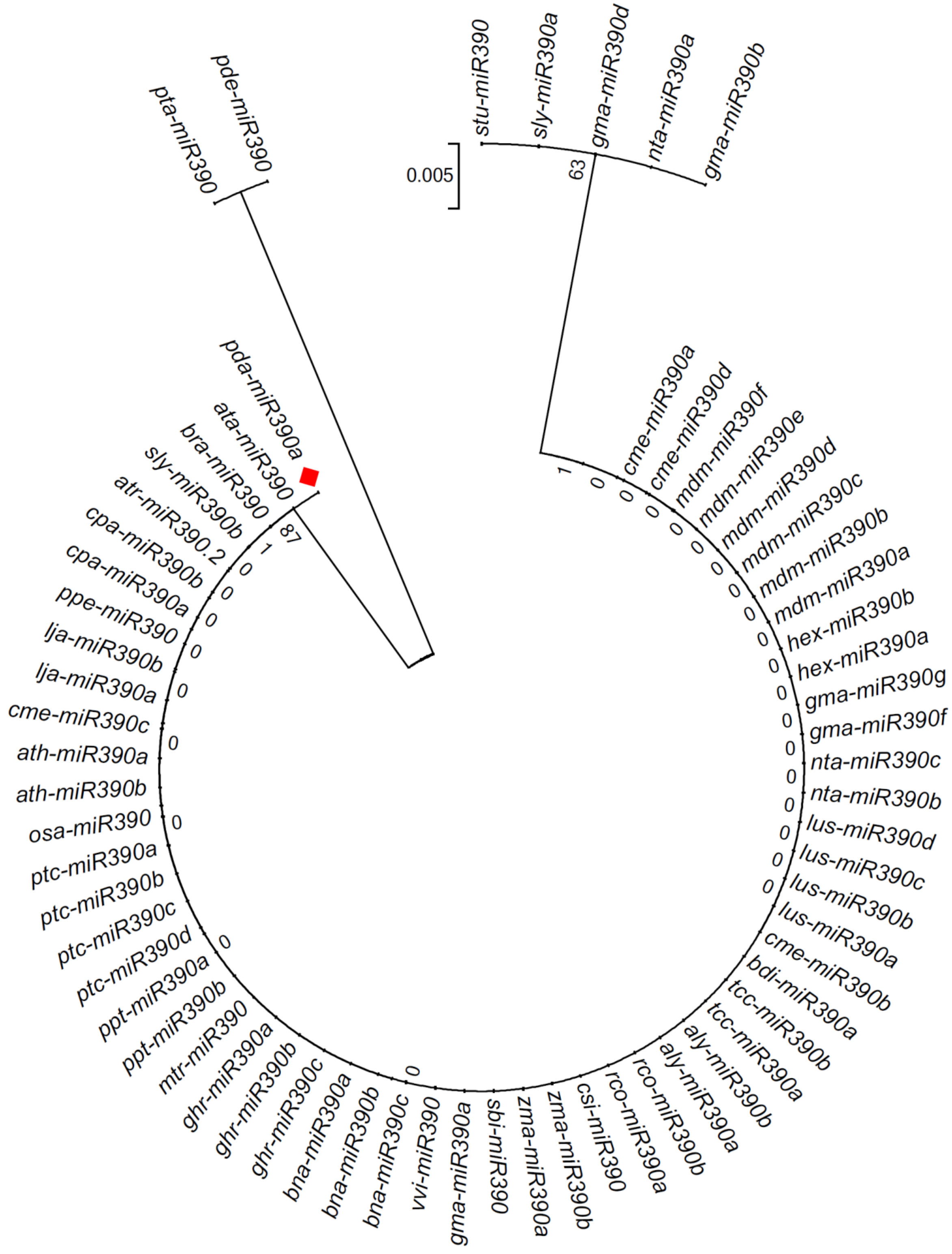

Figure S6. Phylogenetic analysis of the miRNA390 sequences. The phylogenetic tree showed the relationship between the miRNA sequenced from date palm (marked in red colour) and those previously sequenced from other plant species.
